# Sleep measurement and clinical impact in osteoarthritis and inflammatory arthritis: a scoping review

**DOI:** 10.1093/rheumatology/keag412

**Published:** 2026-08-11

**Authors:** Annalisa De Lucia, Yeliz Prior, Gianluca Bertoni, Benedetto Giardulli, Filippo Recenti, Valeria Donisi, Cinzia Perlini, Richard Jones, Andrea Dell’Isola, Simone Battista

**Affiliations:** Centre for Human Movement and Rehabilitation, School of Health and Society, University of Salford, Salford, Greater Manchester, UK; Section of Clinical Psychology, Department of Neurosciences, Biomedicine and Movement Sciences, University of Verona, Verona, Italy; Centre for Human Movement and Rehabilitation, School of Health and Society, University of Salford, Salford, Greater Manchester, UK; Department of Neurosciences, Rehabilitation, Ophthalmology, Genetics, Maternal and Child Health, University of Genoa, Campus of Savona, Savona, Italy; Centre for Human Movement and Rehabilitation, School of Health and Society, University of Salford, Salford, Greater Manchester, UK; Department of Neurosciences, Rehabilitation, Ophthalmology, Genetics, Maternal and Child Health, University of Genoa, Campus of Savona, Savona, Italy; Department of Neurosciences, Rehabilitation, Ophthalmology, Genetics, Maternal and Child Health, University of Genoa, Campus of Savona, Savona, Italy; Section of Clinical Psychology, Department of Neurosciences, Biomedicine and Movement Sciences, University of Verona, Verona, Italy; Section of Clinical Psychology, Department of Neurosciences, Biomedicine and Movement Sciences, University of Verona, Verona, Italy; Centre for Human Movement and Rehabilitation, School of Health and Society, University of Salford, Salford, Greater Manchester, UK; Department of Clinical Sciences Lund, Clinical Epidemiology Unit, Orthopaedics, Lund University, Lund, Sweden; Centre for Human Movement and Rehabilitation, School of Health and Society, University of Salford, Salford, Greater Manchester, UK

**Keywords:** sleep disturbances, osteoarthritis, inflammatory arthritis, sleep assessment methods, quality of life

## Abstract

**Objectives:**

To map how sleep has been assessed in osteoarthritis (OA) and/or inflammatory arthritis (IA), and to synthesize the evidence on the prevalence, characteristics and relationships between sleep disturbances and these conditions.

**Methods:**

We conducted a scoping review following the JBI guidelines and reported it following the PRISMA extension guidelines for scoping reviews (PRISMA-ScR). Medline (PubMed), EMBASE, CINAHL, Cochrane Central, PsycINFO, grey literature sources and ClinicalTrials.gov were searched. Using the RU-SATED framework, we categorized and narratively synthesized sleep dimensions and assessment methods and tracked the clinical impact of sleep disturbances in OA and IA.

**Results Two hundred and eighty-three:**

records (265 published studies; 18 registered trials) were included. They comprised 171 studies on IA, 77 on OA and 17 on mixed populations, which were considered separately where appropriate. Sleep assessment methods varied across studies, favouring subjective over objective measurements. Studies primarily focused on sleep satisfaction, duration, efficiency and sleepiness, overlooking regularity and timing. Global sleep disturbances were observed in ∼40–90% of OA and IA participants, with prevalence estimates varying substantially according to definitions, assessment methods and study populations. A consistent pattern of discrepancies between subjective and objective sleep metrics was identified.

**Conclusion:**

Sleep disturbance is a prevalent and clinically significant feature of both OA and IA, yet it remains under-represented in research and routine care. This review highlights substantial heterogeneity in sleep assessment and consistent discrepancies between subjective and objective measures. These findings support the need for standardized, multimethod approaches and for integrating sleep evaluation into rheumatology research and clinical guidelines.

Rheumatology key messages

- Sleep appears to be more disturbed in OA and IA than in the general population.
- Sleep issues consistently relate to pain, fatigue and higher inflammatory disease activity.
- Subjective–objective discrepancies highlight the need for multimethod sleep assessment.

## Introduction

Sleep is fundamental to physical and mental health, influencing pain perception, immune regulation and daily functioning [1, 2]. Yet, sleep disturbances are highly prevalent across a wide range of long-term conditions [3, 4]. Among these, Osteoarthritis (OA) and Inflammatory Arthritis (IA), such as Rheumatoid Arthritis (RA), Psoriatic Arthritis (PsA) and Axial Spondyloarthritis (AxSpA), are leading causes of disability and impaired quality of life worldwide [5, 6]. These conditions represent the most common forms of arthritis and share key clinical features, such as chronic pain and fatigue, which are closely linked to sleep disturbances [7]. Difficulty initiating or maintaining sleep or waking too early affects approximately one-third of the general population [8], compared with up to 80% of individuals with OA, ∼70% of those with RA, and ∼54% of those with AxSpA [7]. This markedly higher prevalence may be associated with the complex interplay between sleep, pain, fatigue and disease activity [9].

However, sleep remains under-represented in international clinical guidelines for OA and IA [10–13]. Research in this area remains fragmented and heterogeneous, reflecting variations in both assessment methods and the specific sleep dimensions investigated [14]. This heterogeneity limits comparability across studies, complicates the interpretation of findings, and hinders the systematic synthesis of evidence on the role and impact of sleep disturbances in OA and IA. In such circumstances, where quantitative synthesis is challenging, a scoping review is particularly valuable, as it enables the systematic mapping of existing evidence, identification of key concepts and clarification of how sleep has been assessed in the literature [15]. Despite growing recognition of sleep disturbances in OA and IA, there is currently no comprehensive synthesis of how different dimensions of sleep are measured, how assessment methods vary and how these differences affect findings across studies. The RU-SATED sleep health framework [16, 17] offers a foundation for such a synthesis, encompassing six dimensions: Regularity, Satisfaction, Alertness/Sleepiness, Timing, Efficiency and Duration [16].

We conducted this scoping review using the RU-SATED framework to map how sleep is assessed in OA and/or IA, while examining the prevalence, characteristics and relationships between sleep disturbances and these conditions. The RU-SATED framework was selected because it provides a multidimensional, health-oriented model of sleep, capturing both subjective and objective aspects of sleep [16]. These dimensions are particularly relevant in OA and IA, where symptoms such as pain, fatigue and inflammation may affect multiple aspects of sleep health. This approach enables the identification of key methodological gaps and provides a foundation for improving the assessment and integration of sleep in future research and clinical care.

## Methods

This scoping review was conducted following the JBI methodological guidance [18, 19] and reported according to the Preferred Reporting Items for Systematic Reviews and Meta-Analyses extension for Scoping Reviews (PRISMA-ScR) [20]. Its protocol was previously published [21].

### Eligibility criteria

Eligibility criteria were established according to the Population, Concept and Context (PCC) framework proposed by the JBI and reported in Table 1 [18].

**Table 1:** Eligibility criteria based on the population, concept and context framework.

	Inclusion criteria	Exclusion criteria
Population	Adults (≥18 years) with a clinical or instrumental diagnosis of OA and/or IA (i.e. RA, PsA and AxSpA).	Individuals younger than 18 years. Studies lacking a clinical or instrumental diagnosis of OA and/or IA.
	OA and/or IA had to represent the main condition of interest in the study.	Studies in which OA and/or IA were not the primary condition investigated.
	In studies addressing broader rheumatic or musculoskeletal conditions, specific subgroups with OA and/or IA had to be clearly identified.	Studies where it was not possible to clearly distinguish or extract specific OA and/or IA subgroups from other diagnoses.
Concept	All types of articles reported on primary studies, both quantitative and qualitative, as well as study protocols*.	Reviews^b^, editorials, commentaries, expert opinions, letters to editors, case reports, case series, book review chapters and conference abstracts.
	Studies assessing sleep as a primary or secondary outcome.	Studies in which sleep was not assessed as a primary or secondary outcome.
	Studies measuring sleep with any validated outcome measures (objective or subjective)^a^.	Studies evaluating sleep with nonvalidated outcome measures (e.g. single-item, *Ad hoc* or nonvalidated questions on sleep, qualitative studies) unless used in combination with a validated instrument.
Context	No restrictions were applied regarding publication year, country, setting or language^c^.	–

* When multiple publications appeared to derive from the same or overlapping study samples, the most appropriate record was retained, generally the most recent or the most comprehensive publication, based on relevance and completeness of information.

a ‘Validated outcome measures’ refer to tools with evidence of psychometric validation. For subjective measures (e.g. PSQI, PROMIS-SD and RU-SATED), this includes published data on reliability and validity; for objective measures (e.g. actigraphy), validation refers to evidence of concordance with polysomnography.

b Reviews were excluded as study types; however, their reference lists were screened to ensure that potentially eligible primary studies were not missed.

c In cases where studies were published in languages not spoken by the research team (English, Italian, Turkish, French, Spanish and Swedish), translation using a large language model (e.g. ChatGPT) was pre-specified in the protocol. No included studies required translation.

### Search strategy and information sources

A literature search was conducted in Medline (via PubMed), Embase, CINAHL, PsycINFO and the Cochrane Central Register of Controlled Trials (CENTRAL) through 3 June 2025. Search strategies (Supplementary Data S1) were adapted from PubMed syntax using Polyglot [22] and reviewed by an experienced librarian at the University of Salford. The grey literature was searched using the *Grey Matters* tool, a structured checklist for grey literature searching that includes sources such as government and health organization websites [23]. ClinicalTrials.gov was consulted to identify registered or ongoing trials. Finally, the reference lists of included papers and relevant reviews retrieved during the search were manually screened for additional eligible studies.

### Study selection

All retrieved records were imported into *Covidence* software [24] for duplicate removal and screening. AI-assisted translation was specified as a contingency approach for languages not covered by the review team to support inclusiveness during study selection and full-text assessment; however, no included studies ultimately required its use. Two reviewers (A.D.L. and G.B.) independently screened titles and abstracts, followed by full-text assessment of potentially eligible articles. S.B. was consulted in case of disagreement.

### Data extraction and synthesis

Data were extracted from the selected articles using an adapted JBI Standardised Data Extraction Form [18, 19]. Due to the large number of eligible studies, five authors (A.D.L., G.B., B.G., F.P. and S.B.) were involved in data extraction. A.D.L. double-checked data extracted by G.B., B.G. and S.B., while S.B. double-checked data extracted by A.D.L. and F.R. The following information was extracted: bibliographic details; country of origin; study design; population characteristics (including type of diagnosis, sample size, age, sex and gender); intervention type, comparator and related details, where applicable; sleep dimensions assessed according to the RU-SATED framework; sleep architecture and continuity outcomes; and methods of sleep assessment. The RU-SATED framework was applied as an analytical lens to map reported sleep measures to its domains; studies were not assumed to align a priori with this framework, and discrepancies or gaps were identified during synthesis. We classified patient-reported tools (e.g. PROMs) as subjective sleep assessment methods and assessments conducted using external monitoring devices (e.g. polysomnography and actigraphy) as objective sleep assessment methods. Sleep assessment tools were mapped to one or more RU-SATED domains based on the constructs they measure; multidimensional instruments were disaggregated and assigned to multiple domains where appropriate. A single sleep assessment tool could measure more than one RU-SATED sleep health dimension at the same time. The extracted data were collated and synthesized narratively [25].

## Results

### Study selection and identification process

**Figure 1:**
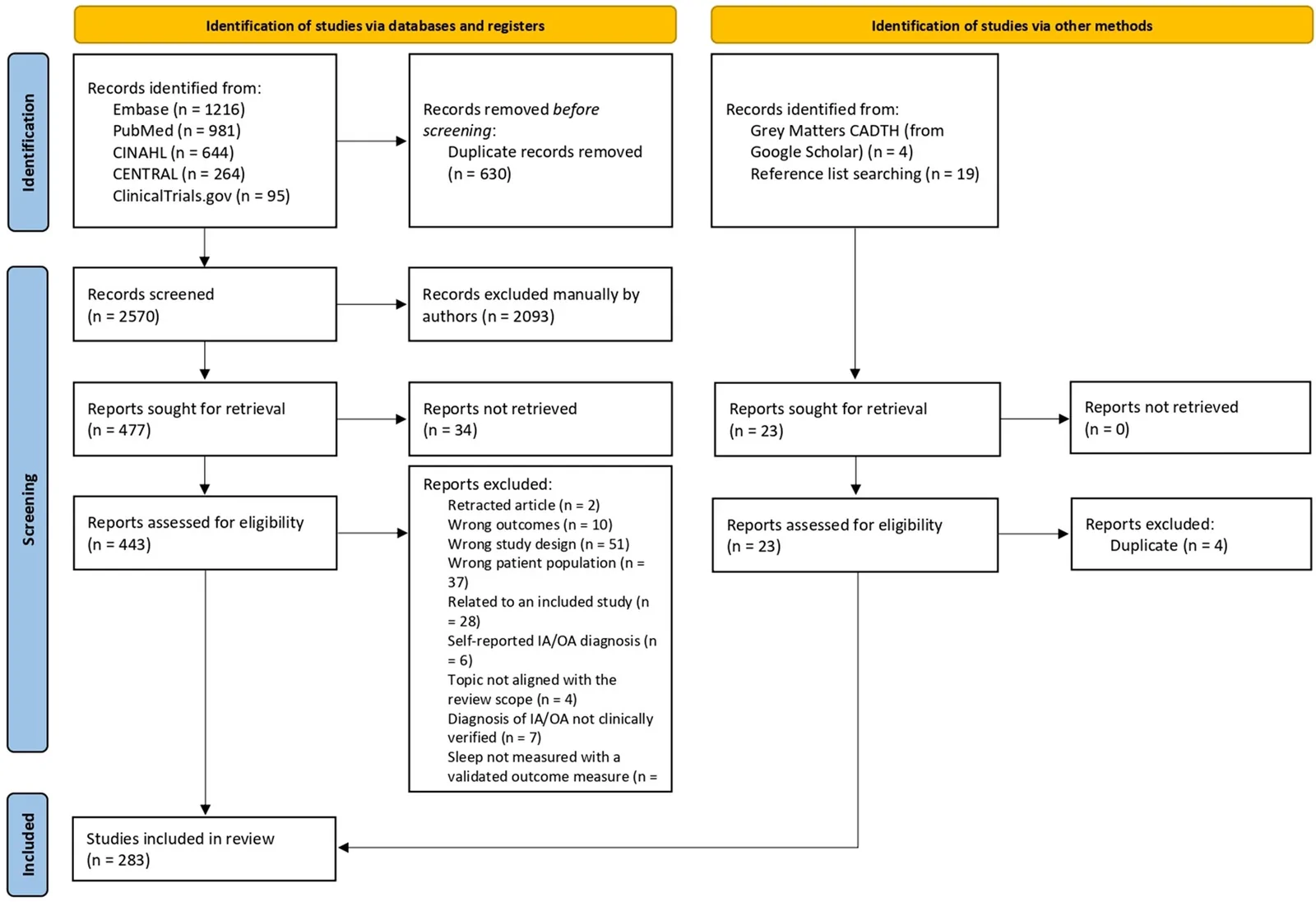
PRISMA 2020 flow diagram illustrating the identification, screening and inclusion process of studies in the scoping review

The database search yielded 3200 records, of which 283 records were included, comprising 265 published articles and 18 registered clinical trial protocols identified through ClinicalTrials.gov (Fig. 1). The list of excluded studies and corresponding reasons is in Supplementary Table S1. Full references of all included published studies are available in Supplementary Data S2. Supplementary Data S3 provides all information on the included protocols retrieved from ClinicalTrials.gov.

### Characteristics of the published studies

The included 265 studies were published between 1988 and 2025. Before 2005, publications were sporadic (≤3 studies per year), but a steady increase was observed from 2010 onwards. The number of studies peaked between 2015 and 2023. Recent years have continued to show high research output (22 studies in 2024 and 17 in 2025).

**Figure 2:**
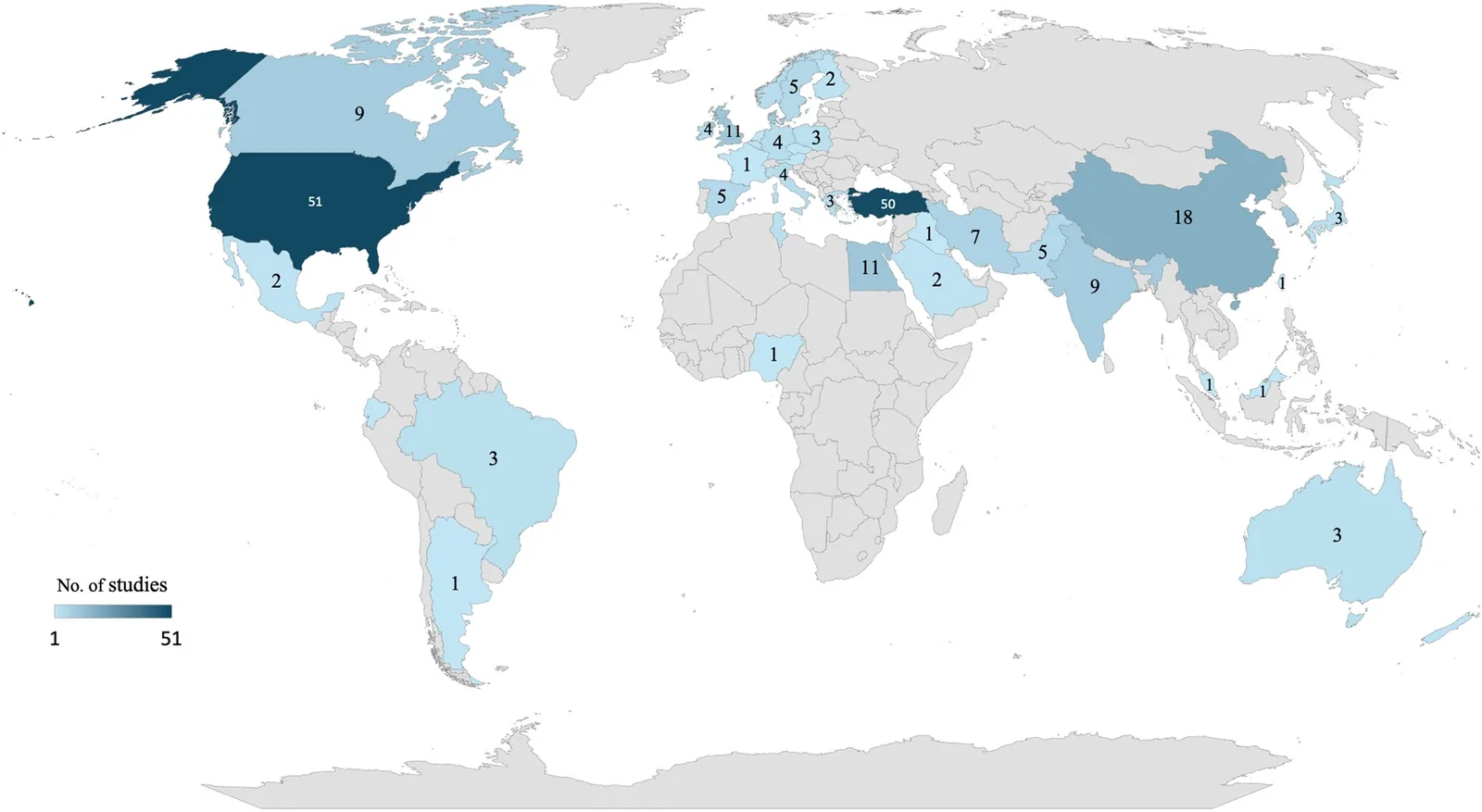
Geographical Distribution of the Publications around Sleep in OA and IA

The studies originated from 39 countries worldwide (see Fig. 2). Research activity was concentrated in high- (*n* = 153/59.3%) and upper-middle-income regions (*n* = 66/25.6%), based on the World Bank Data Help Desk [26]. Most studies were conducted in Europe (*n* = 61), North-Central America (*n* = 62) and Asia (*n* = 110), with minimal representation from Africa (*n* = 15), Oceania (*n* = 5) and South America (*n* = 5). Seven additional studies were multinational, and their country-level data were either aggregated or not fully reported.

Across the 265 published papers, sample sizes ranged from 6 to 10 040 participants. The median sample size was ∼110 participants. The most common design was cross-sectional (130/265; 49.1%), followed by randomized controlled trials (RCTs) (49/265; 18.5%), of which 8 were pilot or feasibility RCTs, cohort studies (30/265; 11.3%), and case-control studies (30/265; 11.3%). Detailed information on study characteristics is provided in Supplementary Table S2.

### Population characteristics of the included studies

Age was provided in 261 out of 265 studies (98.5%). Among studies reporting mean *±* SD, the mean of the reported means was 53.4 years (range: 27.3 ± 8.7–85.0 ± 6.0 years). Studies reporting medians (22/265; 8.4%) spanned from 34 years (IQR, 27–46) to 75 years (IQR, 70–76.5).

Reporting of sex and gender was also widespread but methodologically inconsistent. Among studies providing this information (258/265; 97.4%), the mean proportion of female participants was 64.7%, with a few studies including male-only samples. Ten studies (3.9%) used the terms *sex* and *gender* interchangeably; 22 (8.5%) reported proportions of ‘male/female’ or ‘men/women’ without clarifying whether this referred to sex or gender; and 70 (27.1%) explicitly used gender as a synonym for sex. The most frequently investigated population was individuals with RA, reported in 110 (41.5%) studies, followed by OA in 77 studies (29.1%) and AxSpA in 61 studies (21.9%). Detailed clinical characteristics are provided in Supplementary Data S4.

### Investigated sleep dimensions and measurements

Most studies (245/265; 92.5%) assessed at least one of the six dimensions proposed by the RU-SATED framework [16, 17, 27]. Overall, sleep efficiency, duration and satisfaction were the most frequently explored domains, while timing and regularity were rarely investigated.

The Pittsburgh Sleep Quality Index (PSQI) [28] was the most frequently used tool, with a cut-off score >5, to identify global sleep disturbance in prevalence studies, followed by the MOS Sleep Scale (MOS-SS) [29, 30] and the Insomnia Severity Index (ISI) [31] for insomnia severity. The term ‘global sleep disturbances’ usually refers in the literature to the overall burden of self-reported sleep disturbance or impaired sleep quality, typically assessed using the aforementioned multidimensional subjective instruments rather than formally diagnosed sleep disorders [28–30, 32]. Although the PSQI cut-off was generally similar across prevalence studies, its screening and measurement properties can vary across populations and clinical contexts. Supplementary Table S3 reports the outcome measures used across studies, along with the corresponding cut-offs where available. Subjective measures were administered alone or in combination with other self-reported instruments, and in some studies, also alongside objective assessments.

Polysomnography (PSG) was used in 30 studies (11.3%), 19 of which incorporated self-report measures, and in a few cases, actigraphy-based recordings. PSG protocols encompassed both full in-laboratory overnight studies and validated home-based or ambulatory systems [33, 34]. The most frequently reported parameters of sleep continuity included total sleep time (TST; the total duration of sleep, expressed in min or h, during the recording period), sleep onset latency [SOL; the interval between ‘lights out’ and the first epoch of any sleep stage (typically N2 on PS) expressed in min], sleep efficiency (SE; the percentage of total sleep time relative to the total time spent in bed), wake after sleep onset (WASO; the cumulative duration of wakefulness occurring after initial sleep onset), and the number of awakenings (the count of discrete transitions from sleep to wake during the recording period, often included in sleep fragmentation indices).

Sleep architecture was described as percentages or min spent in stages N1 (light sleep), N2 (intermediate/stable sleep), N3/N4 or slow-wave sleep (SWS; deep sleep), and REM. Additional indices commonly examined included the arousal index (AI; the number of EEG-defined arousals per h of sleep), the periodic limb movement index (PLMI; the number of periodic limb movements per h), the apnoea–hypopnea index (AHI; the number of apnoea, namely complete cessations of airflow ≥10 s, and hypopneas, that is partial airflow reductions ≥50% associated with ≥3% desaturation or arousal per h of sleep), used to classify the severity of obstructive sleep apnoea (OSA) or periodic limb movement disorder (PLMD).

Some studies also examined microstructural and physiological parameters, such as alpha–delta intrusions, REM density, EEG spectral analyses and oxygenation metrics (e.g. mean SpO₂, oxygen desaturation index). Actigraphy or wearable activity tracking was *employed* in 28 studies (10.6%). Devices were typically worn for several consecutive nights or weeks, in home and inpatient settings, either independently or in conjunction with self-report questionnaires and, less frequently, PSG. Commonly derived parameters included TST, SOL, SE, WASO, and indices of movement or fragmentation.

Table 2 summarizes subjective and objective sleep assessment tools across studies. Further details for all sleep assessment tools used across the included studies and registered trials are provided in Supplementary Tables S4 and S5 and Supplementary Data S3 (Table S2c and d), respectively.

**Table 2:** Overview of the sleep dimensions and measurements across the included studies and registered trials.

Name, author	No. of studies/registered trials using the tool	Sleep dimensions assessed	Format/Scoring	Psychometric properties
Subjective measures				
PSQI [28]	160/11	Sleep quality, latency, efficiency, duration, disturbances, sleeping medication use and daytime dysfunction.	Nineteen self-rated items (plus 5 nonscored bed-partner items); 4-point Likert scale; reference period: past month. global score 0–21 (higher = poorer sleep quality).	*α* = 0.70–0.85; moderate–high inter-item correlations; good construct/discriminant validity; widely validated across languages and clinical contexts [35].
ESS [36]	39/6	Daytime sleepiness; propensity to doze in everyday situations.	Eight self-rated items; 4-point Likert scale (0–3); no fixed time reference (‘recent times’). Total score 0–24 (higher = greater daytime sleepiness); cut-off ≥10 indicates excessive sleepiness.	*α* = 0.73–0.90 (mean 0.82); test–retest ICC = 0.81–0.93.
ISI [31]	26/4	Insomnia symptoms (difficulty falling asleep, maintaining sleep, early awakening, satisfaction).	Seven self-rated items; 5-point Likert scale (0–4 per item); reference period: past two weeks. Total score 0–28 (higher = more acute symptoms of insomnia).	*α* = 0.74, item–total correlations 0.36–0.54 (original validation); pooled *α* = 0.83 (95% CI 0.81–0.85) in meta-analysis (33 studies, *N* = 29 688), confirming good internal consistency across populations (Cerri *et al.* 2023) [37].
MOS-SS [29, 38]	21/1	Sleep disturbance, adequacy, somnolence, snoring, quantity and global sleep problem index.	Twelve items; 6-point Likert scale; reference period: past 4 weeks. Global score 0–100 (higher = poorer sleep).	*α* ≥ 0.73 for all subscales (except somnolence *α* = 0.63); *α* = 0.78–0.83 for summary indices (Hays *et al.* 2005). Demonstrated good construct validity and responsiveness to clinical change. Validated in general and clinical populations.
MOS Sleep Scale—Revised (MOS Sleep-R) [39]	1/0	Sleep disturbance, adequacy, somnolence, snoring, awakening short of breath/headache and sleep quantity.	Twelve items (5-point Likert); 1- or 4-week recall; T-scores (*M* = 50, SD = 10; higher = better sleep).	*α* = 0.76–0.90 and ICC ≥ 0.70 across subscales; good construct and known-groups validity across clinical conditions (sleep apnea, RA and OA).
Berlin Questionnaire [40]	6/0	Risk of obstructive sleep apnoea (OSA) (3 categories: snoring/apnoea; daytime sleepiness/fatigue; hypertension/BMI)	Ten self-rated items (Likert + yes/no); usual sleep; frequent symptoms in ≥ 2 categories = high risk	*α* = 0.86–0.92; sensitivity = 0.86, specificity = 0.77, positive predictive value = 0.89 for RDI > 5 [40]. Subsequent studies confirmed acceptable predictive accuracy against polysomnography and home sleep tests [41] supporting its wide use in clinical and epidemiological screening.
AIS [42, 43]	5/1	Insomnia symptoms according to ICD-10 diagnostic criteria: sleep induction, awakenings during the night, early morning awakening, total sleep duration, sleep quality, frequency and duration of sleep difficulties, daytime functioning and distress caused by insomnia.	Eight self-rated items (a shorter 5-item version also available); 4-point Likert scale (0–3 per item); reference period: past month. Total score 0–24 (higher = more severe insomnia); cut-off ≥ 6 suggests clinical insomnia	*α* = 0.87–0.89; test–retest reliability *r* = 0.88–0.89; strong convergent validity with the Sleep Problems Scale (*r* = 0.85–0.90). Validated in adults aged 18–79 years, both clinical and control samples.
PROMIS™ SD/SRI [44, 45]	5/0	SD: sleep initiation, maintenance, satisfaction; SRI: alertness, concentration, fatigue.	Full banks = 27 (SD) and 16 (SRI) items; short forms = 8 items each (5-point Likert, past 7 days); T-scores (*M* = 50, SD = 10).	Reliability ≥ 0.90; *r* = 0.96–0.98 vs full bank; strong convergent validity; better precision than traditional scales. Validated in adults from general and clinical populations.
Jenkins Sleep Evaluation Questionnaire (JSEQ) [46]	4/1	Sleep initiation, maintenance, early awakenings and non-restorative sleep.	Four self-rated items (a three-item version also available); 6-point Likert scale (0–5; higher = more frequent sleep problems). Reference period: past month.	*α* = 0.63–0.79; test–retest reliability *r* = 0.59.
Karolinska Sleep Diary (KSD) [47]	3/0	Latency, awakenings, duration, efficiency, quality, calmness, refreshedness (daily assessment)	Twelve daily items (5-point scale); reference period: previous night.	Internal consistency *r* = 0.46–0.83; perceived ‘sleep quality’ index (*r$\bar{\text{r}}$* = 0.77) aligned with PSG-measured sleep continuity (<4% vs >24% waking).
Leeds Sleep Evaluation Questionnaire (LSEQ) [48, 49]	2/1	Getting to sleep, quality of sleep, awakening from sleep and behaviour following wakefulness.	Ten visual analogue items (100 mm VAS); reference period: previous night.	*α* = 0.70–0.85; stable test–retest reliability; robust four-factor structure confirmed by factor analysis. strong construct and criterion validity with objective and subjective sleep indices. Sensitive to the effects of sedative, stimulant and antidepressant drugs. Validated in >40 languages.
Chronic Pain Sleep Inventory (CPSI)/Sleep Problems Index (CPSI-3) [50–52]	2/0	Pain-related sleep problems: trouble falling asleep, awakenings from pain, sleep medication use, overall quality.	Five-item VAS (0–100 mm); reference period: the past week. Mean of items 1, 3, 4 = Sleep Problems Index (CPSI-3).	Single-factor structure (loadings > 0.80); *α* > 0.90. Moderate–high inter-item correlations (*r* ≥ 0.50); discriminant validity *vs* health scales (*r* < 0.50). Sensitive to pain-related sleep improvement in clinical trials.
Korean Sleep Scale A (KSS-A) [53]	2/0	Sleep pattern, evaluation, outcomes and causes of sleep disturbance.	Fifteen self-rated items (4-point Likert).	*α* = 0.75. Four-factor structure explaining 55.9% of variance; inter-factor *r* = 0.15–0.32. Correlated with VSH Sleep Scale (*r* = 0.71) and Sleep Satisfaction Scale (*r* = 0.51), supporting construct and criterion validity.
Spiegel Sleep Questionnaire (SSQ) [54]	2/0	Subjective sleep quality and continuity.	0–30 self-rated scale (higher = better sleep); scores: 25–30 = normal, 15–24 = disturbed, <15 = pathological.	Adequate internal consistency and content validity.
Oviedo Sleep Questionnaire [55]	1/0	Subjective sleep satisfaction; insomnia and hypersomnia.	15 clinician/self-rated items; semi-structured format based on DSM-IV and ICD-10 criteria; three subscales: satisfaction (1 item, 1–7), insomnia (9 items, 9–45), hypersomnia (3 items, 3–15); higher insomnia/hypersomnia = greater severity; higher satisfaction = better sleep	*α* = 0.77 overall; *α* = 0.88 satisfaction subscale; *r* = 0.78 with HDRS sleep index; good discriminant validity.
Basic Nordic Sleep Questionnaire (BNSQ) [56]	1/0	Sleep quality, insomnia symptoms, snoring, breathing disturbances, daytime sleepiness, sleep timing and use of hypnotics.	Twenty-seven self-rated items; 5-point Likert-type scales (e.g. 1 = never to 5 = always); reference period: previous 3 months.	*α* = 0.82–0.92 across subscales; test–retest reliability *r* = 0.82–0.92; good internal consistency and reproducibility; validated in large community samples (>3000) and across languages; robust construct and criterion validity for detecting insomnia, hypersomnia and sleep-disordered breathing symptoms.
Sleep Habit Questionnaire (SHQ) [57]	1/0	Habitual sleep behaviour and disturbances: difficulty initiating and maintaining sleep, excessive daytime sleepiness, fatigue, snoring, parasomnias, sleep satisfaction, hypnotic use.	Sixty-four self-rated items. Developed for Finnish population surveys and applied in longitudinal cohort studies of sleep and health.	Good reliability, test-retest stability and concurrent and construct validity [58–60].
STOP-Bang Questionnaire [61]	1/0	Screening of OSA risk: snoring, tiredness, observed apnoea, high blood pressure, BMI >35 kg/m², age >50 years, neck >40 cm, male gender.	Eight yes/no self-rated items; total score 0–8; cutoff ≥3 = high OSA risk; reference period: habitual sleep and daytime symptoms.	High sensitivity (84–100%) and moderate specificity (30–56%) across OSA severity levels; alternative scoring (STOP ≥ 2 + male/BMI > 35) increases specificity up to ∼85–97%; adequate internal consistency and test–retest reliability; validated in >10 000 clinical and community participants; widely adopted for perioperative and sleep-clinic screening.
Mini-Sleep Questionnaire (MSQ) [62, 63]	1/0	Sleep disturbances and daytime dysfunctions: difficulty falling asleep, mid-sleep awakenings, early awakenings, hypnotic use, restless sleep (*Sleep subscale*) and tiredness, sleepiness, morning headache, difficulty waking up (*Wake subscale*).	Nine-self-rated items; 7-point Likert scale (1 = never, 7 = always); 2 subscales: *Sleep* (5 items), *Wake* (4 items). Reference period: recent habitual sleep. Higher total scores = poorer sleep quality.	*α* ≈ 0.78 (original), *α* = 0.77 (replication); stable two-factor structure; cut-offs >16 (Sleep) and >14 (Wake), AUC > 0.80; supports validity as a brief screening tool for sleep disturbances [63].
Ford Insomnia Response to Stress Test (FIRST) [64]	1/0	Individual vulnerability to stress-related sleep disturbance (trait predisposition to insomnia).	Nine self-rated items; 4-point Likert scale (1 = not likely, 4 = very likely). Reference period: hypothetical stressful situations (trait measure). Higher scores = greater vulnerability.	*α* = 0.83; 1-factor structure; higher FIRST scores associated with lower sleep efficiency and longer sleep latency (PSG) and longer MSLT latencies (hyperarousal).
Uppsala Sleep Inventory (USI) [65]	1/0	Sleep habits, quality, insomnia symptoms, daytime functioning and related factors.	Comprehensive self-report instrument (30–89 items depending on version). Most items rated on 5-point Likert scales (‘no problems’—'severe problems’; ‘never’—'very often’), with additional quantitative and yes/no questions. Reference period: recent habitual sleep. Widely used in epidemiological and clinical studies across Scandinavian populations and various medical conditions.	Adequate internal consistency, construct and convergent validity with PSG and other subjective sleep measures [66].
(Oguri–Shirakawa–Azumi Sleep Inventory, Middle-Aged and Aged Version (OSA-MA) [67]	1/0	Sleepiness on rising, initiation and maintenance of sleep, frequent dreaming, refreshment and sleep duration.	Sixteen self-rated items grouped into five subscales; 7-point Likert scale; subscales scored 0–100 (higher = better sleep quality); scores standardized using deviation scores for comparison across subscale.	Demonstrated good reliability and validity.
10-item version of the Dsfunctional Beliefs and Attitudes About Sleep scale (DBAS-10) [68]	1/1	Dysfunctional beliefs and attitudes about sleep (e.g. negative consequences of insomnia, need for control).	Ten self-rated items (visual analogue scale 0–100 mm). Reference period: general beliefs (trait). Higher scores = more dysfunctional beliefs.	Correlation high with full DBAS (*r* ≈ 0.83), acceptable internal consistency (*α* ≈ 0.69), discriminates insomnia *vs* normals, sensitive to CBT-induced changes.
10-item version of the Functional Outcomes of Sleep Questionnaire (FOSQ-10) [69]	1/0	Impact of excessive daytime sleepiness on daily activities and quality of life. 5 domains: General Productivity, Activity Level, Vigilance, Social Outcomes and Intimacy/Sexual Relationships.	Ten self-rated items; 4-point Likert scale (1 = extreme difficulty, 4 = no difficulty). Reference period: usual daily functioning. Total score (5–20; higher = better functional status).	High correlation with FOSQ-30 (*r* = 0.96–0.97; 92–94% shared variance). Internal consistency *α* = 0.87. Sensitive to treatment-related change (large effect sizes after CPAP in OSA). Demonstrated discriminant validity between OSA patients and controls.
Steer Clear Test [70]	1/0	Sustained attention and vigilance.	Computer-based driving simulation task during a monotonous 30-min highway-driving scenario.	Dood discriminant validity between untreated OSA and controls and sensitivity to treatment-related improvements; reliable objective measure of daytime vigilance.
International Restless Legs Syndrome Study Group Rating Scale for Restless Legs Syndrome (RLSSG-RS) [71]	1/0	Severity and impact of Restless Legs Syndrome symptoms.	Ten clinician- or self-rated items (0–4 each). Reference period: past 7 days. Total score 0–40 (higher = greater severity).	*α* = 0.93: test–retest reliability *r* = 0.81. Validated against clinical severity and sleep measures; sensitive to treatment-related improvement
Consensus Sleep Diary [72]	0/1	Subjective nightly sleep parameters: bedtime, sleep onset latency (SOL), number/duration of awakenings (WASO), final awakening, time out of bed, total sleep time (TST), sleep efficiency (SE) and perceived sleep quality	Daily self-report diary completed each morning (Core version: 9 items; Expanded versions include daytime variables such as naps, caffeine/alcohol use and medication). Reference period: previous night.	Developed through expert consensus and patient focus groups; clear instructions, sixth-grade reading level; face/content validity supported; designed for use in insomnia research and clinical practice.
*Objective measures*				
Polysimnography (PSG)	30/3	Sleep stages (N1, N2, N3 + N4, REM), TST, SOL, SE, WASO, respiratory and cardiac parameters.	Multichannel physiological recording (EEG, EOG, EMG, ECG, airflow, oximetry) standardized according to the American Academy of Sleep Medicine (AASM) criteria ([34]) (previously R&K manual; [33]); includes manual scoring of sleep stages, respiratory events and arousals.	Considered the gold standard for objective sleep assessment and diagnosis of sleep disorders.
Neurophysiological/Cardiorespiratory measures				
Cardiorespiratory polygraph	1/0	Apnoea–Hypopnea Index (AHI; events/h), distinction between obstructive and central sleep apnoea, Sleep-Related Breathing Disorder (SRBD) severity (mild/moderate/severe).	Home-based overnight recordings of nasal airflow, respiratory effort, oxygen saturation and heart rate; scored by board-certified pulmonologists following AASM (2012) criteria; AHI > 5 events/h classified as pathological.	
Actigraphy devices				
Actiwatch Spectrum (Philips Respironics, Bend, OR, USA)	3/0	(a, b) Sleep fragmentation index (SFI), SOL, SE, WASO, sleep duration, no. of awakenings. © TST.	(a, b) Worn on nondominant wrist for 14 days; 30-s epochs; analysed with Actiware v6.0 (Philips Respironics); main rest intervals manually scored; ≥4 h nonwear excluded; sleep measures averaged across valid days. (c) Worn on nondominant wrist for 7 nights (mean = 6.8 days); analysed with Actiware software (Philips Respironics); automatically scored using built-in sleep–wake algorithm; concurrent recordings with MyZeo EEG and daily sleep diaries for validation.	NA
Actiwatch (Mini Mitter Company, Inc., Bend, OR, USA)	1/0	TST, SOL, SE, WASO.	Worn on nondominant wrist for seven consecutive nights (concurrent with sleep diary); piezoelectric accelerometer; 1-min epochs; analysed with Actiware v5.57 (Mini Mitter); medium sensitivity; sleep–wake automatically scored; onset/offset defined as first/last 10 min of sleep with ≤1 epoch wake.	NA
Actiwatch-2™ (Mini Mitter/Philips Respironics, Bend, OR, USA)	5/0	(a) TST, Wake Time, no. of awakenings. (b) TST, SOL, SE, WASO. (c) Circadian rest–activity rhythm (RAR) parameters—amplitude (strength of rhythm), MESOR (mean activity), acrophase (timing of peak activity) and F-statistic (robustness of rhythm). (d) SE	(a) Configured to record 1-min epochs; analysed using Actiware v5.57; bed/rise times manually entered and validated via event markers, activity and light data; sleep/wake classified using the Cole–Kripke algorithm. (b) Data averaged across 14-day recordings at multiple timepoints; analysed per standardized actigraphy methods (likely *Actiware* algorithm); continuous 24-h wear on nondominant wrist. (c) Data collected in 60-s epochs, analysed with Actiware v6.0 and SAS (extended cosinor model); ≥5 valid days required; sleep diaries used for editing but not included in RAR analyses. d) Worn on wrist for 7 consecutive nights at each assessment; recording epochs of 1 min; analysed with Actiware software; night period defined as ‘lights out’ to final wake time, using bed/rise times from daily sleep logs. Higher SE = better sleep.	NA
Actiwatch-16™ (Mini Mitter, Bend, OR, USA)	1/0	Assumed sleep duration, actual sleep time (AST), SE, sleep activity (mean activity per min during sleep), sleep fragmentation, time in bed (TIB).	(a) Worn continuously on wrist (except for bathing) for five consecutive nights pre- and postsurgery; analysed using Sleepwatch software (Mini Mitter); data collected in 1-min epochs; automatic scoring of sleep–wake states using activity thresholds; parameters derived per manufacturer algorithm. Data analysed with Actiwatch Activity & Sleep Analysis Software; higher FI = more restlessness.	NA
Actiwatch-Score™ (Philips Respironics/Mini Mitter, Bend, OR, USA)	1/0	TST, SOL, SE, WASO.	Wrist-worn accelerometer; activity recorded in 15-s epochs; data processed with onboard Actiwatch software using standardized scoring protocol; sleep intervals defined by self-reported lights-off and wake-up times corroborated with actigraphy data; adjustments applied for discrepancies >30 min between diary and activity data.	NA
Actiwatch-Score™ (Cambridge Neurotechnology Ltd, Cambridge, UK)	2/0	(a) Movement > 0.05 g, 3–11 Hz; 30-s epochs, TST, SOL, SE, WASO. (b) SE—derived from accelerometery	(a) Data analysed with Actiwatch Activity & Sleep Analysis v5.32; automatic sleep/wake scoring per epoch. (b) Data collected in 24-h epochs; analyzed with Actiwatch proprietary software; validated *vs* PSG; SE z-scored for composite symptom index.	NA
Actiwatch (model NS, Cambridge Neurotechnology Ltd )	1/0	SE, AST, SFI.	Worn on nondominant wrist continuously for ∼14 days; 1-min epochs; analysed with Actiwatch Activity & Sleep Analysis Software using Meadows *et al.* (2005) validated algorithm; home nights only included [73].	NA
Mini Mitter Actiwatch™ (Mini Mitter Company, Inc., Bend, OR, USA)	0/1	TST, SOL, SE, WASO.	Worn on nondominant wrist for 14 consecutive days at baseline, 6-week and 12-week follow-up; omnidirectional accelerometer (≥0.01 g) recording activity counts per epoch; data analysed via built-in algorithm to classify sleep/wake; concurrent daily sleep diaries used for comparison.	NA
Actigraph 2 (Philips Healthcare/Respironics, Bend, OR)	1/0	TST, SOL, SE, WASO.	Worn on nondominant wrist for 14 days; data collected in 60-s epochs (medium sensitivity); analysed using PSG-validated algorithm for sleep continuity estimation; accompanied by daily sleep diaries.	NA
ActiGraph® GT3X-W (ActiGraph LLC, Pensacola, FL, USA)	1/0	TST, SE, WASO, no. of awakenings, sleep fragmentation.	Analysed with ActiLife® software using the Cole–Kripke algorithm (10-s epochs); sleep onset/wake derived algorithmically from accelerometer data; 7-day recording (3–7 valid nights per participant)	NA
Axivity AX3 (Axivity Ltd, Newcastle, UK)	1/0	Sleep duration, sleep onset/wake time, sedentary behaviour (<45 mg ENMO), light PA (45–100 mg ENMO), moderate-to-vigorous PA (≥100 mg ENMO), average acceleration, intensity gradient.	100 Hz sampling; ±8 g range; data processed with GGIR (v1.11-0, R); van Hees algorithm for sleep detection; ≥16 h/day wear, ≥4 valid days; calibration error ≤0.01 g	NA
Axivity AX6 (Axivity Ltd, York, UK)	1/0	Sleep duration, SE, sustained inactivity bouts (SIB), moderate-to-vigorous physical activity (MVPA).	Raw data (100 Hz, ±8 g) processed via OMGUI and GGIR v2.8-0 (R); autocalibration, filtering and van Hees algorithm used for sleep–wake detection; valid data defined as >16 h/day wear.	NA
Motionlogger Actigraph (Ambulatory Monitoring Inc., Ardsley, NY, USA)	2/0	(a) TST, SOL, SE, no. and duration of nocturnal awakenings. (b) AST, longest continuous sleep time, % sleep time, no. of awakenings.	(a) Analysed with Action-W software (AMI); automatic sleep–wake scoring; 14-day continuous recording. (b) Analysed with AW2 software (AMI) with Cole–Kripke algorithm; recordings 20:00–06:00 (worn during pre-operative and postoperative nights).	NA
Mini-Motionlogger Actigraph (Ambulatory Monitoring Inc., Ardsley, NY, USA)	1/0	Sleep/Wake states (activity-based), TST, SOL, SE, no. of awakenings.	Worn on wrist continuously for 8 days; data collected in 1-min epochs in zero-crossings mode; analysed with Action-4 software (AMI); sleep/wake scored automatically using the Cole–Kripke algorithm; event markers used to validate diary-reported bed/rise times.	NA
Mini-Act AMA-32 (Ambulatory Monitoring Inc.)	1/0	SE, sleep length, time awake at night.	Worn on nondominant hand for four nights; 1-min epochs; scoring software/algorithm not specified; used to assess sleep quality.	NA
Wrist-worn actigraph (model NS)	2/0	(a) TST, SOL, SE, WASO, no. of awakenings; (b) Wrist movement activity (frequency, intensity) during PSG.	(a) Worn on wrist for three consecutive nights; model and software NS; data collected and retrieved in-person after 3 days. (b) Continuous during PSG; model/software NS	NA
Multimodal wearable devices				
SenseWear Pro3 Armband™ (BodyMedia Inc., Pittsburgh, PA, USA)	1/0	TST, SE, WASO, MVPA duration, Sedentary Behavior (SBD), total and physical activity (PA) Energy Expenditure.	60-s epochs; analysed with BodyMedia proprietary software; automatic algorithm using accelerometery + physiological signals; ≥4 valid days with ≥95% wear time; first day excluded	NA
SenseWear Mini™ (BodyMedia Inc., Pittsburgh, PA, USA)	1/0	TST, SE, TIB, lying awake/resting, no. of steps, METs-based activity intensity (including MVPA).	Multisensor armband (upper arm, triceps of nondominant arm); worn 24 h/day for 7 days; combines accelerometery, heat flux, galvanic skin response, skin temperature; processed using SenseWear Professional Software v8.1.0.22; min-by-min scoring of sleep, sedentary, ambulation and activity intensity; exported to MATLAB for behavioral classification and aggregation across valid days (≥20 h wear/day).	NA
SenseWear Armband™ (SWA; Model MF-SW; BodyMedia Inc., Pittsburgh, PA, USA)	1/0	TST, SOL, SE, WASO, sedentary, moderate and vigorous activity levels (METs), total PA, steps and lying time.	Multisensor armband worn over triceps; integrates accelerometery, heat flux, skin temperature and galvanic skin response; analysed via SenseWear Professional Software (v8.1.0.22). Sleep–wake parameters derived per Oudegeest-Sander *et al.* (2013) during seven nights of continuous use [74].	NA
Actiheart™ (CamNTech Ltd, Cambridge, UK)	1/0	Night sleep duration (min), SB, light (LPA) and moderate-to-vigorous physical activity (MVPA); heart rate (HR) and energy expenditure (METs).	Waterproof chest-mounted monitor combining uniaxial accelerometery and ECG. Data sampled every 30 s via two ECG electrodes (Red Dot 2560, 3M) worn on the left chest for seven consecutive days. Sleep identified as prolonged low movement + low HR; sedentary time = 0 counts/min with valid HR. Data cleaned and analysed per Collings *et al.* (2014) [75].	NA
Consumer-grade wearable activity trackers (WATs)				
Fitbit Alta™ (Fitbit Inc., San Francisco, USA)	1/0	TST, SOL, SE, *n*. of awakenings, shallow/deep/REM sleep	24-h continuous recording; Fitbit proprietary algorithm; automatic stage classification (no external software)	NA
Withings PULSE HR® (Withings, Issy-les-Moulineaux, France)	1/0	TST (light and deep sleep), daily step count, time spent in light, moderate and intense activity (s/day).	Worn continuously on wrist for 52 weeks; data automatically synchronized with Withings Health Mate App®; predefined intensity thresholds based on maximum heart rate and proprietary sleep–wake algorithms; daily summaries exported for analysis.	NA
Fitbit Sense	0/1	Daily movement (e.g. the no. of steps) and sleep parameters.	Worn continuously on wrist during 14-week intervention. Data retrieved via Fitbit API and analysed using custom-made algorithms to quantify daily movement and sleep behaviours	NA

### Sleep dysfunctions in OA and IA

Findings are presented collectively where consistent across OA and IA and separately where condition-specific differences were identified. Global sleep disturbances were highly prevalent and clinically relevant in OA [76, 77], RA [78–80], PsA [81] and AxSpA [82], with reported prevalence estimates ranging widely (∼40–90%) across studies, depending on the definitions, populations and assessment methods used (see Supplementary Table S3).

PSG revealed fragmented, inefficient and lightened sleep, characterized by prolonged SOL, reduced SE and SWS, higher proportions of N1–N2 stages, and frequent arousals and PLMs (e.g. [82–84]). In RA and AxSpA, the AHI correlated with inflammatory activity, anthropometric measures (e.g. neck circumference, occiput-to-wall distance), and disease duration or age (e.g. [85, 86]).

Notably, several studies reported apparently preserved macro-architectural profiles—that is, normal proportions of NREM and REM stages—yet marked sleep fragmentation and microstructural abnormalities (e.g. frequent arousals, alpha–delta intrusions or altered spindle activity; e.g. [87–89]).

Actigraphic data likewise indicated disrupted sleep patterns, with moderately reduced efficiency, shortened TST and increased nocturnal wakefulness (e.g. [90–92]) compared with healthy controls [93–95]. SE and fragmentation were often correlated with pain, fatigue and depressive symptoms (e.g. [96–98]). More recent wearable-based research in AxSpA points to considerable inter-individual variability in sleep responses and their relationship with physical activity, although this finding is based on a limited number of studies [99].

Several comorbid primary sleep disorders were also identified, with prevalence estimates varying substantially across studies depending on ascertainment methods (e.g. formal clinical diagnoses, PSG, validated screening tools and self-reported conditions). These differences should be considered when interpreting the reported estimates.

Clinical insomnia was among the most frequently reported conditions. Studies employing validated screening tools generally reported prevalence rates ranging from ∼17% to over 60% in OA (e.g. [100, 101]), 19% to 50% in axSpA (e.g. [102, 103]) and 40% to 75% in RA (e.g. [104, 105]). In contrast, studies based on formal diagnostic interviews or medical records tended to report lower prevalences (e.g. 13% to 33% in RA; [89, 106]). In OA, one study found that 53% of patients reported clinically significant insomnia symptoms based on the ISI, but only 22% had a corresponding ICD-9 diagnosis recorded in their electronic medical records [107].

RLS, OSA and PLMs were also reported, although prevalence estimates varied widely. RLS prevalence ranged from 2% to ∼68.4% in RA (e.g. [87, 89]), from 3.6% to ∼25% in OA (e.g. [3, 77, 108]), and reached 55% in AS [109]. OSA prevalence varied from 10.5% to 82.9% in RA (e.g. [83, 87]), was 33.7% based on diagnostic codes in OA [107], and ranged from 22.6% to 73.7% in AS [84, 110]. Finally, PLMs were measured less frequently, with prevalence ranging from 1% to 73.7% in RA [87, 89] and 26.3% in AxSpa [84]. A detailed overview of prevalence estimates, along with the corresponding diagnostic or assessment methods for each study, is provided in Supplementary Table S3. However, prevalence estimates for insomnia, OSA and RLS should be interpreted cautiously and may not be directly comparable across studies due to differences in case definitions, inclusion criteria and diagnostic or screening approaches.

Across OA and IA populations, pain and disease activity consistently emerged as the key predictors of poor sleep [111, 112]. Depression and anxiety further mediated these associations, sometimes exerting a greater influence on sleep quality than objective inflammatory indices [113, 114].

Notably, perceived symptoms and patient-reported impact seemed more strongly associated with subjective sleep disturbances than with objective disease markers. Moreover, subjective sleep assessment tools were in several cases poor predictors of objective abnormalities on polysomnography or actigraphy, revealing a recurrent discrepancy between subjective and objective measures [87, 115]. For instance, this pattern was observed in studies by Karataş *et al.* [83, 84], where in both RA and AxSpa cohorts, poorer self-reported sleep quality (PSQI) closely tracked with pain, functional disability and disease activity, but not with PSG parameters or inflammatory indices. Following anti–TNF-α therapy, patients in both cohorts reported marked subjective improvements in sleep despite unchanged PSG outcomes [83, 84]. Similar discrepancies have also been noted in behavioural and mind–body programmes for people with OA, in which improvements in self-reported sleep quality did not translate into significant actigraphic changes [116–118]. In OA, actigraphic recordings showed shortened, fragmented sleep. Interestingly, participants underestimated their objective sleep duration by about two h compared with diary estimates [119].

Evidence from interventional studies across OA and IA suggests that a range of therapeutic approaches—including anti-inflammatory treatments (particularly anti-TNF agents and, in some cohorts, tocilizumab), exercise and mind–body programmes (aerobic or strength training, yoga and Baduanjin), physiotherapy, acupressure/balneotherapy and cognitive behavioural therapy for insomnia (CBT-I)—can mitigate sleep disturbances and related symptoms, leading to measurable improvements in PSQI scores. These improvements were typically accompanied by reductions in pain, fatigue and depressive symptoms; however, objective sleep indices often showed little change [120, 121]. A summary of intervention characteristics across included interventional studies is provided in Supplementary Table S6.

In OA surgical cohorts, sleep quality typically worsened immediately after joint replacement and then gradually returned to preoperative (baseline) levels as pain and function recovered [122]. Prospective studies consistently demonstrate this pattern: following total knee or hip arthroplasty, sleep efficiency and PSQI scores deteriorate sharply within the first postoperative weeks [122–124], then progressively normalize alongside pain relief and functional improvement within 3–12 months [125, 126].

## Discussion

This scoping review provides an updated and comprehensive overview of how sleep has been investigated in individuals with OA and IA. The synthesis highlights a growing scientific interest in this topic, indicating that sleep is increasingly recognized as a relevant outcome in rheumatologic research, particularly as a key patient-reported outcome (PRO) identified by OMERACT [127, 128]. Moreover, it shows that global sleep disturbances and primary sleep disorders (e.g. insomnia, OSA and RLS) are generally more prevalent among individuals with OA and IA compared with controls, although estimates vary across studies [76, 78, 81–84, 93, 95] and are frequently associated with greater pain, fatigue, depressive symptoms, higher BMI and markers of inflammatory activity [85, 107, 110].

Nevertheless, the body of evidence remains highly heterogeneous in study designs, sample sizes, clinical populations, measurements and outcomes, as observed in this review, underscoring the need for greater methodological consistency and more standardized reporting in future research. Most studies were cross-sectional, involved small to moderate sample sizes, and relied primarily on subjective self-report instruments to assess perceived sleep quality and related aspects. Objective assessments, such as PSG and actigraphy devices, were used in a minority of studies, mostly in small cohorts, and generally indicated fragmented, inefficient, lighter sleep.

Across all conditions, the sleep dimensions most frequently explored were satisfaction, efficiency, alertness/daytime sleepiness and duration, all of which were consistently reported as impaired across studies. In contrast, regularity and timing—key components of sleep health reflecting circadian stability—were rarely assessed. This limited coverage highlights an important gap in the literature as key inflammatory mediators such as TNF-α and IL-6 follow circadian patterns [129, 130] and are influenced by sleep–wake timing [9, 131]. Moreover, existing evidence already indicates that disrupted rest/activity rhythms are associated with less robust circadian stability in subsets of people with OA with insomnia, potentially amplifying pain sensitivity (e.g. [94]).

Across studies, a discrepancy between subjective and objective sleep indicators was observed, although the number of directly comparable studies was limited. This finding is consistent with a well-documented pattern in broader sleep research across both clinical and nonclinical populations [132–134]. Patients frequently rated their sleep as poorer and shorter than suggested by objective recordings [87, 119, 135]. Intervention studies further suggested this divergence: improvements following pharmacological or behavioural interventions were generally reflected in PROMs but not in PSG or actigraphy parameters [83, 84, 121, 136]. Conversely, objective measures sometimes identify sleep abnormalities that patients are unaware of, such as OSA and PLMs [83, 115]. Together, these findings indicate that subjective and objective metrics may capture complementary yet distinct aspects of sleep health, and that both should be integrated into OA and IA sleep research [137].

Overall, these findings highlight substantial heterogeneity and incomplete coverage of sleep dimensions across studies, underscoring the need for more consistent, multidimensional (objective and subjective) approaches to sleep assessment in future research. The limited use of objective methods has important implications: in studies relying solely on subjective measures, associations between sleep and outcomes such as pain, fatigue or disease activity may partly reflect perceptual biases rather than physiological sleep disruption. Incorporating objective approaches—now increasingly feasible through research-grade actigraphy and validated consumer wearables (e.g. smart rings and activity trackers) [138, 139]—may enhance ecological validity [140] and provide a more comprehensive understanding of sleep health in individuals with OA and IA. Moreover, inconsistent use of sleep measures creates substantial heterogeneity, making future systematic reviews and meta-analyses difficult. These findings support consideration of greater methodological consistency, including the use of widely adopted instruments (e.g. PSQI, ISI, ESS) alongside objective measures, to facilitate comparability across studies [141]. In this context, the *de novo* development of standardized core outcome sets for sleep in OA and IA, in line with initiatives such as COMET, may help improve consistency and comparability across studies [142].

### Limitations

While the inclusion of both peer-reviewed and grey literature broadened the scope of the evidence base, the heterogeneity of study designs, assessment tools and outcome reporting limited the comparability of findings across studies. Furthermore, although restricting the synthesis to validated sleep measures enhanced methodological consistency, this decision may have excluded clinically relevant studies that used nonvalidated tools. Composite disease-specific instruments (e.g. RAID and PSAID), which include single sleep-related items, may not have been consistently captured or classified as sleep assessment tools in this review. Finally, a deviation from the protocol was conducted due to the large number of studies: instead of two people extracting the data, we divided the work among five authors. However, all the data extracted were checked by two authors.

## Conclusions

Sleep disturbance is a common and clinically relevant feature of both OA and IA. This scoping review highlights substantial heterogeneity in how sleep is assessed across studies, with a predominance of subjective measures and incomplete coverage of key sleep dimensions, particularly within the RU-SATED framework. This variability limits comparability across studies and constrains the synthesis of evidence. Our findings underscore the need for more consistent and multidimensional approaches to sleep assessment, including integrating validated subjective tools with objective measures. Greater methodological alignment, potentially supported by the development of core outcome sets and arthritis-specific sleep-related PROMs, may enhance the quality and comparability of future research and support more effective integration of sleep into rheumatology care and guidelines.

## Supplementary Material

keag412_Supplementary_Data

## Data Availability

The data that support the findings of this study are available in the supplementary material of this article.

## Individual support

Annalisa De Lucia attended the PhD program (Cycle XXXVIII) in Neuroscience, Psychological, and Psychiatric Sciences, and Movement Sciences at the University of Verona, Italy. Her scholarship was financed by Ministerial Decree 351 of April 9, 2022, based on the National Recovery and Resilience Plan (PNRR), funded by the European Union (NextGenerationEU program), Mission 4 (‘Education and Research’), component 1 (‘Enhancement of the offer of educational services: from nurseries to universities’) as part of the following investments: investment 3.4 (‘Teaching and advanced academic skills’; project M4C1: CUPB31I22000190006) and investment 4.1 (‘Extension of the number of research doctorates and innovative doctorates for public administration and cultural heritage’; project M4C1: CUP31I22000200006).
