# Supplementary material for: Sleep measurement and clinical impact in osteoarthritis and inflammatory arthritis: a scoping review
Source: Rheumatology (Oxford). 2026 Aug 11;65(8):keag412. doi: 10.1093/rheumatology/keag412 (PMC13523860; doi:10.1093/rheumatology/keag412)
Supplement: Supplementary file 1 [file keag412_supplementary_data.zip › rhe-25-3089-File006.docx]

**Supplementary Data S3**

**Characteristics of the Included Registered Trials**

The following section provides a detailed overview of the registered clinical trials included in the review.

A total of 18 registered clinical trials were included. The studies were initiated between 2006 and 2024, revealing a clear increase in registrations over the past decade. At the time of data extraction, four trials were active and recruiting, two were active but no longer enrolling, three were completed, and two had completed recruitment. Five trials had not yet started recruitment, while two presented an unknown or unupdated status.

The trials were conducted across ten countries, most frequently in Turkey (n=3), China (n=3), and Brazil (n=3), followed by India (n=2), Belgium (n=1), the Netherlands (n=1), the USA (n=1), Japan (n=1), Egypt (n=1), and Thailand (n=1).

The study designs were predominantly randomised controlled trials (RCTs) (n = 13), with the remaining studies employing cohort or case–control designs. Sample sizes varied widely, ranging from 8 to 1,000 participants, although most trials enrolled fewer than 100 individuals.

The clinical populations primarily included people with OA and RA, often in association with comorbid insomnia in a subset of studies. A smaller number of trials focused on knee or hip OA or on AS. Across trials, both males and females were typically included, with age ranges spanning from 18 to 80 years, covering adult and older adult populations.

Among the registered studies, only one trial (NCT00374556) had results publicly available on ClinicalTrials.gov. This RCT evaluated eszopiclone versus placebo in patients with OA-related insomnia (n=29). Sleep was assessed using subjective diaries, which captured TST, SOL, SE, WASO, TIB, the number of awakenings, and sleep quality (ISI), alongside actigraphy. No corresponding peer-reviewed publication was identified.

The tables below summarize the main characteristics of the included trials, the interventions evaluated, the sleep measures used, and the targeted sleep outcomes.

**Table S2a.** Characteristics of the included registered trials

| **Identifier, year** | **Country** | **Population** | | **Sample size** | | **Sex/Gender distinction reported (Yes/No)** | **Sex** | | **Age (years)** | |
| --- | --- | --- | --- | --- | --- | --- | --- | --- | --- | --- |
|  |  | **I** | **C** | **I** | **C** |  | **Male** | **Female** | **I** | **C** |
| ***Cohort studies*** | | | | | | | | | | |
| NCT06430918, 2024 | Turkey | KOA | NA | 150 | NA | Yes | NS | NS | Adults and older adults | NA |
| NCT00287794, 2006 | Japan | RA | RA | 1000 | | Yes | NS | NS | ≥ 17 | |
| ***Case-control study*** | | | | | | | | | | |
| NCT04809168, 2021 | Turkey | AS | Nociceptive/mechanical pain (> 3 months) | 292 | | Yes | NS | NS | 18-70 | |
| ***RCTs*** | | | | | | | | | | |
| NCT05861934, 2022 | Turkey | RA | RA | 45 | | Yes | NS | NS | Range: 18-90 | |
| NCT05387473, 2022 | Belgium | KOA + insomnia diagnosis (DSM-5 criteria) | KOA + insomnia diagnosis (DSM-5 criteria) | 64 | 64 | Yes | NS | NS | ≥ 45 | |
| NCT04728581, 2024 | Netherlands | KOA | KOA | 165 | | Yes | NS | NS | ≥ 50 | |
| NCT00374556, 2006 | USA | OA + chronic insomnia | OA + chronic insomnia | 14 | 15 | Yes | 10 | 19 | 54.6 ± 5.2 | 52.9 ± 6.6 |
| NCT01919645, 2012 | Brazil | RA | RA | 43 | | Yes | NA | NS | 35-65 | |
| NCT03606707, 2018 | Egypt | RA | RA | 60 | | Yes | NS | NS | 20-80 | |
| NCT04059419, 2019 | Brazil | OA | OA | 30 | 30 | Yes | NS | NS | ≥ 70 | |
| NCT06527547, 2024 | China | RA | RA | 26 | 26 | Yes | NS | NS | 18-72 | |
| ChiCTR-IOR-16007858, 2016 | China | OA or RA | OA or RA | 85 | 85 | Yes | NS | NS | 22-88 | |
| CTRI/2021/02/031562, 2021 | India | Hip OA | Hip OA | 80 | 80 | Yes | NS | NS | 18-80 | |
| CTRI/2024/09/073634, 2024 | India | KOA | KOA | 40 | 40 | Yes | NS | NS | 40-60 | |
| ISRCTN61333726, 2025 | China | KOA | KOA | 88 | | Yes | NS | NS | ≥ 50 | |
| RBR-69cyr2, 2019 | Brazil | RA | RA | 8 | 8 | Yes | NA | NS | 18-100 | |
| RBR-8pjqh2, 2018 | Brazil | RA | RA | 26 | 27 | Yes | NS | NS | 18-80 | |
| TCTR20240425005, 2024 | Thailand | RA + insomnia | RA + insomnia | 84 | | Yes | NS | NS | ≥ 18 | |

AS, Ankylosing Spondylitis; C, Control; DSM-5, Diagnostic and Statistical Manual of Mental Disorders, 5th Edition; I, Intervention; KOA, Knee Osteoarthritis; NA, Not Applicable; NS, Not Specified; OA, Osteoarthritis; RA, Rheumatoid Arthritis; RCT, Randomised Controlled Trial.

**Table S2b.** Intervention characteristics of the included registered trials

| **Identifier, year** | **Intervention** | | | **Comparator** | | | **Key findings** |
| --- | --- | --- | --- | --- | --- | --- | --- |
|  | **Type** | **Duration** | **Other characteristics** | **Type** | **Duration** | **Other characteristics** |  |
| NCT05861934, 2022 | Distant Reiki application | 6 biweekly sessions | // | Routine care and treatment | // | // | NA |
| NCT05387473, 2022 | CBTi-UC: 3 sessions of education/advice (weeks 1–2), then 1×/week exercise + 1×/week CBT-I (weeks 3–5), then alternating weekly CBT-I or exercise (weeks 6–10), and finally 1×/week exercise (weeks 11–14) | 14 weeks | // | UC + information sessions: 3 sessions of education/advice (weeks 1–2), then 1×/week exercise + 1×/week information sessions (weeks 3–5), then alternating weekly information or exercise (weeks 6–10), and finally 1×/week exercise (weeks 11–14) | 14 weeks | // | NA |
| NCT04728581, 2024 | Group 1: 3.75 mg mirtazapine nightly, with option to increase to 7.5 mg Group 2: 3.125 mg quetiapine nightly, with option to increase to 6.25 mg | Over 2 weeks after TKA | // | Placebo (gelatinous capsule without an active ingredient) | Over 2 weeks after TKA | Group 1: 3.75 mg mirtazapine nightly, with option to increase to 7.5 mg Group 2: 3.125 mg quetiapine nightly, with option to increase to 6.25 mg | NA |
| NCT00374556, 2006 | Eszopiclone 3mg capsules | 12 weeks, once/day at bedtime | // | 3mg placebo capsules | 12 weeks, once/day at bedtime | // | Eszopiclone improved some sleep diary measures (WASO, sleep quality, SE, TST) and ISI scores at 6 weeks, but effects were inconsistent at 12 weeks. Pain and QoL showed limited change |
| NCT00287794, 2006 | Diazepam, melatonin | NR | // | NA | NA | NA | NA |
| ChiCTR-IOR-16007858, 2016 | Hypnotics | NR | // | No hypnotics | NR | // | NA |
| CTRI/2021/02/031562, 2021 | Trazodone 50mg | 1 month, once/day, from 2 days pre-op up to 4 weeks post-op | // | Starch capsule 50 mg (placebo) | 1 month, once/day | // | NA |
| CTRI/2024/09/073634, 2024 | Mindfulness-based program (Pilates, body scan, sitting/walking meditation, mindful stretching) | 6 weeks, 3 sessions/week, 50 min each | // | Guidelines based physiotherapy treatment (isometrics, progressive strengthening, stretching with sandbags, mini-squats, aerobic training + warm-up/cool-down and home exercise program) | 4 weeks, 3 supervised sessions/week + 2 home sessions/week, daily walking 15–30 min | // | NA |
| ISRCTN61333726, 2025 | Auricular acupressure (AA) applied to six specific acupoints with Vaccaria seeds | Applied once on the day of surgery, then patients self-pressed taped points 4–5 times/day (3–5 minutes per session) for 7 consecutive days | // | Sham AA with blank adhesive tape applied to the same acupoints | Same schedule as IG | // | NA |
| NCT01919645, 2012 | Combined Training (strength + aerobic) | NR (assessment at 8 and 16 weeks) | // | Sleep hygiene information | Monthly follow-up phone calls, assessments at weeks 8 and 16, plus 2 visits to get and then return the actigraph | // | NA |
| NCT03606707, 2018 | Intra-articular steroid injection + methotrexate and chloroquine 400 mg/day | 3 months | // | Systemic steroid 20 mg/day + methotrexate and chloroquine 400 mg/day | 3 months | // | NA |
| NCT04059419, 2019 | Multiprofessional program (PARQVE): knee OA education; nutrition counselling; psychological group therapy; supervised physiotherapy sessions; physical educator sessions | Education: 2 days of lectures (2 months apart) Hospital nutrition consultations at months 1, 3, and 5 Psychological group therapy sessions at months 4 and 6 7 supervised sessions with the physical therapy team + 7 sessions with the physical educators’ team (weekly ×4 weeks, then every 2 weeks ×3) | // | UC | Assessment at baseline, 3 and 6 months | // |  |
| NCT06527547, 2024 | Transcutaneous auricular vagus nerve stimulation (taVNS) with Huatuo electronic acupuncture device (SDZ-IIB). Electrodes placed on auricular points for heart and kidney | 4 weeks, 30-min home self-treatment (after physician training), twice daily, 5 days/week | // | Sham stimulation using the same Huatuo electronic acupuncture device (model SDZ-II) | Same schedule as IG | // | NA |
| RBR-69cyr2, 2019 | AA at specific sleep points | 8 weekly sessions | // | AA at non-specific points | Same schedule as IG | // | NA |
| RBR-8pjqh2, 2018 | Melatonin (5mg) as fast-release capsules | 90 days, 1 capsules/day | // | Placebo (capsules without an active ingredient) | Same schedule as IG | // | NA |
| TCTR20240425005, 2024 | Group 1: mindfulness- based intervention group therapy Group 2: group CBT | 4 bi-weekly sessions | // | Patient education about RA, self-care, nutrition, exercise and sleep hygiene | Same schedule as IG | // | NA |

CBT-I, Cognitive Behavioural Therapy for Insomnia; IG, Intervention Group; NA, Not Applicable; TKA – Total Knee Arthroplasty; UC, Usual Care.

**Table S2c.** Summary of self-reported sleep measures and Ru-SATED dimensions covered across included registered trials

| **Identifier, year** | **Self-reported outcome measures (Yes/No)** | **RU-SATED Sleep Health Dimensions** | | | | | | **Other (specify)** | **Tool(s) used (specify)** |
| --- | --- | --- | --- | --- | --- | --- | --- | --- | --- |
|  |  | **Regularity** | **Satisfaction** | **Alertness/Sleepiness** | **Timing** | **Efficiency** | **Duration** |  |  |
| NCT05861934, 2022  NCT04809168, 2021  NCT03606707, 2018  CTRI/2024/09/073634, 2024  ISRCTN61333726, 2025 | Yes | No | Yes | Yes | No | Yes | Yes | Sleeping medicine use, disturbances in sleep | PSQI |
|  |  |  |  |  |  |  |  |  |  |
|  |  |  |  |  |  |  |  |  |  |
|  |  |  |  |  |  |  |  |  |  |
|  |  |  |  |  |  |  |  |  |  |
| NCT06430918, 2024 | Yes | No | No | Yes | No | Yes | No | Total sleep disturbance score | JSEQ |
| NCT04728581, 2024 | Yes | No | Yes | Yes | No | Yes | No | Sense of balance and coordination upon awakening | LSEQ |
| TCTR20240425005, 2024 | Yes | No | Yes | Yes | No | Yes | Yes | // | Modified AIS |
| NCT04059419, 2019 | Yes | No | Yes | Yes | No | Yes | Yes | Insomnia severity | ISI, ESS |
| RBR-69cyr2, 2019  RBR-8pjqh2, 2018  CTRI/2021/02/031562, 2021 | Yes | No | Yes | Yes | No | Yes | Yes | Sleeping medicine use, disturbances in sleep | PSQI, ESS |
|  |  |  |  |  |  |  |  |  |  |
|  |  |  |  |  |  |  |  |  |  |
| ChiCTR-IOR-16007858, 2016 | Yes | No | Yes | Yes | No | Yes | Yes | Sleeping medicine use, disturbances in sleep, insomnia severity | PSQI, ISI |
| NCT05387473, 2022 | Yes | No | Yes | Yes | No | Yes | Yes | Sleeping medicine use, disturbances in sleep, insomnia severity, dysfunctional beliefs about sleep | PSQI, ESS, ISI, DBAS |
| NCT00374556, 2006 | Yes | No | Yes | No | No | Yes | Yes | TIB (min), WASO, no. of awakenings, insomnia severity | Daily Sleep Diary, VAS, ISI |
| NCT06527547, 2024 | Yes | No | Yes | Yes | No | Yes | Yes | Sleeping medicine use, disturbances in sleep; frequency of snoring, awakening short of breath or with a headache, and of naps, sleep problems index; time into bed, time out of bed | PSQI, MOS-SS, ESS, consensus sleep diary |
| NCT00287794, 2006  NCT01919645, 2012 | No |  |  |  |  |  |  |  |  |
|  |  |  |  |  |  |  |  |  |  |

AIS, Athens Insomnia Scale; DBAS, Dysfunctional Beliefs and Attitudes about Sleep; ESS, Epworth Sleepiness Scale; ISI, Insomnia Severity Index; JSEQ, Jenkins Sleep Evaluation Questionnaire; LSEQ, Leeds Sleep Evaluation Questionnaire; MOS-SS, Medical Outcomes Study – Sleep Scale; PSQI, Pittsburgh Sleep Quality Index.

**Table S2d.** Summary of objective sleep measures and sleep architecture/continuity outcomes covered across the included registered trials

| **Identifier, year** | **Sleep Architecture** | | | | | **Sleep Continuity** | | | | | | | **Other** | **Tool(s) used** |
| --- | --- | --- | --- | --- | --- | --- | --- | --- | --- | --- | --- | --- | --- | --- |
|  | **NREM (% or min)** | | | **REM (% or min)** | **REM latency (min)** | **Total Sleep Time (TST, min)** | **Timing** | **Sleep latency (SL, min)** | **Sleep efficiency (SE, %)** | **Wake After Sleep Onset (WASO, min)** | **No. of awakenings** | **Sleep Fragmentation Index (SFI)** |  |  |
|  | **N1** | **N2** | **N3+N4/Slow-Wave Sleep** |  |  |  |  |  |  |  |  |  |  |  |
| NCT05387473, 2022 | Yes | Yes | Yes | Yes | No | Yes | No | Yes | Yes | Yes | No | No | TIB, respiratory parameters, myoclonic activity | Fitbit Sense, PSG |
| NCT00374556, 2006 | No | No | No | No | No | Yes | No | Yes | Yes | Yes | No | No | // | Actigraphy (Mini Mitter Actiwatch) |
| ChiCTR-IOR-16007858, 2016 | NR | NR | NR | NR | NR | NR | NR | NR | NR | NR | NR | NR | // | PSG |
| NCT01919645, 2012 | No | No | Yes | No | No | No | No | No | No | No | No | No | // |  |

PSG, Polysomnography; TIB, Time in Bed.

**References**

1. ClinicalTrials.gov. (2024). *Neuropathic Pain and Its Relation to Sleep Quality in Knee Osteoarthriti*s. (Identifier NCT06430918). U.S. National Library of Medicine. https://clinicaltrials.gov/study/NCT06430918
2. ClinicalTrials.gov. (2022). *The Effect of Distant Reiki Application on Pain, Functional Status and Sleep Quality*. (Identifier NCT05861934). U.S. National Library of Medicine. https://clinicaltrials.gov/study/NCT05861934
3. ClinicalTrials.gov. (2022). *Added Value of Cognitive Behavioural Therapy for Insomnia in Persons With Knee Osteoarthritis (PREMEO).* (Identifier: NCT05387473). U.S. National Library of Medicine. https://clinicaltrials.gov/study/NCT05387473
4. ClinicalTrials.gov. (2021). *Neuropathic Spinal Pain in Ankylosing Spondylitis.* (Identifier: NCT04809168). U.S. National Library of Medicine. https://clinicaltrials.gov/study/NCT04809168
5. ClinicalTrials.gov. (2024). *Improving Sleep After TKA Using Mirtazapine and Quetiapine*. (Identifier: NCT04728581). U.S. National Library of Medicine. https://clinicaltrials.gov/study/NCT04728581
6. ClinicalTrials.gov. (2006). *Insomnia and Osteoarthritis Study*. (Identifier: NCT00374556). U.S. National Library of Medicine. https://clinicaltrials.gov/study/NCT00374556
7. ClinicalTrials.gov. (2006). *Quality of Sleep in Patients With Rheumatoid Arthritis.* (Identifier: NCT00287794). U.S. National Library of Medicine. https://clinicaltrials.gov/study/NCT00287794
8. ClinicalTrials.gov. (2012). *Effectiveness and Safety of Physical Exercises in the Improvement of the Sleep Quality*. Regarding Rheumatoid Arthritis (SRA). (Identifier: NCT01919645). U.S. National Library of Medicine. https://clinicaltrials.gov/study/NCT01919645
9. ClinicalTrials.gov. (2018). *Efficacy of Fluoroscopic Guided Atlantoaxial Joint Injection on Head and Neck Pain and Sleep Quality in RA Patients.* (Identifier: NCT03606707). U.S. National Library of Medicine. https://clinicaltrials.gov/study/NCT03606707
10. ClinicalTrials.gov. (2019)*.* *PARQUE 70+ Studying Sleep Disorder.* (Identifier: NCT04059419). U.S. National Library of Medicine. https://clinicaltrials.gov/study/NCT04059419
11. ClinicalTrials.gov. (2024). *Effect of Transcutaneous Auricular Electroacupuncture on Sleep Disturbances in Patients With Rheumatoid Arthritis: A Randomized Double-Blind Clinical Trial*. (Identifier: NCT06527547). U.S. National Library of Medicine. https://clinicaltrials.gov/study/NCT06527547
12. Registro Brasileiro de Ensaios Clínicos (ReBec). (2019). *Auriculotherapy in sleep disorders in patients with rheumatoid Arthritis*. (Identifier: RBR-69cyr2). https://ensaiosclinicos.gov.br/rg/RBR-69cyr2
13. Registro Brasileiro de Ensaios Clínicos (ReBec). (2018). *Effects of Melatonin on clinical manifestations, inflammation and sleep quality in patients with Rheumatoid Arthritis.* (Identifier: RBR-8pjqh2). https://ensaiosclinicos.gov.br/rg/RBR-8pjqh2
14. Thai Clinical Trials Registry (TCTR). (2024). *The effect of mindfulness-based intervention and cognitive-behavioral therapy on disease activity and sleep quality in patients with rheumatoid arthritis and concomitant insomnia: a randomized controlled trial*. (Identifier: TCTR20240425005). https://www.thaiclinicaltrials.org/show/TCTR20240425005
15. The UK's Clinical Study Registry. (2025). *Study on the effectiveness of auricular acupressure in improving sleep after knee surgery*. (Identifier: ISRCTN61333726) https://www.isrctn.com/ISRCTN61333726
16. Chinese Clinical Trial Registry (ChiCTR). (2016). *Sleep quality affects early knee function recovery and pain relief following total knee arthroplasty*. (Identifier ChiCTR-IOR-16007858). https://www.chictr.org.cn/showprojEN.html?proj=13278
17. Clinical Trials Registry – India (CTRI). (2021). *A randomized-controlled trial to look for sleep pattern of patients operated with hip* *replacement surgery after taking a sleeping pill (trazodone)*. (Identifier: CTRI/2021/02/031562). https://ctri.nic.in/Clinicaltrials/pmaindet2.php?EncHid=NDk1Njg=&Enc=&userName=
18. Clinical Trials Registry – India (CTRI). (2024). *Effect Of Mind-Body Technique on Sleep Quality, Pain and Disability in Knee Osteoarthritis Patients- A Randomized Controlled Trial*. (Identifier: CTRI//2024/09/073634). https://ctri.nic.in/Clinicaltrials/pmaindet2.php?EncHid=MTEyNzc5&Enc=&userName=
