# Supplementary material for: Sleep measurement and clinical impact in osteoarthritis and inflammatory arthritis: a scoping review
Source: Rheumatology (Oxford). 2026 Aug 11;65(8):keag412. doi: 10.1093/rheumatology/keag412 (PMC13523860; doi:10.1093/rheumatology/keag412)
Supplement: Supplementary file 1 [file keag412_supplementary_data.zip › Supplement_Data.zip!/rhe-25-3089-File004.docx]

**Supplementary Data S1**

**Search strategies**

**PubMed**

(osteoarthritis[Mesh] OR osteoarthritis[TIAB] OR osteoarthrosis[TIAB] OR arthrosis[TIAB] OR arthritis[Mesh] OR "inflammatory arthritis"[TIAB] OR arthritis[TIAB] OR arthropathy[TIAB] OR "Arthritis, Rheumatoid"[Mesh] OR "Rheumatoid Arthritis"[TIAB] OR spondylitis[TIAB] OR Spondylarthritis[Mesh] OR "Non-Radiographic Axial Spondyloarthritis"[Mesh] OR "Axial Spondyloarthritis"[Mesh] OR "Spondylitis, Ankylosing"[Mesh] OR spondylarthritis[TIAB] OR "Non-Radiographic Axial Spondylarthritis"[TIAB] OR "Arthritis, Psoriatic"[Mesh] OR "Psoriatic Arthritis"[TIAB]) AND ("Sleep Arousal Disorders"[Mesh] OR "Sleep Duration"[Mesh] OR "Sleep Quality"[Mesh] OR "Sleep Latency"[Mesh] OR "Sleep Hygiene"[Mesh] OR Parasomnias[Mesh] OR Sleep[Mesh] OR "Sleep assessment"[TIAB] OR "Sleep efficiency"[TIAB] OR "Sleep latency"[TIAB] OR "Sleep fragmentation"[TIAB] OR "Sleep architecture"[TIAB] OR sleep[TI])

*Results: 963*

**Cochrane Central**

#1 MeSH descriptor: [Osteoarthritis] explode all trees

#2 (osteoarthritis OR osteoarthrosis):ti,ab,kw (Word variations have been searched)

#3 (arthrosis OR "inflammatory arthritis" OR arthritis OR arthropathy):ti,ab,kw (Word variations have been searched)

#4 MeSH descriptor: [Arthritis] explode all trees

#5 MeSH descriptor: [Arthritis, Rheumatoid] explode all trees

#6 ("Rheumatoid Arthritis"):ti,ab,kw (Word variations have been searched)

#7 (spondylitis):ti,ab,kw (Word variations have been searched)

#8 MeSH descriptor: [Spondylarthritis] explode all trees

#9 MeSH descriptor: [Non-Radiographic Axial Spondyloarthritis] explode all trees

#10 MeSH descriptor: [Axial Spondyloarthritis] explode all trees

#11 MeSH descriptor: [Spondylitis, Ankylosing] explode all trees

#12 (spondylitis):ti,ab,kw (Word variations have been searched)

#13 ("Non-Radiographic Axial Spondylarthritis"):ti,ab,kw (Word variations have been searched) #14 MeSH descriptor: [Arthritis, Psoriatic] explode all trees

#15 ("Psoriatic Arthritis"):ti,ab,kw (Word variations have been searched)

#16 #1 OR #2 OR #3 OR #4 OR #5 OR #6 OR #7 OR #8 OR #9 OR #10 OR #11 OR #12 OR #13 OR #14 OR #15

#17 MeSH descriptor: [Sleep Arousal Disorders] explode all trees

#18 MeSH descriptor: [Sleep Duration] explode all trees

#19 MeSH descriptor: [Sleep Quality] explode all trees

#20 MeSH descriptor: [Sleep Latency] explode all trees

#21 MeSH descriptor: [Sleep Hygiene] explode all trees

#22 MeSH descriptor: [Parasomnias] explode all trees

#23 MeSH descriptor: [Sleep] explode all trees

#24 ("Sleep assessment" OR "Sleep efficiency" OR "Sleep latency" OR "Sleep fragmentation" OR "Sleep architecture"):ti,ab,kw (Word variations have been searched)

#25 (sleep):ti

#26 #17 OR #18 OR #19 OR #20 OR #21 OR #22 OR #23 OR #24 OR #25

#27 #16 AND #26

*Results: 254*

**CINAHL and PsychINFO**

((MH "Osteoarthritis+") OR XB ( osteoarthritis OR osteoarthrosis ) OR (MH "Arthritis+") OR XB (arthrosis OR "inflammatory arthritis" OR arthritis OR arthropathy) OR (MH "Arthritis, Rheumatoid+") OR XB ( "rheumatoid arthritis" OR spondylitis ) OR ((MH "Spondylarthritis+") OR (MH "Non-Radiographic Axial Spondyloarthritis+") OR (MH "Axial Spondyloarthritis+") OR (MH "Spondylitis, Ankylosing+") ) OR XB ( spondylitis OR spondylarthritis OR "non-radiographic axial spondylarthritis" ) OR (MH "Arthritis, Psoriatic+") OR "psoriatic arthritis" ) AND (((MH "Sleep Arousal Disorders+") OR (MH "Sleep Duration+") OR (MH "Sleep Quality+") OR (MH "Sleep Latency+") OR (MH "Sleep Hygiene+") OR (MH "Parasomnias+") OR (MH "Sleep+")) OR XB ( "sleep assessment" OR "sleep efficiency" OR "sleep latency" OR "sleep fragmentation" OR "sleep architecture") OR TI sleep)

*Results: 715*

**Embase**

#1: ('osteoarthritis'/exp OR osteoarthritis:ti,ab OR osteoarthrosis:ti,ab OR arthrosis:ti,ab OR 'arthritis'/exp OR 'inflammatory arthritis':ti,ab OR arthritis:ti,ab OR arthropathy:ti,ab OR 'arthritis, rheumatoid'/exp OR 'rheumatoid arthritis':ti,ab OR spondylitis:ti,ab OR 'spondylarthritis'/exp OR 'non-radiographic axial spondyloarthritis'/exp OR 'axial spondyloarthritis'/exp OR 'spondylitis, ankylosing'/exp OR spondylarthritis:ti,ab OR 'non-radiographic axial spondylarthritis':ti,ab OR 'arthritis, psoriatic'/exp OR 'psoriatic arthritis':ti,ab) AND ('sleep arousal disorders'/exp OR 'sleep duration'/exp OR 'sleep quality'/exp OR 'sleep latency'/exp OR 'sleep hygiene'/exp OR 'parasomnias'/exp OR 'sleep'/exp OR 'sleep assessment':ti,ab OR 'sleep efficiency':ti,ab OR 'sleep latency':ti,ab OR 'sleep fragmentation':ti,ab OR 'sleep architecture':ti,ab OR sleep:ti)

#2: [embase]/lim NOT ([embase]/lim AND [medline]/lim)

#3: #1 AND #2

#4: #3 NOT ('conference abstract'/it OR 'conference paper'/it OR 'conference review'/it)

#5: #4 NOT ([adolescent]/lim OR [child]/lim OR [fetus]/lim OR [infant]/lim OR [newborn]/lim OR [preschool]/lim OR [school]/lim)

*Results: 1,207*

**Clinical Trial.gov**

(osteoarthritis OR osteoarthrosis OR arthrosis OR arthritis OR inflammatory arthritis OR arthropathy OR rheumatoid arthritis OR spondylitis OR spondylarthritis OR non-radiographic axial spondyloarthritis OR axial spondyloarthritis OR ankylosing spondylitis OR psoriatic arthritis) AND (sleep arousal disorders OR sleep duration OR sleep quality OR sleep latency OR sleep hygiene OR parasomnias OR sleep assessment OR sleep efficiency OR sleep fragmentation OR sleep architecture OR sleep) (Filters: Adults and older adults)

*Results: 50*
