# Supplementary material for: Sleep measurement and clinical impact in osteoarthritis and inflammatory arthritis: a scoping review
Source: Rheumatology (Oxford). 2026 Aug 11;65(8):keag412. doi: 10.1093/rheumatology/keag412 (PMC13523860; doi:10.1093/rheumatology/keag412)
Supplement: Supplementary file 1 [file keag412_supplementary_data.zip › Supplement_Data.zip!/rhe-25-3089-File005.docx]

**Supplementary Data S2**

**Detailed List of Included Published Studies**

1. Abd Elazeem, M. I., & Salem, M. N. (2018). Can rheumatoid arthritis affect sleep in Egyptian patients? *Egyptian Rheumatologist*, *40*(1), 11–16. https://doi.org/10.1016/j.ejr.2017.06.001
2. Abdelrahman, M. S. I., Shaddad, A. M., Khaleel, W. G. E., Talaat, E. A., Safwat, S. M., & Farrag, S. (2024). Sleep quality, anxiety, depression, and quality of life in rheumatoid arthritis patients and impact of disease activity. *Egyptian Rheumatology and Rehabilitation*, *51*(1). https://doi.org/10.1186/s43166-024-00242-z
3. Abdulaziez, O., & Asaad, T. (2012). Sleep problems in ankylosing spondylitis: Polysomnographic pattern and disease related variables. *Egyptian Rheumatologist*, *34*(2), 59–65. https://doi.org/10.1016/j.ejr.2012.02.001
4. Acar, Y., Ilc¸in, N., & Sari, I. (2023). The Effects of Tele-Yoga in Ankylosing Spondylitis Patients: A Randomized Controlled Trial. *Journal of Integrative and Complementary Medicine*, *29*(11), 727–737. https://doi.org/10.1089/jicm.2023.0022
5. Acharya, N. N., Venkatesan, P., Karvannan, H., & Balebail Gopalakrishna, D. (2024). Comparison of Yoga with exercise in ankylosing spondylitis on mobility and functional capacity. *International Journal of Rheumatic Diseases*, *27*(6). https://doi.org/10.1111/1756-185X.15223
6. Adawi, M., Sabbah, F., Tzischinsky, O., Blum, N., Bragazzi, N. L., Yehuda, I., Tamir, S., Romanenko, O., & Blum, A. (2020). Sleep disorders and vascular responsiveness in patients with rheumatoid arthritis. *Journal of Internal Medicine*, *288*(4), 439–445. https://doi.org/10.1111/joim.13087
7. Akintayo, R. O., Yerima, A., Uhunmwangho, C., Olaosebikan, H., & Akpabio, A. A. (2019). Tossing and turning with degenerative arthropathy: An assessment of poor sleep quality in knee osteoarthritis. *Reumatologia*, *57*(4), 207–213. https://doi.org/10.5114/reum.2019.87615
8. Aktaş, O., Dönmez, A. A., Kapucu, S., & Çinar, M. (2025). The effect of warm saltwater and warm water baths on pain, fatigue, sleep quality, and functional capacity in patients with rheumatoid arthritis: a randomized controlled study✰. *BMC Complementary Medicine and Therapies*, *25*(1). https://doi.org/10.1186/s12906-025-05050-2
9. Albayrak Gezer, İ., Balkarli, A., Can, B., Bağçaci, S., Küçükşen, S., & Küçük, A. (2017). Pain, depression levels, fatigue, sleep quality, and quality of life in elderly patients with rheumatoid arthritis. *Turkish Journal of Medical Sciences*, *47*(3), 847–853. https://doi.org/10.3906/sag-1603-147
10. Alburquerque-García, A., Rodrigues-De-Souza, D. P., Fernández-De-Las-Peñas, C., & Alburquerque-Sendín, F. (2015). Association between muscle trigger points, ongoing pain, function, and sleep quality in elderly women with bilateral painful knee osteoarthritis. *Journal of Manipulative and Physiological Therapeutics*, *38*(4), 262–268. https://doi.org/10.1016/j.jmpt.2014.10.018
11. Allen, K. D., Renner, J. B., Devellis, B., Helmick, C. G., & Jordan, J. M. (2008). Osteoarthritis and sleep: the Johnston County Osteoarthritis Project. *The Journal of rheumatology*, *35*(6), 1102–1107.
12. Altaş, E. U., & Demirdal, Ü. S. (2020). The effect of physical therapy and rehabilitation modalities on sleep quality in patients with primary knee osteoarthritis: A single-blind, prospective, randomized-controlled study. *Turkish Journal of Physical Medicine and Rehabilitation*, *66*(1), 73–83. https://doi.org/10.5606/tftrd.2020.3089
13. Altındağ, Ö., Akaltun, M. S., & Gür, A. (2021). Daytime Sleepiness and Its Relationship with Disease Activity and Emotional State in Patients with Ankylosing Spondylitis. *Journal of Turkish Sleep Medicine*, *8*(3), 192–196. https://doi.org/10.4274/jtsm.galenos.2021.22931
14. Anjum, S., Asim Arif, M., Khalid, A., Iqbal, A., Sana Ashraf, H., & Sohail, M. (2022). Impact of disease activity level on sleep quality in patients with rheumatoid arthritis: A cross-sectional study. In *Rawal Medical Journal* (Vol. 47, Issue 1).
15. Atçi, A. G. (2022). Investigation of the Effects of Physical Therapy and Spa Treatment on Pain and Sleep Quality in Patients with Osteoarthritis Investigarea Efectelor Kinetoterapiei și ale Tratamentului Balnear asupra Durerii și Calității Somnului la Pacienții cu Osteoartrită. In *Revista Română de Kinetoterapie* (Vol. 28).
16. Austad, C., Kvien, T. K., Olsen, I. C., & Uhlig, T. (2017). Sleep disturbance in patients with rheumatoid arthritis is related to fatigue, disease activity, and other patient-reported outcomes. *Scandinavian Journal of Rheumatology*, *46*(2), 95–103. https://doi.org/10.3109/03009742.2016.1168482
17. Aydemir, B., Muhammad, L. N., Song, J., Reid, K. J., Grimaldi, D., Isaacs, A., Carns, M., Dennis-Aren, K., Dunlop, D. D., Chang, R. W., Zee, P. C., & Lee, Y. C. (2024). Characterization of sleep disturbance in established rheumatoid arthritis patients: exploring the relationship with central nervous system pain regulation. *BMC Rheumatology*, *8*(1). https://doi.org/10.1186/s41927-024-00405-7
18. Aydemir, B., Schieir, O., Valois, M. F., Muhammad, L. N., Song, J., Dunlop, D., Chang, R. W., Bartlett, S. J., Bessette, L., Boire, G., Hazlewood, G., Hitchon, C., Pope, J., Thorne, C., Tin, D., Bykerk, V. P., & Lee, Y. C. (2025). Association Between Sleep Disturbance and Subsequent Pain Interference in Patients With Early Rheumatoid Arthritis. *Arthritis Care and Research*, *77*(9), 1078–1084. https://doi.org/10.1002/acr.25568
19. Aydin, E., Bayraktar, K., Turan, Y., Omurlu, I., Tastaban, E., & Sendur, O. F. (2015). Qualidade do sono em pacientes com espondilite anquilosante. *Revista Brasileira de Reumatologia*, *55*(4), 340–345. https://doi.org/10.1016/j.rbr.2014.12.007
20. Aykurt Karlıbel, I., & Kasapoğlu Aksoy, M. (2023). The relationship between central sensitization and disease activity, quality of life, and sleep quality among patients with axial spondyloarthritis. *Irish Journal of Medical Science*, *192*(1), 481–489. https://doi.org/10.1007/s11845-022-02957-4
21. Azizli, A., Sargın, G., & Senturk, T. (2023). The effect of disease-modifying antirheumatic drugs on sleep and quality of life in older patients with rheumatoid arthritis. *Journal of Rheumatic Diseases*, *30*(2), 99–105. https://doi.org/10.4078/jrd.22.0053
22. Azzam, A. I. (2024). The Impact of Sleep Problems on Rheumatoid Arthritis Disease Activity. *Sleep Medicine Research*, *15*(2), 106–112. https://doi.org/10.17241/smr.2023.02075
23. Babul, N., Noveck, R., Chipman, H., Roth, S. H., Gana, T., & Albert, K. (2004). Efficacy and safety of extended-release, once-daily tramadol in chronic pain: A randomized 12-week clinical trial in osteoarthritis of the knee. *Journal of Pain and Symptom Management*, *28*(1), 59–71. https://doi.org/10.1016/j.jpainsymman.2003.11.006
24. Bagnato, G. L., Fiorenza, A., Cordova, F., Roberts, W. N., Moore, C., Jr, Greco, D., Monaco, C., Muscatello, M. R., Bruno, A., Zoccali, R., & Bagnato, G. (2016). Clinical, autoimmune, and psychiatric parameters correlate with sleep disturbance in patients with systemic sclerosis and rheumatoid arthritis. *Clinical and experimental rheumatology, 34 Suppl 100*(5), 49–55*.*
25. Bakir, E., Baglama, S. S., & Gursoy, S. (2018). The effects of reflexology on pain and sleep deprivation in patients with rheumatoid arthritis: A randomized controlled trial. *Complementary Therapies in Clinical Practice*, *31*, 315–319. https://doi.org/10.1016/j.ctcp.2018.02.017
26. Baldelli, L., Addimanda, O., Burattini, M., Chiaro, G., Brusi, V., Pignotti, E., Meliconi, R., & Provini, F. (2019). Nightmare disorder and REM sleep behavior disorder in inflammatory arthritis: Possibility beyond neurodegeneration. *Brain and Behavior*, *9*(3). https://doi.org/10.1002/brb3.1230
27. Bandinelli, F., Delle Sedie, A., Mallia, I., Mauro, I., Pecani, N., Carli, L., Esti, L., Di Carlo, M., Carotti, M., & Salaffi, F. (2025). Causes of Sleep Disturbance in Early ASAS Spondyloarthritis: A Retrospective Long-Term Experience. *Journal of Personalized Medicine*, *15*(1). https://doi.org/10.3390/jpm15010031
28. Bao, C., Huang, F., Khan, M. A., Fei, K., Wu, Z., Han, C., & Hsia, E. C. (2014). Safety and efficacy of golimumab in Chinese patients with active ankylosing spondylitis: 1-year results of a multicentre, randomized, double-blind, placebo-controlled phase III trial. *Rheumatology (United Kingdom)*, *53*(9), 1654–1663. https://doi.org/10.1093/rheumatology/keu132
29. Bartlett, S. J., Bingham, C. O., van Vollenhoven, R., Murray, C., Gruben, D., Gold, D. A., & Cella, D. (2022). The impact of tofacitinib on fatigue, sleep, and health-related quality of life in patients with rheumatoid arthritis: a post hoc analysis of data from Phase 3 trials. *Arthritis Research and Therapy*, *24*(1). https://doi.org/10.1186/s13075-022-02724-x
30. Bedaiwi, M. K., Alkhalaf, A., Almaghlouth, I. A., Alqurtas, E. M., Almutairi, S. ‎, Almutairi, F., Alqahtani, T., Alasbali, M., & Omair, M. A. (2021). Correlation of Sleep Quality in Spondyloarthritis Patients and its Association with Disease Activitiy Parameters. *Pakistan Journal of Medical and Health Sciences*, *15*(6), 2089–2093. https://doi.org/10.53350/pjmhs211562089
31. Belt, N. K., Kronholm, E., & Kauppi, M. J. (2009). Sleep problems in fibromyalgia and rheumatoid arthritis compared with the general population. *Clinical and experimental rheumatology, 27*(1), 35–41.
32. Bertram, W., Penfold, C., Glynn, J., Johnson, E., Burston, A., Rayment, D., Howells, N., White, S., Wylde, V., Gooberman-Hill, R., Blom, A., & Whale, K. (2024). REST: a preoperative tailored sleep intervention for patients undergoing total knee replacement - feasibility study for a randomised controlled trial. *BMJ Open*, *14*(3). https://doi.org/10.1136/bmjopen-2023-078785
33. Bilge, N. S. Y., Bilge, U., Kilic, S., & Bilgin, M. (2017). The assessment of sleep quality in rheumatoid arthritis patients. *BIOMEDICAL RESEARCH-INDIA*, *28*.
34. Bilge, N. S. Y., Guler, S., & Bilge, U. (2016). Comparison of the frequency of sleep disorders among patients with rheumatoid arthritis and type 2 diabetic patients. *Konuralp Tip Dergisi*, *8*(2), 100-103.
35. Bjurström, M. F., Irwin, M. R., Bodelsson, M., Smith, M. T., & Mattsson-Carlgren, N. (2021). Preoperative sleep quality and adverse pain outcomes after total hip arthroplasty. *European Journal of Pain (United Kingdom)*, *25*(7), 1482–1492. https://doi.org/10.1002/ejp.1761
36. Bjurström, M. F., Olmstead, R., & Irwin, M. R. (2017). Reciprocal Relationship between Sleep Macrostructure and Evening and Morning Cellular Inflammation in Rheumatoid Arthritis. *Psychosomatic Medicine*, *79*(1), 24–33. https://doi.org/10.1097/PSY.0000000000000363
37. Bordvik, D. H., Steen Pettersen, P., Gløersen, M., Mulrooney, E., Neogi, T., Kjeken, I., & Haugen, I. K. (2025). The associations between sleep problems and pain outcomes in people with hand osteoarthritis – Data from the Nor-hand study. *Osteoarthritis and Cartilage Open*, *7*(1). https://doi.org/10.1016/j.ocarto.2025.100579
38. Borman, P., & Çeliker, R. (1999). A comparative analysis of quality of life in rheumatoid arthritis and fibromyalgia. *Journal of Musculoskeletal Pain*, *7*(4), 5–14. https://doi.org/10.1300/J094v07n04_02
39. Boye Larsen, D., Laursen, M., Simonsen, O., Arendt-Nielsen, L., & Petersen, K. K. (2021). The association between sleep quality, preoperative risk factors for chronic postoperative pain and postoperative pain intensity 12 months after knee and hip arthroplasty. *British Journal of Pain*, *15*(4), 486–496. https://doi.org/10.1177/20494637211005803
40. Brahem, M., Chebil, A., Abid, H., Jomaa, O., Sarraj, R., Jebali, B., Abdellatif, S., ben Salem, A., Hachfi, H., Mighri, K., & Younes, M. (2024). Sleep disorders in rheumatoid arthritis patients. *Egyptian Rheumatologist*, *46*(3), 107–111. https://doi.org/10.1016/j.ejr.2024.03.001
41. Buchanan, D. T., Vitiello, M. V., & Bennett, K. (2017). Feasibility and Efficacy of a Shared Yoga Intervention for Sleep Disturbance in Older Adults With Osteoarthritis*. Journal of gerontological nursing,* 1–10. Advance online publication. https://doi.org/10.3928/00989134-20170405-01
42. Cai, Y., Chen, J., Dou, J., Zhou, N., Shao, H., Shen, X., Hong, M., Chen, J., Fan, X., Hu, Q., & Lu, C. (2025). Relationships between emotional state, sleep disturbance and health-related quality of life in patients with axial spondyloarthritis. *Clinical Rheumatology*, *44*(1), 267–276. https://doi.org/10.1007/s10067-024-07246-2
43. Çakirbay, H., Bilici, M., Kavakçi, Ö., Çebi, A., Güler, M., & Tan, Ü. (2004). Sleep quality and immune functions in rheumatoid arthritis patients with and without major depression. *International Journal of Neuroscience*, *114*(2), 245–256. https://doi.org/10.1080/00207450490269471
44. Campbell, C. M., Buenaver, L. F., Finan, P., Bounds, S. C., Redding, M., McCauley, L., Robinson, M., Edwards, R. R., & Smith, M. T. (2015). Sleep, Pain Catastrophizing, and Central Sensitization in Knee Osteoarthritis Patients with and Without Insomnia. *Arthritis Care and Research*, *67*(10), 1387–1396. https://doi.org/10.1002/acr.22609
45. Cano-García, L., Mena-Vázquez, N., Manrique Arija, S., Hernández-Sánchez, M. D., Segura-Ruiz, R., Domínguez-Quesada, C., & Fernández-Nebro, A. (2021). Psychological factors associated with sleep disorders in patients with axial spondyloarthritis or psoriatic arthritis: A multicenter cross-sectional observational study. *Journal of Clinical Nursing*, *30*(1–2), 266–275. https://doi.org/10.1111/jocn.15546
46. Chalupczak, N. V., Aydemir, B., Isaacs, A., Muhammad, L. N., Song, J., Reid, K. J., Grimaldi, D., Carns, M., Dennis-Aren, K., Dunlop, D. D., Wallace, B. I., Zee, P. C., & Lee, Y. C. (2025). Sleep Matters: Exploring the Link Between Sleep Disturbances and Fatigue in Rheumatoid Arthritis. *Arthritis Care and Research*. https://doi.org/10.1002/acr.25579
47. Chau, S. Y. L., Leung, M. H. A., & Tang, W. K. (2023). The association of disease activity with depression and sleep quality in patients with rheumatoid arthritis in Hong Kong. *International Journal of Rheumatic Diseases*, *26*(12), 2543–2550. https://doi.org/10.1111/1756-185X.14957
48. Chen, A. F., Orozco, F. R., Austin, L. S., Post, Z. D., Deirmengian, C. A., & Ong, A. C. (2016). Prospective Evaluation of Sleep Disturbances After Total Knee Arthroplasty. *Journal of Arthroplasty*, *31*(1), 330–332. https://doi.org/10.1016/j.arth.2015.07.044
49. Chen, C. J., McHugh, G., Campbell, M., & Luker, K. (2015). Subjective and Objective Sleep Quality in Individuals with Osteoarthritis in Taiwan. *Musculoskeletal Care*, *13*(3), 148–159. https://doi.org/10.1002/msc.1094
50. Cheung, C., Wyman, J. F., Resnick, B., & Savik, K. (2014). Yoga for managing knee osteoarthritis in older women: A pilot randomized controlled trial. *BMC Complementary and Alternative Medicine*, *14*. https://doi.org/10.1186/1472-6882-14-160
51. Cho, Y. W., Na, G. Y., Lim, J. G., Kim, S. H., Kim, H. S., Earley, C. J., & Allen, R. P. (2013). Prevalence and clinical characteristics of restless legs syndrome in diabetic peripheral neuropathy: Comparison with chronic osteoarthritis. *Sleep Medicine*, *14*(12), 1387–1392. https://doi.org/10.1016/j.sleep.2013.09.013
52. Clarke, L. L., Wilson, S., & Kirwan, J. R. (2013). Using actigraphy to measure sleep patterns in rheumatoid arthritis: A pilot study in patients taking night-time prednisone. *Musculoskeletal Care*, *11*(3), 179–185. https://doi.org/10.1002/msc.1052
53. Coksevim, N. H., Durmus, D., & Kuru, O. (2018). Effects of global postural reeducation exercise and anti-TNF treatments on disease activity, function, fatigue, mobility, sleep quality and depression in patients with active Ankylosing spondylitis: A prospective follow-up study. In *Journal of Back and Musculoskeletal Rehabilitation* (Vol. 31, Issue 6, pp. 1005–1012). IOS Press. https://doi.org/10.3233/BMR-170901
54. Cremeans-Smith, J. K., Millington, K., Sledjeski, E., Greene, K., & Delahanty, D. L. (2006). Sleep disruptions mediate the relationship between early postoperative pain and later functioning following total knee replacement surgery. *Journal of Behavioral Medicine*, *29*(2), 215–222. https://doi.org/10.1007/s10865-005-9045-0
55. Crosby, L. J. (1988). EEG sleep variables of rheumatoid arthritis patients. *Arthritis & Rheumatism: Official Journal of the American College of Rheumatology*, *1*(4), 198-204. https://doi.org/10.1002/anr.1790010404
56. Crosby L. J. (1991). Factors which contribute to fatigue associated with rheumatoid arthritis. *Journal of advanced nursing*, *16*(8), 974–981. https://doi.org/10.1111/j.1365-2648.1991.tb01803.x
57. Da Costa, D., Zummer, M., & Fitzcharles, M.-A. (2009). Determinants of sleep problems in patients with spondyloarthropathy. *Musculoskeletal Care*, *7*(3), 143–161. https://doi.org/10.1002/msc
58. de Leeuw, A. W., van Wissen, M. A. T., Vlieland, T. P. M. V., van Tubergen, A. M., Gademan, M. G. J., Berger, M. A. M., & van Weely, S. F. E. (2025). Personalized physical activity recommendations for people with axial spondyloarthritis using wearable activity tracker data: an exploratory study. *Rheumatology International*, *45*(3). https://doi.org/10.1007/s00296-024-05755-6
59. Demir, S., Kucuk, A., Altas, M., & Cure, E. (2021). Restless Leg Syndrome and Sleep Disorders in Patients with Rheumatoid Arthritis and Its Relation with Anemia Parameters. *Acta Medica*, *64*(3), 137–144. https://doi.org/10.14712/18059694.2021.24
60. Demirci, S., Demirci, K., Doğru, A., İnal, E. E., Koyuncuoğlu, H. R., & Şahin, M. (2016). Restless legs syndrome is associated with poor sleep quality and quality of life in patients with ankylosing spondylitis: a questionnaire-based study. *Acta Neurologica Belgica*, *116*(3), 329–336. https://doi.org/10.1007/s13760-015-0564-3
61. Deniz, O., Cavusoglu, C., Satis, H., Salman, R. B., Varan, O., Atas, N., Coteli, S., Dogrul, R. T., Babaoglu, H., Oncul, A., Varan, H. D., Kizilarslanoglu, M. C., Tufan, A., & Goker, B. (2023). Sleep quality and its associations with disease activity and quality of life in older patients with rheumatoid arthritis. *European Geriatric Medicine*, *14*(2), 317–324. https://doi.org/10.1007/s41999-022-00739-w
62. Deodhar, A., Braun, J., Inman, R. D., Mack, M., Parasuraman, S., Buchanan, J., Hsu, B., Gathany, T., & Van Der Heijde, D. (2010). Golimumab reduces sleep disturbance in patients with active ankylosing spondylitis: Results from a randomized, placebo-controlled trial. *Arthritis Care and Research*, *62*(9), 1266–1271. https://doi.org/10.1002/acr.20233
63. Deodhar, A., Mease, P., Marzo-Ortega, H., Hunter, T., Sandoval, D., Kronbergs, A., Lauzon, S., Leung, A., & Navarro-Compán, V. (2021). Ixekizumab improves sleep and work productivity in patients with non-radiographic axial spondyloarthritis: results from the COAST-X trial at 52 weeks. *BMC Rheumatology*, *5*(1). https://doi.org/10.1186/s41927-021-00218-y
64. Detert, J., Dziurla, R., Hoff, P., Gaber, T., Klaus, P., Bastian, H., Braun, T., Schellmann, S., Penzel, T., Fietze, I., Loeschmann, P.-A., Jaehnig, P., Straub, R. H., Burmester, G. R., & Buttgereit, F. (2016). Effects of treatment with etanercept versus methotrexate on sleep quality, fatigue and selected immune parameters in patients with active rheumatoid arthritis. In *Clinical and Experimental Rheumatology* (Vol. 34). http://register.germanctr.de,
65. Döner, Ş. İ., Gerçek, H., Sert, Ö. A., Aytar, A., & Aytar, A. (2024). The effects of aromatherapy massage in menopausal women with knee osteoarthritis: A randomized controlled study. *Explore*, *20*(6). https://doi.org/10.1016/j.explore.2024.05.012
66. Dougados, M., Tsai, W. C., Saaibi, D. L., Bonin, R., Bukowski, J., Pedersen, R., Vlahos, B., & Kotak, S. (2015). Evaluation of health outcomes with etanercept treatment in patients with early nonradiographic axial spondyloarthritis. *Journal of Rheumatology*, *42*(10), 1835–1841. https://doi.org/10.3899/jrheum.141313
67. Drewes, A. M., Bjerregård, K., Taagholt, S. J., Svendsen, L., & Nielsen, K. D. (1998). Zopiclone as night medication in rheumatoid arthritis. *Scandinavian journal of rheumatology*, *27*(3), 180–187. https://doi.org/10.1080/030097498440787
68. Drewes, A. M., Svendsen, L., Taagholt, S. J., Bjerregård, K., Nielsen, K. D., & Hansen, B. (1998). Sleep in rheumatoid arthritis: a comparison with healthy subjects and studies of sleep/wake interactions. *British journal of rheumatology*, *37*(1), 71–81. https://doi.org/10.1093/rheumatology/37.1.71
69. Dubreuil, M., Navarro-Compán, V., Boonen, A., Gaffney, K., Gensler, L. S., De La Loge, C., Vaux, T., Fleurinck, C., Massow, U., Taieb, V., Mørup, M. F., Deodhar, A., & Rudwaleit, M. (2024). Improved physical functioning, sleep, work productivity and overall health-related quality of life with bimekizumab in patients with axial spondyloarthritis: Results from two phase 3 studies. *RMD Open*, *10*(2). https://doi.org/10.1136/rmdopen-2024-004202
70. Durcan, L., Wilson, F., & Cunnane, G. (2014). The effect of exercise on sleep and fatigue in rheumatoid arthritis: A randomized controlled study. *Journal of Rheumatology*, *41*(10), 1966–1973. https://doi.org/10.3899/jrheum.131282
71. Durmus, D., Sarisoy, G., Alayli, G., Kesmen, H., Çetin, E., Bilgici, A., Kuru, O., & Ünal, M. (2015). Psychiatric symptoms in ankylosing spondylitis: their relationship with disease activity, functional capacity, pain and fatigue. *Comprehensive Psychiatry*, *62*, 170–177. https://doi.org/10.1016/j.comppsych.2015.07.016
72. Er, M. S., Altinel, E. C., Altinel, L., Erten, R. A., & Eroǧlu, M. (2014). An assessment of sleep quality in patients undergoing total knee arthroplasty before and after surgery. *Acta Orthopaedica et Traumatologica Turcica*, *48*(1), 50–54. https://doi.org/10.3944/AOTT.2014.3163
73. Farzana, M., & Kumar, M. (2020). Effect of Jacobson’s Progressive Relaxation Technique Over Sleep Disturbances and Quality of Life in Chronic Rheumatoid Arthritis. *Indian Journal of Public Health Research & Development*, *11*(2), 108. https://doi.org/10.37506/v11/i2/2020/ijphrd/194763
74. Fatah, R. N., & Abdulrahman, B. (2020). A sleep disturbance after total knee arthroplasty. *Journal of Family Medicine and Primary Care*, *9*(1), 119. https://doi.org/10.4103/jfmpc.jfmpc_595_19
75. Fawzy, R. M., Abdel-Monem, S. M., El-Brashi, A. W. S., & Mohamed, A. A. (2022). A comparative study between rheumatoid arthritis and osteoarthritis regarding association of insomnia with disease status. *Egyptian Rheumatology and Rehabilitation*, *49*(1). https://doi.org/10.1186/s43166-021-00108-8
76. Feehan, L., Xie, H., Lu, N., & Li, L. C. (2024a). Twenty-four hour physical activity, sedentary behaviour and sleep profiles in adults living with rheumatoid arthritis: a cross-sectional latent class analysis. *Journal of Activity, Sedentary and Sleep Behaviors*, *3*(1). https://doi.org/10.1186/s44167-024-00049-5
77. Fertelli, T. K., & Tuncay, F. O. (2019). Fatigue in individuals with knee osteoarthritis: Its relationship with sleep quality, pain and depression. *Pakistan journal of medical sciences*, *35*(4), 1040. https://doi.org/10.12669/pjms.35.4.383
78. Fielden, J. M., Gander, P. H., Horne, J. G., Lewer, B. M. F., Green, R. M., & Devane, P. A. (2003). An assessment of sleep disturbance in patients before and after total hip arthroplasty. *Journal of Arthroplasty*, *18*(3), 371–376. https://doi.org/10.1054/arth.2003.50056
79. Florete, O. G., Xiang, J., Vorsanger, G. J., & Janssen Scientifi, O.-M. (2008). Effects of extended-release tramadol on pain-related sleep parameters in patients with osteoarthritis. *Expert Opin. Pharmacother*, *9*(11), 1817–1827. https://doi.org/10.1517/14656560802217606
80. Fongen, C., Dagfinrud, H., Bilberg, A., & Sveaas, S. H. (2024). Reduced sleep quality is highly prevalent and associated with physical function and cardiorespiratory fitness in patients with axial spondyloarthritis: a cross-sectional study. *Scandinavian Journal of Rheumatology*, *53*(2), 130–139. https://doi.org/10.1080/03009742.2023.2281069
81. Fouda, N., & Dayem, A. A. (2014). Obstructive sleep apnea in patients with rheumatoid arthritis: Correlation with disease activity and pulmonary function tests. *Egyptian Rheumatologist*, *36*(4), 165–171. https://doi.org/10.1016/j.ejr.2014.04.002
82. Fragiadaki, K., Tektonidou, M. G., Konsta, M., Chrousos, G. P., & Sfikakis, P. P. (2012). Sleep disturbances and interleukin 6 receptor inhibition in rheumatoid arthritis. *Journal of Rheumatology*, *39*(1), 60–62. https://doi.org/10.3899/jrheum.110617
83. Frede, N., Rieger, E., Lorenzetti, R., Venhoff, A. C., Kanne, A. M., Finzel, S., Jandova, I., Glaser, C., Thiel, J., Voll, R. E., & Venhoff, N. (2023). Sleep behaviour differs in women and men with psoriatic arthritis and axial spondyloarthritis with impact on quality of life and depressive symptoms. *RMD Open*, *9*(2). https://doi.org/10.1136/rmdopen-2022-002912
84. Fu, K., Makovey, J., Metcalf, B., Bennell, K. L., Zhang, Y., Asher, R., Robbins, S. R., Deveza, L. A., Cistulli, P. A., & Hunter, D. J. (2019). Sleep quality and fatigue are associated with pain exacerbations of hip osteoarthritis: An internet-based case-crossover study. *Journal of Rheumatology*, *46*(11), 1524–1530. https://doi.org/10.3899/jrheum.181406
85. Gamal, R. M., Mohamed, M. E., Hammam, N., El Fetoh, N. A., Rashed, A. M., & Furst, D. E. (2020). Preliminary study of the association of serum irisin levels with poor sleep quality in rheumatoid arthritis patients. *Sleep Medicine*, *67*, 71–76. https://doi.org/10.1016/j.sleep.2019.10.021
86. Genty, M., Combe, B., Kostine, M., Ardouin, E., Morel, J., Lukas, C., Genty, M., Combe, B., Kostine, M., Ardouin, E., Morel, J., & Lukas, C. (2017). Improvement of fatigue in patients with rheumatoid arthritis treated with biologics: relationship with sleep disorders, depression and clinical efficacy. A prospective, multicentre study Improvement of fatigue in RA patients treated with biologics / M. Genty et al. In *Clinical and Experimental Rheumatology* (Vol. 35).
87. George, R. J., Kumar, R., Achenbach, S. J., Lovering, E., Lennon, R. J., Davis, J. M., Carvalho, D. Z., Crowson, C. S., & Myasoedova, E. (2025). Sleep disorders in rheumatoid arthritis: Incidence, risk factors and association with dementia. *Seminars in Arthritis and Rheumatism*, *73*. https://doi.org/10.1016/j.semarthrit.2025.152722
88. Gezer, O., Batmaz, İ., Sariyildiz, M. A., Sula, B., Ucmak, D., Bozkurt, M., & Nas, K. (2017). Sleep quality in patients with psoriatic arthritis. *International Journal of Rheumatic Diseases*, *20*(9), 1212–1218. https://doi.org/10.1111/1756-185X.12505
89. Ghani Sayed, E. A., Samy, S., Abdi, F. A., & Abou-Raya, A. (2025). Occurrence and assessment of chronic pain fatigue and sleep disturbance in axial spondyloarthritis patients. *Alexandria Journal of Medicine*, *61*(1), 1–7. https://doi.org/10.1080/20905068.2025.2460313
90. Gjevre, J. A., Taylor-Gjevre, R. M., Nair, B. V., & Lim, H. J. (2012). Do Sleepy Rheumatoid Arthritis Patients Have A Sleep Disorder? *Musculoskeletal Care*, *10*(4), 187–195. https://doi.org/10.1002/msc.1016
91. Goes, A. C. J., Reis, L. A. B., Silva, M. B. G., Kahlow, B. S., & Skare, T. L. (2017). Rheumatoid arthritis and sleep quality. *Revista Brasileira de Reumatologia (English Edition)*, *57*(4), 294–298. https://doi.org/10.1016/j.rbre.2016.07.011
92. Gohir, S. A., Greenhaff, P., Abhishek, A., & Valdes, A. M. (2019). Evaluating the efficacy of Internet-Based Exercise programme Aimed at Treating knee Osteoarthritis (iBEAT-OA) in the community: A study protocol for a randomised controlled trial. *BMJ Open*, *9*(10). https://doi.org/10.1136/bmjopen-2019-030564
93. Goodchild, C. E., Treharne, G. J., Booth, D. A., & Bowman, S. J. (2010). Daytime patterning of fatigue and its associations with the previous night’s discomfort and poor sleep among women with primary Sjögren’s syndrome or rheumatoid arthritis. *Musculoskeletal Care*, *8*(2), 107–117. https://doi.org/10.1002/msc.174
94. Gouda, W., Mokhtar, M., Elazab, S. A., Alreefi, R., Alomar, T., Kushk, F., Alahmadi, R., Khalil, M., & Kamal, M. (2023). Sleep disorders in patients with rheumatoid arthritis: association with quality of life, fatigue, depression levels, functional disability, disease duration, and activity: a multicentre cross-sectional study. *Journal of International Medical Research*, *51*(10). https://doi.org/10.1177/03000605231204477
95. Grabovac, I., Haider, S., Berner, C., Lamprecht, T., Fenzl, K. H., Erlacher, L., Quittan, M., & Dorner, T. E. (2018). Sleep quality in patients with rheumatoid arthritis and associations with pain, disability, disease duration, and activity. *Journal of Clinical Medicine*, *7*(10). https://doi.org/10.3390/JCM7100336
96. Guaracha-Basáñez, G. A., Contreras-Yáñez, I., Bautista-Torres2, L. E., Ortiz-Haro, A. B., Pascual-Ramos, V., Guaracha-Basáñez, G. A., Contreras-Yáñez, I., Bautista-Torres, L. E., Ortiz-Haro, A. B., & Pascual-Ramos, V. (2022). Sleep quality and predictors of optimal sleep in patients with rheumatoid arthritis: data from a recent-onset cohort Sleep quality in RA patients / G.A. Guaracha-Basáñez et al. In *Clinical and Experimental Rheumatology* (Vol. 41).
97. Günaydin, R., Göksel Karatepe, A., Ceşmeli, N., & Kaya, T. (2009). Fatigue in patients with ankylosing spondylitis: relationships with disease-specific variables, depression, and sleep disturbance. *Clinical Rheumatology*, *28*(9), 1045–1051. https://doi.org/10.1007/s10067-009-1204-1
98. Guo, G., Fu, T., Yin, R., Zhang, L., Zhang, Q., Xia, Y., Li, L., & Gu, Z. (2016). Sleep quality in Chinese patients with rheumatoid arthritis: Contributing factors and effects on health-related quality of life. *Health and Quality of Life Outcomes*, *14*(1). https://doi.org/10.1186/s12955-016-0550-3
99. Hamdi, W., Souissi, M. A., Lassoued Ferjani, H., Maatallah, K., Cherif, I., & Kaffel, D. (2021). Sleep disturbances in chronic rheumatic diseases: Is disease activity the major determinant factor?. *La Tunisie medicale*, *99*(8), 890–897.
100. Hammam, N., Gamal, R. M., Rashed, A. M., Elfetoh, N. A., Mosad, E., & Khedr, E. M. (2020). Fatigue in Rheumatoid Arthritis Patients: Association With Sleep Quality, Mood Status, and Disease Activity. *Reumatologia Clinica*, *16*(5), 339–344. https://doi.org/10.1016/j.reuma.2018.07.010
101. Hawker, G. A., French, M. R., Waugh, E. J., Gignac, M. A. M., Cheung, C., & Murray, B. J. (2010). The multidimensionality of sleep quality and its relationship to fatigue in older adults with painful osteoarthritis. *Osteoarthritis and Cartilage*, *18*(11), 1365–1371. https://doi.org/10.1016/j.joca.2010.08.002
102. Heffner, K. L., France, C. R., Ashrafioun, L., Quiñones, M., Walsh, P., Maloney, M. D., Giordano, B. D., & Pigeon, W. R. (2018). Clinical Pain-related Outcomes and Inflammatory Cytokine Response to Pain Following Insomnia Improvement in Adults with Knee Osteoarthritis. *Clinical Journal of Pain*, *34*(12), 1133–1140. https://doi.org/10.1097/AJP.0000000000000644
103. Hirsch, M., Carlander, B., Vergé, M., Tafti, M., Anaya, J. M., Billiard, M., & Sany, J. (1994). Objective and subjective sleep disturbances in patients with rheumatoid arthritis. A reappraisal. *Arthritis and rheumatism*, *37*(1), 41–49. https://doi.org/10.1002/art.1780370107
104. Hörnberg, K., Pomeroy, J., Sandberg, C., Ångström, L., Södergren, A., & Sundström, B. (2021). Isotemporal Substitution of Time Between Sleep and Physical Activity: Associations With Cardiovascular Risk Factors in Early Rheumatoid Arthritis. *ACR Open Rheumatology*, *3*(3), 138–146. https://doi.org/10.1002/acr2.11225
105. Hu, F., Song, K., Hu, W., Zhang, Z., Liu, C., Wang, Q., Ji, Q., & Zhang, X. (2020). Improvement of Sleep Quality in Patients With Ankylosing Spondylitis Kyphosis After Corrective Surgery. *Spine*, *45*(23), E1596–E1603. https://doi.org/10.1097/BRS.0000000000003676
106. Hughes, M., Chalk, A., Sharma, P., Dahiya, S., & Galloway, J. (n.d.). *A cross-sectional study of sleep and depression in a rheumatoid arthritis population*. https://doi.org/10.1007/s10067-020-05414-8/Published
107. Hultgren, S., Broman, J. E., Gudbjörnsson, B., Hetta, J., & Lindqvist, U. (2000). Sleep disturbances in outpatients with ankylosing spondylitisa questionnaire study with gender implications. *Scandinavian journal of rheumatology*, *29*(6), 365–369. https://doi.org/10.1080/030097400447561
108. İn, E., Turgut, T., Gülkesen, A., & Gündoğdu, B. (2015). Obstructive sleep apnea syndrome and sleep efficiency in patients with ankylosing spondylitis. *Archives of Rheumatology*, *30*(4), 326–333. https://doi.org/10.5606/ArchRheumatol.2015.5698
109. In, E., Turgut, T., Gülkesen, A., Yolbaş, S., Akgöl, G., & Koca, S. S. (2016). Sleep Quality Is Related to Disease Activity in Patients with Ankylosing Spondylitis: A Polysomnographic Study. *Journal of Clinical Rheumatology*, *22*(5), 248–252. https://doi.org/10.1097/RHU.0000000000000355
110. Jeong, H.-J., Lee, T. H., Lee, J. M., Choi, G., Son, C.-N., Kim, J.-M., Cho, Y. W., & Kim, S.-H. (2014). Sleep Disturbances in Korean Patients with Ankylosing Spondylitis are Associated with Increased Disease Activity. *Journal of Rheumatic Diseases*, *21*(5), 241. https://doi.org/10.4078/jrd.2014.21.5.241
111. Jiang, Y., Yang, M., Lv, Q., Qi, J., Lin, Z., Liao, Z., Zhang, Y., Wu, H., Song, H., Zhan, F., Liu, S., Gao, G., Hu, S., Li, Y., Shen, L., Huang, A., Wei, Q., Cao, S., & Gu, J. (2018). Prevalence of psychological disorders, sleep disturbance and stressful life events and their relationships with disease parameters in Chinese patients with ankylosing spondylitis. *Clinical Rheumatology*, *37*(2), 407–414. https://doi.org/10.1007/s10067-017-3907-z
112. Jiang, Y., Yang, M., Wu, H., Song, H., Zhan, F., Liu, S., Gao, G., Liu, Z., Hu, Z., He, P., Zhang, S., Hu, Z., Lin, Z., Zhang, Y., Li, Y., Shen, L., Huang, A., Liao, Z., Cao, S., … Gu, J. (2015). The relationship between disease activity measured by the BASDAI and psychological status, stressful life events, and sleep quality in ankylosing spondylitis. *Clinical Rheumatology*, *34*(3), 503–510. https://doi.org/10.1007/s10067-014-2688-x
113. Juárez-Rojop, I. E., Fresán, A., Genis-Mendoza, A. D., Cerino-Palomino, C., Nolasco-Rosales, G. A., González-Castro, T. B., López-Narváez, M. L., Olan, F., Villar-Soto, M., Tovilla-Zárate, C. A., & Nicolini, H. (2023). Prevalence of Poor Sleep Quality and Associated Factors in Individuals with Rheumatoid Arthritis: A Cross-Sectional Study. *Medicina (Lithuania)*, *59*(9). https://doi.org/10.3390/medicina59091633
114. Kakazu, V. A., Pinto, R. Z., Dokkedal-Silva, V., Fernandes, G. L., Araujo, C. G. A., Pires, G. N., Dias, T. A., Tufik, S., Andersen, M. L., & Morelhão, P. K. (2024). Are Sleep Quality, Daytime Sleepiness, and Depression Associated with Knee Pain? A Cross-Sectional Study in Older Adults. *Sleep Science*, *18*(1), e91–e96. https://doi.org/10.1055/s-0044-1787528
115. Karadaǧ, Ö., Nakas, D., Kalyoncu, U., Akdoǧan, U., Kiraz, S., & Ertenli, I. (2012). Effect of anti-TNF treatment on sleep problems in ankylosing spondylitis. *Rheumatology International*, *32*(7), 1909–1913. https://doi.org/10.1007/s00296-011-1907-x
116. Karatas, G., Bal, A., Yuceege, M., Firat, H., Gurcay, E., Ardic, S., & Cakci, F. A. (2018). Evaluation of sleep quality in patients with ankylosing spondylitis and efficacy of anti-TNF-α therapy on sleep problems: A polisomnographic study. *International Journal of Rheumatic Diseases*, *21*(6), 1263–1269. https://doi.org/10.1111/1756-185X.13102
117. Karatas, G., Bal, A., Yuceege, M., Yalcin, E., Firat, H., Dulgeroglu, D., Karataş, F., Sahin, S., Cakci, A., & Ardic, S. (2017). The evaluation of sleep quality and response to anti-tumor necrosis factor α therapy in rheumatoid arthritis patients. *Clinical Rheumatology*, *36*(1), 45–50. https://doi.org/10.1007/s10067-016-3387-6
118. Katz, P., Margaretten, M., Trupin, L., Schmajuk, G., Yazdany, J., & Yelin, E. (2016). Role of Sleep Disturbance, Depression, Obesity, and Physical Inactivity in Fatigue in Rheumatoid Arthritis. *Arthritis Care and Research*, *68*(1), 81–90. https://doi.org/10.1002/acr.22577
119. Katz, P., Pedro, S., & Michaud, K. (2023). Sleep Disorders Among Individuals With Rheumatoid Arthritis. *Arthritis Care and Research*, *75*(6), 1250–1260. https://doi.org/10.1002/acr.24999
120. Kaya, E., Kaplan, C., Çarli, A. B., & Güzelküçük, Ü. (2016). Effects of balneotherapy and physical therapy on sleep quality in patients with osteoarthritis aged 50 to 85 years. *Archives of Rheumatology*, *31*(1), 1–5. https://doi.org/10.5606/ArchRheumatol.2016.4625
121. Kayahan Satış, N., & Satış, H. (2025). Sleep problems in elderly patients with rheumatoid arthritis: Contributing factors and quality of life implications. *Journal of Turkish Society For Rheumatology*, *17*(1), 45–52. https://doi.org/10.4274/raed.galenos.2024.39200
122. Kim, C. S., Jang, S. H., & Cho, Y. Y. (2015). The Effect of Laughter Therapy on Arthralgia, Ankylosis, Depression, and Sleep of Elderly Housebound Women with Osteoarthritis. *Journal of Korean Biological Nursing Science*, *17*(2), 123–131. https://doi.org/10.7586/jkbns.2015.17.2.123
123. Kim, S. H., Kim, H., Kim, S., Paek, S. I., Koh, J. H., Lee, J., Cho, Y. W., & Park, S. H. (2018). Sleep quality independently affects health-related quality of life and cognitive function in Korean female patients with rheumatoid arthritis: A case-control study. *Journal of Korean Medical Science*, *33*(35). https://doi.org/10.3346/jkms.2018.33.e216
124. Kim, C. S., Yoo, K. S., & Hong, S. H. (2014). The effects of foot reflexology on arthralgia, ankylosis, depression, and sleep in community-dwelling elderly women with osteoarthritis. *Journal of Korean Academy of Community Health Nursing*, *25*(3), 207-216.
125. Kivitz, A., Ma, C., Ahdieh, H., & Galer, B. S. (2006). *A 2-Week, Multicenter, Randomized, Double-Blind, Placebo-Controlled, Dose-Ranging, Phase III Trial Comparing the Efficacy of Oxymorphone Extended Release and Placebo in Adults with Pain Associated with Osteoarthritis of the Hip or Knee*.
126. Kılıç, N., & Parlar Kılıç, S. (2023). The effect of progressive muscle relaxation on sleep quality and fatigue in patients with rheumatoid arthritis: A randomized controlled trial. *International Journal of Nursing Practice*, *29*(3). https://doi.org/10.1111/ijn.13015
127. Koken, M., & Guclu, B. (2019). The effects of total knee arthroplasty on sleep quality. *Malaysian Orthopaedic Journal*, *13*(2), 11–14. https://doi.org/10.5704/MOJ.1907.002
128. Kontodimopoulos, N., Stamatopoulou, E., Kletsas, G., & Kandili, A. (2020). Disease activity and sleep quality in rheumatoid arthritis: a deeper look into the relationship. *Expert Review of Pharmacoeconomics and Outcomes Research*, *20*(6), 595–602. https://doi.org/10.1080/14737167.2020.1677156
129. Kosinski, M., Janagap, C. C., Gajria, K., Schein, J., & Freedman, J. (2007). Pain relief and pain-related sleep disturbance with extended-release tramadol in patients with osteoarthritis. *Current Medical Research and Opinion*, *23*(7), 1615–1626. https://doi.org/10.1185/030079907X199493
130. Krajewska-Włodarczyk, M., Owczarczyk-Saczonek, A., & Placek, W. (2018). Sleep disorders in patients with psoriatic arthritis and psoriasis. *Reumatologia*, *56*(5), 301–306. https://doi.org/10.5114/reum.2018.79501
131. Kruthik, G., Ansari, M. S., & Patil, P. (2025). Study of Sleep Problem in Rheumatoid Arthritis and Its Impact on Disease Activity, Pain Perception, and Functional Disability. *Annals of African Medicine*. https://doi.org/10.4103/aam.aam_206_24
132. Kumar, A., Handa, R., Upadhyaya, S., Gupta, S., & Malgutte, D. (2022). Sleep quality in rheumatoid arthritis. *Indian Journal of Rheumatology*, *17*(1), 34–40. https://doi.org/10.4103/injr.injr_151_21
133. Kuralay, C., & Kiyak, E. (2018). Sleep quality and factors affecting patients with knee osteoarthritis. *International Journal of Caring Sciences*, *11*(2), 1141-1146.
134. Kwiatkowska, B., Kłak, A., Raciborski, F., & Maślińska, M. (2019). The prevalence of depression and insomnia symptoms among patients with rheumatoid arthritis and osteoarthritis in Poland: a case control study. *Psychology, Health and Medicine*, *24*(3), 333–343. https://doi.org/10.1080/13548506.2018.1529325
135. Latocha, K. M., Loppenthin, K. B., Ostergaard, M., Jennum, P. J., Hetland, M. L., Rogind, H., Lundbak, T., Midtgaard, J., Christensen, R., & Esbensen, B. A. (2023). The effect of group-based cognitive behavioural therapy for insomnia in patients with rheumatoid arthritis: A randomized controlled trial. *Rheumatology (United Kingdom)*, *62*(3), 1097–1107. https://doi.org/10.1093/rheumatology/keac448
136. Lavie, P., Nahir, M., Lorber, M., & Scharf, Y. (1991). Nonsteroidal antiinflammatory drug therapy in rheumatoid arthritis patients. Lack of association between clinical improvement and effects on sleep. *Arthritis and rheumatism*, *34*(6), 655–659. https://doi.org/10.1002/art.1780340605
137. LeBrun, D. G., Grubel, J., Ong, J., Chiu, Y. F., Blevins, J. L., Haas, S. B., Rodriguez, J. A., Gausden, E. B., Cushner, F. D., Lee, G. C., Gonzalez Della Valle, A., & Chalmers, B. P. (2024). The John N. Insall Award: Does Melatonin Improve Subjective Sleep Quality After Total Knee Arthroplasty? A Randomized, Placebo-Controlled Trial. *Journal of Arthroplasty*, *39*(8), S15–S21. https://doi.org/10.1016/j.arth.2024.01.018
138. Lee, Y. C., Chibnik, L. B., Lu, B., Wasan, A. D., Edwards, R. R., Fossel, A. H., Helfgott, S. M., Solomon, D. H., Clauw, D. J., & Karlson, E. W. (2009). The relationship between disease activity, sleep, psychiatric distress and pain sensitivity in rheumatoid arthritis: A cross-sectional study. *Arthritis Research and Therapy*, *11*(5). https://doi.org/10.1186/ar2842
139. Lee, Y. C., Lu, B., Edwards, R. R., Wasan, A. D., Nassikas, N. J., Clauw, D. J., Solomon, D. H., & Karlson, E. W. (2013). The role of sleep problems in central pain processing in rheumatoid arthritis. *Arthritis and Rheumatism*, *65*(1), 59–68. https://doi.org/10.1002/art.37733
140. Lee, M. K., & Oh, J. H. (2019). The Relationship Between Pain and Physical Function: Mediating Role of Sleep Quality, Depression, and Fatigue*. Journal of gerontological nursing, 45*(7), 46–54. https://doi.org/10.3928/00989134-20190612-05
141. Lee, S. W., Oh, H. S., Kim, S. H., Park, W., Kwon, S. R., Lim, M. J., Jung, K. H., & Seo, W. S. (2022). Factors That Influence Sleep Disturbance and the Mediating Effects of Depression on Sleep Disturbance in Patients With Rheumatoid Arthritis. *Orthopaedic Nursing*, *41*(5), 335–344. https://doi.org/10.1097/NOR.0000000000000880
142. Lee, W. J., & Park, H. (2023). Effects of auricular acupressure on sleep and pain in elderly people who have osteoarthritis and live in nursing homes: A randomized, single-blind, placebo-controlled trial. *Explore*, *19*(2), 214–222. https://doi.org/10.1016/j.explore.2022.07.001
143. Leigh, T. J., Hindmarch, I., Bird, H. A., & Wright, V. (1988). Comparison of sleep in osteoarthritic patients and age and sex matched healthy controls. *Annals of the rheumatic diseases*, *47*(1), 40–42. https://doi.org/10.1136/ard.47.1.40
144. Leigh, T. J., Bird, H. A., Hindmarch, I., & Wright, V. (1989). The evaluation of a nonsteroidal anti-inflammatory drug (tenoxicam) in the treatment of sleep disturbance in osteoarthritic patients. *Clinical rheumatology*, *8*(4), 489–493. https://doi.org/10.1007/BF02032101
145. Lerman, S. F., Finan, P. H., Smith, M. T., & Haythornthwaite, J. A. (2017). Psychological interventions that target sleep reduce pain catastrophizing in knee osteoarthritis. *Pain*, *158*(11), 2189–2195. https://doi.org/10.1097/j.pain.0000000000001023
146. Liu, T., Geng, Y., Han, Z., Qin, W., Zhou, L., Ding, Y., Zhang, Z., & Sun, G. (2023). Self-reported sleep disturbance is significantly associated with depression, anxiety, self-efficacy, and stigma in Chinese patients with rheumatoid arthritis. *Psychology, Health and Medicine*, *28*(4), 908–916. https://doi.org/10.1080/13548506.2022.2039398
147. Loeppenthin, K., Esbensen, B. A., Klausen, J. M., Østergaard, M., Christensen, J. F., Tolver, A., Thomsen, T., Bech, J. S., & Jennum, P. (2022). Efficacy and Acceptability of Intermittent Aerobic Exercise on Polysomnography-Measured Sleep in People With Rheumatoid Arthritis With Self-Reported Sleep Disturbance: A Randomized Controlled Trial. *ACR Open Rheumatology*, *4*(5), 395–405. https://doi.org/10.1002/acr2.11403
148. Londhe, S. B., Shah, R. V., Antao, N., Londhe, I., Shah, A. R., Namjoshi, Z., Baranwal, G., & Singh, D. (2025). A prospective study comparing sleep quality using Pittsburgh Sleep Quality Index at 8 weeks after robotic-assisted versus conventional total knee arthroplasty: a single-center study. *Journal of Robotic Surgery*, *19*(1). https://doi.org/10.1007/s11701-025-02339-9
149. Lopes, F. H. A., Freitas, M. V. C., de Bruin, V. M. S., & de Bruin, P. F. C. (2021). Depressive symptoms are associated with impaired sleep, fatigue, and disease activity in women with rheumatoid arthritis. *Advances in Rheumatology*, *61*(1). https://doi.org/10.1186/s42358-021-00176-6
150. Løppenthin, K., Esbensen, B. A., Jennum, P., Østergaard, M., Christensen, J. F., Thomsen, T., Bech, J. S., & Midtgaard, J. (2014). Effect of intermittent aerobic exercise on sleep quality and sleep disturbances in patients with rheumatoid arthritis - Design of a randomized controlled trial. *BMC Musculoskeletal Disorders*, *15*(1). https://doi.org/10.1186/1471-2474-15-49
151. Løppenthin, K., Esbensen, B. A., Jennum, P., Østergaard, M., Tolver, A., Thomsen, T., & Midtgaard, J. (2015). Sleep quality and correlates of poor sleep in patients with rheumatoid arthritis. *Clinical Rheumatology*, *34*(12), 2029–2039. https://doi.org/10.1007/s10067-015-2875-4
152. Løppenthin, K., Esbensen, B. A., Østergaard, M., Jennum, P., Tolver, A., Aadahl, M., Thomsen, T., & Midtgaard, J. (2015). Physical activity and the association with fatigue and sleep in Danish patients with rheumatoid arthritis. *Rheumatology International*, *35*(10), 1655–1664. https://doi.org/10.1007/s00296-015-3274-5
153. Lošťák, J., Gába, A., Dygrýn, J., Baláž, Ĺ., Štefelová, N., & Gallo, J. (2022). Twenty-Four-Hour Movement Behaviors in Adults and Older Adults before Total Knee Arthroplasty: a Study Based on Compositional Data Analysis. 24hodinové pohybové chování u dospělých a starších dospělých před totální náhradou kolenního kloubu: studie založená na analýze kompozičních dat. *Acta chirurgiae orthopaedicae et traumatologiae Cechoslovaca*, *89*(1), 9–15.
154. Lü, J., Huang, L., Wu, X., Fu, W., & Liu, Y. (2017). Effect of Tai Ji Quan training on self-reported sleep quality in elderly Chinese women with knee osteoarthritis: a randomized controlled trail. *Sleep Medicine*, *33*, 70–75. https://doi.org/10.1016/j.sleep.2016.12.024
155. Lu, L., Liu, J., Mo, S., & Chen, H. (2019). The Effect of Auricular Plaster Therapy on Insomnia in Patients with Rheumatoid Arthritis. *Medical Acupuncture*, *31*(2), 130–133. https://doi.org/10.1089/acu.2019.1346
156. Lu, P. Y., Wu, H. Y., Chen, L. H., Liu, C. Y., & Chiou, A. F. (2023). The Effects of Self-Aromatherapy Massage on Pain and Sleep Quality in Patients with Rheumatoid Arthritis: A Randomized Controlled Trial. *Pain Management Nursing*, *24*(4), e52–e60. https://doi.org/10.1016/j.pmn.2023.02.009
157. Luyster, F. S., Chasens, E. R., Wasko, M. C., & Dunbar-Jacob, J. (2011). Sleep quality and functional disability in patients with rheumatoid arthritis. *Journal of clinical sleep medicine: JCSM: official publication of the American Academy of Sleep Medicine*, *7*(1), 49–55.
158. Lyne, L., Åkerstedt, T., Alfredsson, L., Lehtonen, T., Saevarsdottir, S., Klareskog, L., & Westerlind, H. (2022). Sleep problems in rheumatoid arthritis over 12 years from diagnosis: Results from the Swedish EIRA study. *RMD Open*, *8*(1). https://doi.org/10.1136/rmdopen-2021-001800
159. Maatallah, K., Makhlouf, Y., Ferjani, H., Cherif, I., Nessib, D. Ben, Triki, W., Kaffel, D., & Hamdi, W. (2022). Factors associated with the inflammatory process in pain in ankylosing spondylitis. *Pan African Medical Journal*, *41*. https://doi.org/10.11604/pamj.2022.41.331.31312
160. Mahowald, M. W., Mahowald, M. L., Bundlie, S. R., & Ytterberg, S. R. (1989). Sleep fragmentation in rheumatoid arthritis. *Arthritis and rheumatism*, *32*(8), 974–983. https://doi.org/10.1002/anr.1780320806
161. Manning, B. T., Kearns, S. M., Bohl, D. D., Edmiston, T., Sporer, S. M., & Levine, B. R. (2017). Prospective assessment of sleep quality before and after primary total joint replacement. *Orthopedics*, *40*(4), e636–e640. https://doi.org/10.3928/01477447-20170411-01
162. Mansoor, S., Zammurrad, S., & Rasheed, U. (2024). Evaluating the Impact of Sleep Disturbances and Depression on Axial Spondyloarthritis a Cross-Sectional Study. *Journal of Population Therapeutics and Clinical Pharmacology*. *31*(6), 1036-1041. https://doi.org/10.53555/jptcp.v31i6.6605
163. Manzo, C., & Castagna, A. (2020). Subjective sleep disturbances at the time of diagnosis in patients with polymyalgia rheumatica and in patients with seronegative elderly-onset rheumatoid arthritis. A pilot study. *Reumatologia*, *58*(4), 196–201. https://doi.org/10.5114/REUM.2020.98430
164. Martinez, R., Reddy, N., Mulligan, E. P., Hynan, L. S., Wells, J., & Benlidayi, I. C. (2019). Sleep quality and nocturnal pain in patients with hip osteoarthritis. *Medicine (United States)*, *98*(41). https://doi.org/10.1097/MD.0000000000017464
165. McCurry, S. M., Zhu, W., Von Korff, M., Wellman, R., Morin, C. M., Thakral, M., Yeung, K., & Vitiello, M. V. (2021). Effect of Telephone Cognitive Behavioral Therapy for Insomnia in Older Adults With Osteoarthritis Pain: A Randomized Clinical Trial. *JAMA internal medicine*, *181*(4), 530–538. https://doi.org/10.1001/jamainternmed.2020.9049
166. McKenna, S., Tierney, M., O’Neill, A., Fraser, A., & Kennedy, N. (2018). Sleep and physical activity: a cross-sectional objective profile of people with rheumatoid arthritis. *Rheumatology International*, *38*(5), 845–853. https://doi.org/10.1007/s00296-018-4009-1
167. McKenna, S. G., Donnelly, A., Esbensen, B. A., Comber, L., Ng, W. L., Anjum, A. M., Fraser, A., & Kennedy, N. M. (2021). The feasibility of an exercise intervention to improve sleep (time, quality and disturbance) in people with rheumatoid arthritis: a pilot RCT. *Rheumatology International*, *41*(2), 297–310. https://doi.org/10.1007/s00296-020-04760-9
168. Mehr, H. E., Mohammadizadeh, H., Rohi, F., Monfared, S. M., & Rajabi, F. (2023). Predicting Pain Perception Based on Psychological Distress in Patients with Rheumatoid Arthritis: The Mediating Role of Sleep Quality. In *J Sleep Sci* (Vol. 8). https://jss.tums.ac.ir
169. Mehta, N., Sancheti, P., Shyam, A., Patil, K., Gugale, S., Obaid, N., Sisodia, Y., & Sanghavi, S. (2025). Sleep quality improves after total knee arthroplasty: Addressing early disturbance and risk factors. *Journal of ISAKOS*, *10*. https://doi.org/10.1016/j.jisako.2024.100373
170. Mesci, E. (2016). Relation of physical activity level with quality of life, sleep and depression in patients with knee osteoarthritis. *Northern Clinics of Istanbul*. https://doi.org/10.14744/nci.2015.95867
171. Minnock, P., Veale, D. J., Bresnihan, B., FitzGerald, O., & McKee, G. (2015). Factors that influence fatigue status in patients with severe rheumatoid arthritis (RA) and good disease outcome following 6 months of TNF inhibitor therapy: a comparative analysis. *Clinical Rheumatology*, *34*(11), 1857–1865. https://doi.org/10.1007/s10067-015-3088-6
172. Miyauchi, K., Fujimoto, K., Abe, T., Takei, M., & Ogawa, K. (2021). Cross-sectional assessment of sleep and fatigue in middle-aged Japanese women with primary Sjogren syndrome or rheumatoid arthritis using self-reports and wrist actigraphy. *Medicine (United States)*, *100*(37). https://doi.org/10.1097/MD.0000000000027233
173. Mont, M. A., Lin, J. H., Spitzer, A. I., Dasa, V., Rivadeneyra, A., Rogenmoser, D., Concoff, A. L., Ng, M. K., DiGiorgi, M., DySart, S., Urban, J., & Mihalko, W. M. (2025). Improved Sleep Associated With Triamcinolone Acetonide Extended-Release Injections for Knee Osteoarthritis: Use of a New Real-World Registry. *Arthroplasty Today*, *32*. https://doi.org/10.1016/j.artd.2025.101655
174. Mukartihal, R. K., Angadi, D. S., Mangukiya, H. J., Singh, N. K., Varad, S., Ramesh, P. A., & Patil, S. S. (2022). Temporal changes in sleep quality and knee function following primary total knee arthroplasty: a prospective study. *International Orthopaedics*, *46*(2), 223–230. https://doi.org/10.1007/s00264-021-05192-1
175. Murphy, S. L., Lyden, A. K., Phillips, K., Clauw, D. J., & Williams, D. A. (2011). Association between pain, radiographic severity, and centrally-mediated symptoms in women with knee osteoarthritis. *Arthritis Care and Research*, *63*(11), 1543–1549. https://doi.org/10.1002/acr.20583
176. Mustafa, M., Bawazir, Y., Merdad, L., Wali, S., Attar, S., Fathaldin, O., Bahlas, S., Alhejaili, F., Aljohaney, A., Jan, A., & Jadu, F. (2019). Frequency of sleep disorders in patients with rheumatoid arthritis. *Open Access Rheumatology: Research and Reviews*, *11*, 163–171. https://doi.org/10.2147/OARRR.S201556
177. Myoji, Y., Fujita, K., Mawatari, M., & Tabuchi, Y. (2015). Changes in sleep-wake rhythms, subjective sleep quality and pain among patients undergoing total hip arthroplasty. *International Journal of Nursing Practice*, *21*(6), 764–770. https://doi.org/10.1111/ijn.12345
178. Nicassio, P. M., Ormseth, S. R., Kay, M., Custodio, M., Irwin, M. R., Olmstead, R., & Weisman, M. H. (2012). The contribution of pain and depression to self-reported sleep disturbance in patients with rheumatoid arthritis. *Pain*, *153*(1), 107–112. https://doi.org/10.1016/j.pain.2011.09.024
179. Nie, A., Wang, C., Song, Y., Xie, X., Yang, H., & Chen, H. (2018). Prevalence and factors associated with disturbed sleep in outpatients with ankylosing spondylitis. *Clinical Rheumatology*, *37*(8), 2161–2168. https://doi.org/10.1007/s10067-018-4190-3
180. Oksüz, S., & Unal, E. (2023). Comparison of the effects of aerobic training alone versus aerobic training combined with clinical Pilates exercises on the functional and psychosocial status of patients with ankylosing spondylitis: A randomized controlled trial. *Physiotherapy Theory and Practice*, *39*(1), 61–71. https://doi.org/10.1080/09593985.2021.2005199
181. Owens, M. A., Mun, C. J., Hamilton, K. R., Hughes, A., Campbell, C. M., Edwards, R. R., & Smith, M. T. (2023). Presurgical sleep and pain behaviors predict insomnia symptoms and pain after total knee arthroplasty: a 12-month longitudinal, observational study. *Pain Medicine (United States)*, *24*(11), 1224–1233. https://doi.org/10.1093/pm/pnad106
182. Ozkan, Y., & Kütük, S. G. (2016). Effect of severity of ankylosing spondylitis on psychiatric symptoms and quality of life. *Medical Science Technology*, *57*, 81–87. https://doi.org/10.12659/MST.898837
183. Palimi, T., Zilaee, M., Rajaei, E., & Karandish, M. (2022). Effects of Melatonin on Sleep Quality and Disease Activity in Patients With Rheumatoid Arthritis. *Sleep Medicine Research*, *13*(1), 38–45. https://doi.org/10.17241/smr.2022.01207
184. Parmelee, P. A., Cox, B. S., DeCaro, J. A., Keefe, F. J., & Smith, D. M. (2017). Racial/ethnic differences in sleep quality among older adults with osteoarthritis. *Sleep Health*, *3*(3), 163–169. https://doi.org/10.1016/j.sleh.2017.03.010
185. Pehlivan, S., Karadakovan, A., Pehlivan, Y., & Onat, A. M. (2016). Sleep quality and factors affecting sleep in elderly patients with rheumatoid arthritis in Turkey. *Turkish Journal of Medical Sciences*, *46*(4), 1114–1121. https://doi.org/10.3906/sag-1506-82
186. Petrov, M. E., Goodin, B. R., Cruz-Almeida, Y., King, C., Glover, T. L., Bulls, H. W., Herbert, M., Sibille, K. T., Bartley, E. J., Fessler, B. J., Sotolongo, A., Staud, R., Redden, D., Fillingim, R. B., & Bradley, L. A. (2015). Disrupted sleep is associated with altered pain processing by sex and ethnicity in knee osteoarthritis. *Journal of Pain*, *16*(5), 478–490. https://doi.org/10.1016/j.jpain.2015.02.004
187. Petzinna, S. M., Winter, L., Skowasch, D., Pizarro, C., Weber, M., Kütting, D., Behning, C., Bauer, C. J., & Schäfer, V. S. (2024). Assessing sleep-related breathing disorders among newly diagnosed rheumatoid and psoriatic arthritis patients: a cross-sectional study. *Rheumatology International*, *44*(6), 1025–1034. https://doi.org/10.1007/s00296-024-05610-8
188. Punshi, A., Prithiani, S., Das Talreja, M. L. K., Bai, K., Rasheed, U., & Bhaktiarpuri, A. (2023). Sleep Quality in Patients Presenting with Rheumatoid Arthritis at Tertiary Care Hospital, Islamabad. *Pakistan Journal of Medical and Health Sciences*, *17*(5), 131–133. https://doi.org/10.53350/pjmhs2023175131
189. Purabdollah, M., lakdizaji, S., & Rahmani, A. (2017). Relationship between Sleep, Pain and Inflammatory Markers in Patients with Rheumatoid Arthritis. *Journal of Caring Sciences*, *6*(3), 249–255. https://doi.org/10.15171/jcs.2017.024
190. Radwan, A., & Borai, A. (2021). Quality of sleep in rheumatoid arthritis patients: Relationship with disease activity, depression and functional status. *Egyptian Rheumatologist*, *43*(2), 183–187. https://doi.org/10.1016/j.ejr.2020.08.002
191. Rajalingam, S., Sakthiswary, R., & Hussein, H. (2017). Anti-citrullinated cyclic peptide antibody and functional disability are associated with poor sleep quality in rheumatoid arthritis. *Archives of Rheumatology*, *32*(1), 15–20. https://doi.org/10.5606/ArchRheumatol.2017.5960
192. Rezazadeh, M., Amini, M., Toufan, F., & Yousefi, Z. (2024). Investigating the Relationship between Sleep Quality and Pain Perception in Patients with Rheumatoid Arthritis. *Journal of Isfahan Medical School*, *42*(779), 703–710. https://doi.org/10.48305/jims.v42.i779.0703
193. Roehrs, T., Diederichs, C., Gillis, M., Burger, A. J., Stout, R. A., Lumley, M. A., & Roth, T. (2015). Effects of reduced time in bed on daytime sleepiness and recovery sleep in fibromyalgia and rheumatoid arthritis. *Journal of Psychosomatic Research*, *79*(1), 27–31. https://doi.org/10.1016/j.jpsychores.2015.03.010
194. Rosenthal, M., Moore, P., Groves, E., Iwan, T., Schlosser, L. G., Dziewanowska, Z., & Negro-Vilar, A. (2007). Sleep improves when patients with chronic OA pain are managed with morning dosing of once a day extended-release morphine sulfate (AVINZA): findings from a pilot study. *Journal of opioid management, 3*(3), 145–154. https://doi.org/10.5055/jom.2007.0052
195. Roth, T., Price, J. M., Amato, D. A., Rubens, R. P., Roach, J. M., & Schnitzer, T. J. (2009). The effect of eszopiclone in patients with Insomnia and coexisting rheumatoid arthritis: A pilot study. *Primary Care Companion to the Journal of Clinical Psychiatry*, *11*(6), 292–301. https://doi.org/10.4088/PCC.08m00749bro
196. Rudwaleit, M., Gooch, K., Michel, B., Herold, M., Thörner, Å., Wong, R., Kron, M., Chen, N., & Kupper, H. (2011). Adalimumab improves sleep and sleep quality in patients with active ankylosing spondylitis. *Journal of Rheumatology*, *38*(1), 79–86. https://doi.org/10.3899/jrheum.100213
197. Salwen, J. K., Smith, M. T., & Finan, P. H. (2017). Mid-treatment sleep duration predicts clinically significant knee osteoarthritis pain reduction at 6 months: Effects from a behavioral sleep medicine clinical trial. *Sleep*, *40*(2). https://doi.org/10.1093/sleep/zsw064
198. Sandikci, S. C., Colak, S., Aydoğan Baykara, R., Öktem, A., Cüre, E., Omma, A., & Kucuk, A. (2019). Evaluation of restless legs syndrome and sleep disorders in patients with psoriatic arthritis. *Zeitschrift Fur Rheumatologie*, *78*(10), 987–995. https://doi.org/10.1007/s00393-018-0562-y
199. Sarıyıldız. (2013). Sleep Quality in Rheumatoid Arthritis: Relationship Between the Disease Severity, Depression, Functional Status and the Quality of Life. *Journal of Clinical Medicine Research*. https://doi.org/10.4021/jocmr1648w
200. Sarvestan, J., Baker, K. F., & Del Din, S. (2025). Exploring the Effect of Sampling Frequency on Real-World Mobility, Sedentary Behaviour, Physical Activity and Sleep Outcomes Measured with Wearable Devices in Rheumatoid Arthritis: Feasibility, Usability and Practical Considerations. *Bioengineering*, *12*(1). https://doi.org/10.3390/bioengineering12010018
201. Sasaki, E., Tsuda, E., Yamamoto, Y., Maeda, S., Inoue, R., Chiba, D., Okubo, N., Takahashi, I., Nakaji, S., & Ishibashi, Y. (2014). Nocturnal knee pain increases with the severity of knee osteoarthritis, disturbing patient sleep quality. *Arthritis Care and Research*, *66*(7), 1027–1032. https://doi.org/10.1002/acr.22258
202. Schneeberger, E. E., Zaffarana, C. A., Cerda, O., & Citera, G. (2023). Sleep disturbances in patients with psoriatic arthritis. *Revista Colombiana de Reumatologia*, *30*, S34–S42. https://doi.org/10.1016/j.rcreu.2023.02.007
203. Shakya, H., Wang, D., Zhou, K., Luo, Z. Y., Dahal, S., & Zhou, Z. K. (2019). Prospective randomized controlled study on improving sleep quality and impact of zolpidem after total hip arthroplasty. *Journal of Orthopaedic Surgery and Research*, *14*(1). https://doi.org/10.1186/s13018-019-1327-2
204. Shen, B., Chen, H., Yang, D., Yolanda, O., Yuan, C., Du, A., Xu, R., Geng, Y., Chen, X., Li, H., & Xu, G. Y. (2021). A Structural Equation Model of Health-Related Quality of Life in Chinese Patients With Rheumatoid Arthritis. *Frontiers in Psychiatry*, *12*. https://doi.org/10.3389/fpsyt.2021.716996
205. Shourabi, E., Vagharseyyedin, S. A., Taghanaki, H. B., & Saremi, Z. (2024). The effect of self-administered acupressure on pain severity and sleep quality of patients with knee osteoarthritis: a randomized controlled trial. *BMC Complementary Medicine and Therapies*, *24*(1). https://doi.org/10.1186/s12906-024-04693-x
206. Sieper, J., Kivitz, A., Van Tubergen, A., Deodhar, A., Coteur, G., Woltering, F., & Landewé, R. (2015). Impact of Certolizumab Pegol on Patient-Reported Outcomes in Patients with Axial Spondyloarthritis. *Arthritis Care and Research*, *67*(10), 1475–1480. https://doi.org/10.1002/acr.22594
207. Singh, G., & Kumar, V. (2024). Sleep Quality is Poor in Rheumatoid Arthritis Patients and Correlates with Anxiety, Depression, and Poor Quality of Life. *Mediterranean Journal of Rheumatology*, *35*(3), 423–431. https://doi.org/10.31138/mjr.221022.sqp
208. Skougaard, M., Stisen, Z. R., Jørgensen, T. S., Egeberg, A., Hansen, R. L., Perez-Chada, L. M., Mogensen, M., Merola, J. F., Gerwien, J. G., & Kristensen, L. E. (2023). Increased prevalence of sleep disturbance in psoriatic arthritis is associated with inflammatory and non-inflammatory measures. *Scandinavian Journal of Rheumatology*, *52*(3), 259–267. https://doi.org/10.1080/03009742.2022.2044116
209. Solak, O., Dundar, U., Demirdal, U. S., Fidan, F., Gokce, I. Y., & Unlu, M. (2009). Evaluation of rheumatoid arthritis patients with Pittsburgh Sleep Quality Index/Romatoid artrit hastalarinin Pittsburgh Uyku Kalite Indeksi ile degerlendirilmesi. *Turkish Journal of Physical Medicine and Rehabilitation*, 107-111.
210. Solak, Ö., Fidan, F., Dündar, Ü., Türel, A., Ayçiçek, A., Kavuncu, V., & Ünlü, M. (2009). The prevalence of obstructive sleep apnoea syndrome in ankylosing spondylitis patients. *Rheumatology*, *48*(4), 433–435. https://doi.org/10.1093/rheumatology/kep021
211. Son, C. N., Choi, G., Lee, S. Y., Lee, J. M., Lee, T. H., Jeong, H. J., Jung, C. G., Kim, J. M., Cho, Y. W., & Kim, S. H. (2015). Sleep quality in rheumatoid arthritis, and its association with disease activity in a Korean population. *Korean Journal of Internal Medicine*, *30*(3), 384–390. https://doi.org/10.3904/kjim.2015.30.3.384
212. Song, Y., Wang, C., & Chen, H. (2017). Functional limitation and associated factors in outpatients with ankylosing spondylitis in Southwest China. *Clinical Rheumatology*, *36*(4), 871–877. https://doi.org/10.1007/s10067-017-3563-3
213. Song, B. W., Jeong, H. J., Kim, B. Y., Cho, Y. W., Son, C. N., Kim, S. S., & Kim, S. H. (2021). Bath Ankylosing Spondylitis Disease Activity Index is Associated with the Quality of Sleep in Ankylosing Spondylitis Patients. *Journal of Rheumatic Diseases*, *28*(3), 143–149. https://doi.org/10.4078/jrd.2021.28.3.143
214. Song, J., Muhammad, L. N., Neogi, T., Dunlop, D. D., Wohlfahrt, A., Bolster, M. B., Bingham, C. O., Clauw, D. J., Marder, W., & Lee, Y. C. (2023). Pain Sensitization as a Potential Mediator of the Relationship Between Sleep Disturbance and Subsequent Pain in Rheumatoid Arthritis. *Arthritis Care and Research*, *75*(4), 778–784. https://doi.org/10.1002/acr.24888
215. Spira, A. P., Runko, V. T., Finan, P. H., Kaufmann, C. N., Bounds, S. C., Liu, L., Buenaver, L. F., McCauley, L. M., Ancoli-Israel, S., & Smith, M. T. (2015). Circadian rest/activity rhythms in knee osteoarthritis with insomnia: A study of osteoarthritis patients and pain-free controls with insomnia or normal sleep. *Chronobiology International*, *32*(2), 242–247. https://doi.org/10.3109/07420528.2014.965314
216. Stocks, J., Tang, N. K. Y., Walsh, D. A., Warner, S. C., Harvey, H. L., Jenkins, W., Abhishek, A., Doherty, M., & Valdes, A. M. (2018). Bidirectional association between disturbed sleep and neuropathic pain symptoms: A prospective cohort study in post-total joint replacement participants. *Journal of Pain Research*, *11*, 1087–1093. https://doi.org/10.2147/JPR.S149830
217. Straub, R. H., Detert, J., Dziurla, R., Fietze, I., Loeschmann, P. A., Burmester, G. R., & Buttgereit, F. (2017). Inflammation Is an Important Covariate for the Crosstalk of Sleep and the HPA Axis in Rheumatoid Arthritis. *NeuroImmunoModulation*, *24*(1), 11–20. https://doi.org/10.1159/000475714
218. Suhas Masilamani, A. B., Jayakumar, T., Mulpur, P., Kikkuri, R. R., Vaddiparthi, S., & Gurava Reddy, A. V. (2025). Sleep quality after total knee arthroplasty: a comparative analysis of robotic-assisted and conventional techniques. *Journal of Robotic Surgery*, *19*(1). https://doi.org/10.1007/s11701-025-02405-2
219. Sveaas, S. H., Dagfinrud, H., Berg, I. J., Provan, S. A., Johansen, M. W., Pedersen, E., & Bilberg, A. (2020). High-Intensity Exercise Improves Fatigue, Sleep, and Mood in Patients With Axial Spondyloarthritis: Secondary Analysis of a Randomized Controlled Trial. *Physical therapy*, *100*(8), 1323–1332. https://doi.org/10.1093/ptj/pzaa086
220. Szady, P., Baczyk, G., & Kozłowska, K. (2017). Fatigue and sleep quality in rheumatoid arthritis patients during hospital admission. *Reumatologia*, *55*(2), 65–72. https://doi.org/10.5114/reum.2017.67600
221. Tabaraii, R., Masoumi, M., Bagherzadeh-Fard, M., Yazdanifar, M. A., Balasi, J., & Smiley, A. (2021). Association of lifestyle and disease characteristics with self-rated wellness/health score in patients with rheumatoid arthritis. *BMC Rheumatology*, *5*(1). https://doi.org/10.1186/s41927-021-00227-x
222. Taibi, D. M., Bourguignon, C., & Gill Taylor, A. (2009). A feasibility study of valerian extract for sleep disturbance in person with arthritis. *Biological Research for Nursing*, *10*(4), 409–417. https://doi.org/10.1177/1099800408324252
223. Taibi, D. M., & Vitiello, M. V. (2011). A pilot study of gentle yoga for sleep disturbance in women with osteoarthritis. *Sleep Medicine*, *12*(5), 512–517. https://doi.org/10.1016/j.sleep.2010.09.016
224. Tamm, S., Lensmar, C., Andreasson, A., Axelsson, J., Forsberg Morén, A., Grunewald, J., Gyllfors, P., Karshikoff, B., Kosek, E., Lampa, J., Olgart Höglund, C., Strand, V., Cervenka, S., & Lekander, M. (2021). Objective and Subjective Sleep in Rheumatoid Arthritis and Severe Seasonal Allergy: Preliminary Assessments of the Role of Sickness, Central and Peripheral Inflammation. *Nature and science of sleep*, *13*, 775–789. https://doi.org/10.2147/NSS.S297702
225. Tang, H. Y. (Jean), McCurry, S. M., Pike, K. C., Riegel, B., & Vitiello, M. V. (2021). Open-loop Audio-Visual Stimulation for sleep promotion in older adults with comorbid insomnia and osteoarthritis pain: results of a pilot randomized controlled trial. *Sleep Medicine*, *82*, 37–42. https://doi.org/10.1016/j.sleep.2021.03.025
226. Tang, H. Y. (Jean), McCurry, S. M., Pike, K. C., Von Korff, M., & Vitiello, M. V. (2017). Differential predictors of nighttime and daytime sleep complaints in older adults with comorbid insomnia and osteoarthritis pain. *Journal of Psychosomatic Research*, *100*, 22–28. https://doi.org/10.1016/j.jpsychores.2017.06.020
227. Taylor-Gjevre, R. M., Gjevre, J. A., Nair, B., Skomro, R., & Lim, H. J. (2011). Components of sleep quality and sleep fragmentation in rheumatoid arthritis and osteoarthritis. *Musculoskeletal care*, *9*(3), 152–159. https://doi.org/10.1002/msc.208
228. Taylor-Gjevre, R. M., Gjevre, J. A., Skomro, R. P., Nair, B. V., & Lim, H. J. (2011). Improved sleep efficiency after anti-tumor necrosis factor α therapy in rheumatoid arthritis patients. *Therapeutic Advances in Musculoskeletal Disease*, *3*(5), 227–233. https://doi.org/10.1177/1759720X11416862
229. Taylor, S. S., Hughes, J. M., Coffman, C. J., Jeffreys, A. S., Ulmer, C. S., Oddone, E. Z., Bosworth, H. B., Yancy, W. S., & Allen, K. D. (2018). Prevalence of and characteristics associated with insomnia and obstructive sleep apnea among veterans with knee and hip osteoarthritis. *BMC Musculoskeletal Disorders*, *19*(1). https://doi.org/10.1186/s12891-018-1993-y
230. Tektonidou, M. G., Katsifis, G., Georgountzos, A., Theodoridou, A., Koukli, E. M., Kandili, A., Giokic-Kakavouli, G., & Karatsourakis, T. D. (2020). Real-world evidence of the impact of adalimumab on work productivity and sleep measures in patients with rheumatoid arthritis, psoriatic arthritis, and ankylosing spondylitis. *Therapeutic Advances in Musculoskeletal Disease*, *12*. https://doi.org/10.1177/1759720X20949088
231. Thorlund, J. B., Skarpsno, E. S., Vestergaard, J. J., Skou, S. T., Grønne, D. T., Roos, E. M., & Vaegter, H. B. (2025). Sleep problems and insomnia are common and associated with pain intensity, number of comorbidities and analgesic use in patients with knee and hip osteoarthritis: a cross-sectional study using data from the good life with osteoarthritis in Denmark (GLA:D®) registry. *Rheumatology International*, *45*(5). https://doi.org/10.1007/s00296-025-05878-4
232. Toledano, E., Gómez-Lechón, L., Chacón, C. C., Hidalgo, C., Ibáñez, M., Márquez, A., Queiro, R., & Montilla, C. (2024). Clinical Features and Disease Activity in Psoriatic Arthritis: A Sex-Related Perspective on Leptin and Comorbidity. *Journal of Clinical Medicine*, *13*(10). https://doi.org/10.3390/jcm13102959
233. Toledano, E., Hidalgo, C., Gómez-Lechón, L., ibáñez, M., Chacón, C. C., Martín-Vallejo, J., Pastor, S., & Montilla, C. (2023). SLEEP quality in patients with psoriatic arthritis and its relationship with disease activity and comorbidities: a cross-sectional study. *Scientific Reports*, *13*(1). https://doi.org/10.1038/s41598-023-48723-z
234. Tuncay, M. S., Sahin, O., Semiz, M., Semiz, E., Alim, B., Peksen, H., Salihoglu, S., Sabuncu, M. F., Kilic, M. S., & Tavasli, A. (2024). Evaluation of sleep quality in rheumatoid arthritis patients. *Annals of Clinical and Analytical Medicine*, *15*(05). https://doi.org/10.4328/acam.22050
235. Tymms, K., Butcher, B. E., Sletten, T. L., Smith, T., O’Sullivan, C., Littlejohn, G., Sadler, R., Tronnberg, R., & Griffiths, H. (2022). Prevalence of sleep disturbance and the association between poor disease control in people with ankylosing spondylitis within the Australian clinical setting (ASLEEP study): a real-world observational study using the OPAL dataset. *Clinical Rheumatology*, *41*(4), 1105–1114. https://doi.org/10.1007/s10067-021-05953-8
236. Ulus, Y., Akyol, Y., Tander, B., Durmus, D., Bilgici, A., & Kuru, O. (2011). Sleep quality in fibromyalgia and rheumatoid arthritis: associations with pain, fatigue, depression, and disease activity. *Clinical and experimental rheumatology, 29*(6 Suppl 69), S92–S96.
237. Ünal, M., & Durmus, D. (2016). The effect of capsaicin phonophoresis in knee osteoarthritis and can it be utilized early in primary care? : A randomized-controlled trial. *Konuralp Medical Journal,* *8*(3), 173-180. https://doi.org/10.18521/ktd.284442
238. Urkmez, B., & Keskin, Y. (2020). Relationship between sleep quality and physical activity level in patients with ankylosing spondylitis. *Modern Rheumatology*, *30*(6), 1053–1059. https://doi.org/10.1080/14397595.2019.1702139
239. Van Meirhaeghe, J. P., Salmon, L. J., O’Sullivan, M. D., Gooden, B. R., Lyons, M. C., Pinczewski, L. A., & Roe, J. P. (2021). Improvement in Sleep Patterns After Hip and Knee Arthroplasty: A Prospective Study in 780 Patients. *Journal of Arthroplasty*, *36*(2), 442–448. https://doi.org/10.1016/j.arth.2020.08.056
240. Viitanen, J., Ronni, S., Ala-Peijari, S., Uoti-Reilama, K., & Kautiainen, H. (2000). A comparison of self-estimated symptoms and impact of disease in fibromyalgia and rheumatoid arthritis. *Journal of Musculoskeletal Pain*, *8*(3), 21–33. https://doi.org/10.1300/J094v08n03_03
241. Vitiello, M. V., McCurry, S. M., Shortreed, S. M., Baker, L. D., Rybarczyk, B. D., Keefe, F. J., & Von Korff, M. (2014). Short-term improvement in insomnia symptoms predicts long-term improvements in sleep, pain, and fatigue in older adults with comorbid osteoarthritis and insomnia. *Pain*, *155*(8), 1547–1554. https://doi.org/10.1016/j.pain.2014.04.032
242. Vitiello, M. V., Rybarczyk, B., Von Korff, M., & Stepanski, E. J. (2009). Cognitive behavioral therapy for insomnia improves sleep and decreases pain in older adults with co-morbid insomnia and osteoarthritis. *Journal of clinical sleep medicine: JCSM: official publication of the American Academy of Sleep Medicine*, *5*(4), 355–362.
243. Vitiello, M. V., Zhu, W., Von Korff, M., Wellman, R., Morin, C. M., Yeung, K., & McCurry, S. M. (2022). Long-term improvements in sleep, pain, depression, and fatigue in older adults with comorbid osteoarthritis pain and insomnia. *Sleep*, *45*(2). https://doi.org/10.1093/sleep/zsab231
244. Von Korff, M., Vitiello, M. V., McCurry, S. M., Balderson, B. H., Moore, A. L., Baker, L. D., Yarbro, P., Saunders, K., Keefe, F. J., & Rybarczyk, B. D. (2012). Group interventions for co-morbid insomnia and osteoarthritis pain in primary care: The lifestyles cluster randomized trial design. *Contemporary Clinical Trials*, *33*(4), 759–768. https://doi.org/10.1016/j.cct.2012.03.010
245. Wadeley, A., Clarke, E., Leverment, S., & Sengupta, R. (2018). Sleep in ankylosing spondylitis and non-radiographic axial spondyloarthritis: associations with disease activity, gender and mood. *Clinical Rheumatology*, *37*(4), 1045–1052. https://doi.org/10.1007/s10067-018-3984-7
246. Waheed, Z., & Kalsoom, U. (2024). The Prevalence of Anxiety, Depression, and Sleep Disturbances Among the Rheumatoid Arthritis Patients. *Journal of Postgraduate Medical Institute*, *38*(1), 54–58. https://doi.org/10.54079/jpmi.38.1.3282
247. Ward, L., Stebbings, S., Athens, J., Cherkin, D., & David Baxter, G. (2018). Yoga for the management of pain and sleep in rheumatoid arthritis: a pilot randomized controlled trial. *Musculoskeletal Care*, *16*(1), 39–47. https://doi.org/10.1002/msc.1201
248. Wells, G., Li, T., & Tugwell, P. (2010). Investigation into the impact of abatacept on sleep quality in patients with rheumatoid arthritis, and the validity of the MOS-Sleep questionnaire Sleep Disturbance Scale. *Annals of the Rheumatic Diseases*, *69*(10), 1768–1773. https://doi.org/10.1136/ard.2009.119727
249. Westhovens, R., Van Der Elst, K., Matthys, A., Tran, M., & Gilloteau, I. (2014). Sleep problems in patients with rheumatoid arthritis. *Journal of Rheumatology*, *41*(1), 31–40. https://doi.org/10.3899/jrheum.130430
250. Whibley, D., Braley, T. J., Kratz, A. L., & Murphy, S. L. (2019). Transient Effects of Sleep on Next-Day Pain and Fatigue in Older Adults With Symptomatic Osteoarthritis. *Journal of Pain*, *20*(11), 1373–1382. https://doi.org/10.1016/j.jpain.2019.04.011
251. Wolfe, F., Michaud, K., & Li, T. (2006). Personal non-commercial use only. In *The Journal of Rheumatology* (Vol. 33, Issue 10). www.jrheum.org
252. Wong, I. T. Y., Chandran, V., Li, S., & Gladman, D. D. (2017). Sleep disturbance in psoriatic disease: Prevalence and associated factors. *Journal of Rheumatology*, *44*(9), 1369–1374. https://doi.org/10.3899/jrheum.161330
253. Xu, T., Jia, X., Chen, S., Xie, Y., Tong, K. K., Iezzi, T., & Jackson, T. (2021). Physical activity and sleep differences between osteoarthritis, rheumatoid arthritis and non-arthritic people in China: objective versus self-report comparisons. *BMC Public Health*, *21*(1). https://doi.org/10.1186/s12889-021-11837-y
254. Yaseen, A., Yasin, S., Ul Haq, Z., Ahmad, I., Hussain, M., & Pervez, N. (2024). Frequency of Sleep Disorders in Rheumatoid Arthritis Patients. *Cureus*. https://doi.org/10.7759/cureus.72417
255. Yeung, W. K., Morgan, K., & Mckenna, F. (2018). Comparison of sleep structure and psychometric profiles in patients with fibromyalgia, osteoarthritis and healthy controls. *Journal of Sleep Research*, *27*(2), 290–298. https://doi.org/10.1111/jsr.12588
256. Yilmaz, T., Başpinar, M. M., Arslan, N. G., Yilmaz Yalçinkaya, E., & Basat, O. (2021). Is There an Effect of Pain and Radiological Stage on Quality of Life and Sleep in Patients with Osteoarthritis? *Fiziksel Tıp ve Rehabilitasyon Bilimleri Dergisi*, *24*(2), 97–106. https://doi.org/10.31609/jpmrs.2020-77436
257. Ying, X., Zhao, Q., Wu, Y., Deng, S., Ma, Q., & Fang, R. (2025). Analysis of sleep disorders and their influencing factors in patients with ankylosing spondylitis. *PLoS ONE*, *20*(5 May). https://doi.org/10.1371/journal.pone.0323324
258. Yüksel, G. A., Kurtuluş, D., & Tireli, H. (2019). Sleep Quality and Restless Legs Syndrome in Patients with Ankylosing Spondylitis. *Haydarpasa Numune Med J, 59*(1), 78-83. https://doi.org/10.14744/hnhj.2018.93723.
259. Yolbaş, S., Yildirim, A., Düzencı, D., Gündoğdu, B., Özgen, M., & Koca, S. S. (2017). Sleep quality, sleeping postures, and sleeping equipment in patients with ankylosing spondylitis. *Turkish Journal of Medical Sciences*, *47*(4), 1198–1205. https://doi.org/10.3906/sag-1605-62
260. Yousefi, S., Zanjani, Z., Omidi, A., Zamani, B., & Sayyah, M. (2023). Comparison of Mindfulness-Based Stress Reduction Therapy and Cognitive-Behavioral Therapy of Chronic Fatigue, Sleep Quality, Executive Function and Disease Activity in Patients with Rheumatoid Arthritis. *Journal of Contemporary Psychotherapy*, *53*(2), 173–180. https://doi.org/10.1007/s10879-022-09558-3
261. Yüce, E., Şentürk, E., Sağaltici, E., Şentürk, İ. A., & Aytekin, E. (2023). Sleep quality and depression in patients with ankylosing spondylitis and their associations with clinical parameters: A cross-sectional, case-control study. *Agri*, *35*(1), 1–9. https://doi.org/10.14744/agri.2021.46514
262. Zamarrón, C., Maceiras, F., Mera, A., & Gómez-Reino, J. J. (2004). Effect of the first infliximab infusion on sleep and alertness in patients with active rheumatoid arthritis. *Annals of the Rheumatic Diseases*, *63*(1), 88–90. https://doi.org/10.1136/ard.2003.007831
263. Zhang, S., Chen, Z., Wu, Y., Lin, D., He, J., Liu, J., Gao, F., & Lin, H. (2021). Efficacy and safety of thalidomide on psychological symptoms and sleep disturbances in the patient with refractory ankylosing spondylitis. *Annals of Palliative Medicine*, *10*(3), 2512–2519. https://doi.org/10.21037/apm-19-409
264. Zhu, X., Xu, H., Wang, L., Wang, J., & Zhang, H. (2023). Relationship of preoperative sleep quality and early rehabilitation after unicompartmental knee arthroplasty. *Chinese Journal of Tissue Engineering Research*, *27*(36), 5806–5811. https://doi.org/10.12307/2023.706
265. Zúñiga-Vera, A., Quiñonez-Caicedo, J., & Martínez-Pérez, J. (2023). Correlation between sleep quality and disease activity in patients with Spondyloarthritis. *Revista Ecuatoriana de Neurologia*, *32*(3), 47–51. https://doi.org/10.46997/revecuatneurol32300047
