# Supplementary material for: Sleep measurement and clinical impact in osteoarthritis and inflammatory arthritis: a scoping review
Source: Rheumatology (Oxford). 2026 Aug 11;65(8):keag412. doi: 10.1093/rheumatology/keag412 (PMC13523860; doi:10.1093/rheumatology/keag412)
Supplement: Supplementary file 1 [file keag412_supplementary_data.zip › Supplement_Data.zip!/rhe-25-3089-File007.docx]

**Supplementary Data S4**

**Clinical Characteristics of the Included Study Populations**

The most frequently investigated population was individuals with RA, reported in 110 (41.5%) studies. OA was investigated in 77 studies (29.1%), including mixed-joint OA without specification on the joint(s) (56/77; 72.7%), knee OA (KOA, 16/77; 20.8%), hand OA (1/77; 1.3%), hip OA (1/77; 1.3%), and hip or hand OA combined (3/77; 3.9%). Spondyloarthritis (SpA) and its subtypes were represented in 61 (21.9%) studies. Among these, four (6.6%) studies addressed SpA in general, while most focused on AxSpA: 15 (24.6%) on AxSpA, 28 (49.5%) referred to ankylosing spondylitis (AS) or radiographic AxSpA, and 2 (3.3%) on non-radiographic AxSpA. Two (3.3%) studies included both radiographic and non-radiographic forms of SpA within the same design. Additionally, eight studies (13.1%) investigated PsA, and one study (1.6%) involved both PsA and AxSpA. A subset of studies (17/265; 6.4%) compared populations with different rheumatologic diagnoses, with the most common comparisons being between RA and PsA, RA and OA, and RA and fibromyalgia, and frequently included healthy control groups.
