# Supplementary material for: Sleep measurement and clinical impact in osteoarthritis and inflammatory arthritis: a scoping review
Source: Rheumatology (Oxford). 2026 Aug 11;65(8):keag412. doi: 10.1093/rheumatology/keag412 (PMC13523860; doi:10.1093/rheumatology/keag412)
Supplement: Supplementary file 1 [file keag412_supplementary_data.zip › Supplement_Data.zip!/rhe-25-3089-File008.docx]

**Supplementary Table S1**

**Records excluded with reasons**

| **First author, year** | **Title** | **Reason for exclusion** |
| --- | --- | --- |
| Abad, 2014 | Sleep deprivation and rheumatologic disease | Wrong study design |
| Abbasi, 2013 | Sleep disturbances in patients with rheumatoid arthritis. | No full text |
| Aissaoui, 2012 | Fatigue in patients with ankylosing spondylitis: prevalence and relationships with disease-specific variables, psychological status, and sleep disturbance. | Sleep not measured with a validated outcome measure |
| Alamoudi, 2006 | Sleep-disordered breathing in patients with acquired retrognathia secondary to rheumatoid arthritis | Wrong patient population |
| Alegre De Mique, 2005 | Rheumatism and sleep | Wrong study design |
| Allam, 2011 | Sleep disturbance in cervical spondylosis | No full text |
| Alonso, 2023 | Altered sleep in axial spondyloarthritis and psoriatic arthritis: Post hoc comparative study based on a sleep-specific question from the ASAS health index | Sleep not measured with a validated outcome measure |
| Alshehri, 2025 | Combined Diabetes and Arthritis are Associated with Increased Sleep Efficiency Variability | Self-reported IA/OA diagnosis |
| Appelbaum, 1988 | Cognitive behavioral treatment of a veteran population with moderate to severe rheumatoid arthritis | Sleep not measured with a validated outcome measure |
| Ataka, 2010 | Occipitocervical fusion has potential to improve sleep apnea in patients with rheumatoid arthritis and upper cervical lesions. | Wrong study design |
| Avila-Ribeiro, 2017 | Psychometric properties of sleep and coping numeric rating scales in rheumatoid arthritis: a subanalysis of an etanercept trial | Topic not alligned with the review scope |
| Bartlett, 2020 | Associations Among Fatigue, Vitality, Sleep, and HRQL in Patients with Rheumatoid Arthritis: data from Phase 3 Trials of Tofacitinib | Wrong study design |
| Batmazi, 2013 | Sleep quality and associated factors in ankylosing spondylitis: relationship with disease parameters, psychological status and quality of life | Wrong patient population |
| Belza, 1995 | Comparison of self-reported fatigue in rheumatoid arthritis and controls | No full text |
| Berkay, 2024 | The Impact of Diagnosed Chronic Sleep Disorders on Outcomes Following Total Knee Arthroplasty | Sleep not measured with a validated outcome measure |
| Bettega, 1998 | Surgical treatment of a patient with obstructive sleep apnea syndrome associated with temporomandibular joint destruction by rheumatoid arthritis | Wrong study design |
| Brahem, 2021 | Evaluation of sleep disorders in knee osteoarthritis (Epworth and Pittsburg scales) | No full text |
| Bui, 2016 | The contribution of sleep disturbance and inflammation to Psychological distress and pain in rheumatoid arthritis | Wrong study design |
| Caldwell, 2002 | Efficacy and safety of a once-daily morphine formulation in chronic, moderate-to-severe osteoarthritis pain: results from a randomized, placebo-controlled, double-blind trial and an open-label extension trial | Sleep not measured with a validated outcome measure |
| Carlson, 1998 | Nsaids improve quality of sleep in patients with osteoarthritis pain | No full text |
| Chen, 2012 | The investigation and measurement of quality of sleep in individuals with osteoarthritis in Taiwan: a cross-sectional survey | Wrong study design |
| Chen, 2024 | Sleep characteristics and risk of osteoarthritis: A two-sample and multivariate Mendelian randomization study | Sleep not measured with a validated outcome measure |
| ChiCTR2000035754 | Effects of opioid and opioid-free analgesia on sleep and neuropsychiatric performance in elderly patients after knee arthroplasty: a randomized controlled study | Wrong patient population |
| ChiCTR2500096906 | The Effect of improving sleep quality during dexmedetomidine and ramazolam on sleep disorders in elderly patients after knee replacement surgery | Diagnosis of IA/OA not clinically verified |
| ChiCTR2500099848 | Effect of perioperative subanesthetic esketamine infusion on postoperative sleep quality in patients after total joint arthroplasty: a randomized controlled trial | Wrong patient population |
| ChiCTR-IOR-16007861 | Sleep quality affects early recovery following total hip arthroplasty, A Randomized, Double-blind, Controlled Trail | Trial record not retrievable |
| Cho, 2020 | Association between sleep duration and osteoarthritis and their prevalence in Koreans: A cross-sectional study | Sleep not measured with a validated outcome measure |
| Çınar, 2023 | Sleep Quality in Patients with Ankylosing Spondylitis | Sleep not measured with a validated outcome measure |
| Coksevim, 2017 | Effects of global postural reeducation exercise and anti-TNF treatments on disease activity, fatigue, mobility, sleep quality and depression in patients with active ankylosing spondylitis (prospective-controlled trial) | Wrong study design |
| Crouthamel, 2021 | Developing a Novel Measurement of Sleep in Rheumatoid Arthritis: Study Proposal for Approach and Considerations | Topic not alligned with the review scope |
| Dai, 2020 | Sleep Quality Is Related to Worsening Knee Pain in Those with Widespread Pain: The Multicenter Osteoarthritis Study | Wrong patient population |
| Dautovich. 2015 | Breaking the cycle: Sleep disturbance as a target for remedying the 'cycle of distress' in osteoarthritis | Wrong study design |
| Deodhar, 2017 | Improvements in sleep problems and pain in patients with active ankylosing spondylitis treated with intravenous golimumab: 28-week results of the phase III trial | Wrong study design |
| Devins, 1993 | Restless sleep, illness intrusiveness, and depressive symptoms in three chronic illness conditions: rheumatoid arthritis, end-stage renal disease, and multiple sclerosis | Sleep not measured with a validated outcome measure |
| Drewes, 2000 | A longitudinal study of clinical symptoms and sleep parameters in rheumatoid arthritis | Wrong study design |
| Druce, 2018 | Quality of life, sleep and rheumatoid arthritis (QUASAR): a protocol for a prospective UK mhealth study to investigate the relationship between sleep and quality of life in adults with rheumatoid arthritis | Self-reported IA/OA diagnosis |
| Dumith, 2021 | Who are the individuals with the worst perceived quality of sleep? A population-based survey in southern Brazil | Wrong patient population |
| Duruöz, 2018 | The Validity and Reliability of Turkish Version of the Jenkins Sleep Evaluation Scale in Rheumatoid Arthritis | Wrong study design |
| Duruöz, 2018 | Validity and reliability of the Turkish version of the Jenkins Sleep Scale in psoriatic arthritis | Wrong study design |
| Feehan, 2020 | Twenty-Four Hour Activity and Sleep Profiles for Adults Living with Arthritis: Habits Matter | Wrong patient population |
| Ferguson, 1996 | Broken sleep, pain, disability, social activity, and depressive symptoms in rheumatoid arthritis | Sleep not measured with a validated outcome measure |
| Filchenko, 2021 | [Self-reported sleep and wake disorders in patients with ostheoarthrosis and end-stage renal disease] | Wrong patient population |
| Finn, 1998 | Sleep-disordered breathing and self-reported general health status in the Wisconsin Sleep Cohort Study | Wrong patient population |
| Frange, 2015 | Circadian rhythms, insomnia and osteoarthritis pain: Comments on article titled "Circadian rest/activity rhythms in knee osteoarthritis with insomnia: A study of osteoarthritis patients and pain-free controls with insomnia or normal sleep" | Wrong study design |
| Franke, 2021 | Osteoarthritis and sleep disorders: Cognitive behavioral therapy is helpful | Wrong study design |
| Freburger 2010 | The effects of a physical activity program on sleep- and health-related quality of life in older persons with arthritis | Self-reported IA/OA diagnosis |
| Gallego-Ariza, 2013 | Impact of a supervised muscular training on perceived quality of sleep and health in a population with knee osteoarthritis | Wrong study design |
| Gao, 2022 | Association Between Sleep Traits and Rheumatoid Arthritis: A Mendelian Randomization Study | Wrong patient population |
| Garver, 2015 | Integrating lifestyle approaches into osteoarthritis care | Wrong study design |
| Gerber, 2020 | Polyarticular arthritis, independent of rheumatoid factor, is associated with poor functional outcome in recent onset inflammatory synovitis | Sleep not measured with a validated outcome measure |
| Ghanmi, 2023 | Sleep Disorders in Patients Affected With Rheumatoid Arthritis: Assessment and Correlation with Flare-Ups | Wrong study design |
| Gilbert, 2021 | Relationship Between Self-Reported Restless Sleep and Objectively Measured Physical Activity in Adults With Knee Osteoarthritis | Sleep not measured with a validated outcome measure |
| Ginta, 2023 | Osteoarthritis and the sleep connection: managing and thriving when joints are affected | Wrong study design |
| Gjevre, 2023 | Restless Legs Syndrome as a Comorbidity in Rheumatoid Arthritis | Wrong study design |
| Gona, 2024 | A Study on Assessment of Depression and Sleep Disturbances among Patients with Rheumatoid Arthritis | Retracted article |
| Haber, 2023 | Relationships between sleep quality and hip pain severity in people with hip osteoarthritis | No full text |
| Habib, 2021 | The Effect of Medical Cannabis on Pain Level and Quality of Sleep among Rheumatology Clinic Outpatients | Wrong patient population |
| Hakkou, 2013 | Sleep disturbance in Moroccan patients with ankylosing spondylitis: prevalence and relationships with disease-specific variables, psychological status and quality of life | Sleep not measured with a validated outcome measure |
| Hamai, 2023 | Interaction between functional capability and sleep quality at midterm after total knee arthroplasty: a Japanese retrospective cohort study | Wrong patient population |
| Hamilton, 1999 | Sleep apnoea caused by rheumatoid arthritis | Wrong study design |
| Heffner, 2016 | Sleep improvement in older adults with osteoarthritis is associated with lower inflammatory response to laboratory pain and improved clinical pain | Wrong study design |
| Huber, 2025 | Neighborhood Disadvantage and Knee Osteoarthritis Pain: Do Sleep and Catastrophizing Play a Role? | Wrong patient population |
| IRCT20191027045257N7 | Investigation of the Impact of a Lower Back Recovery Device on Sleep Quality and Blood Glucose Levels in Diabetic Patients with Knee Osteoarthritis | Trial record not retrievable |
| IRCT20210129050172N1 | Investigating the effect of exercise program on improving sleep quality and quality of life | Trial record not retrievable |
| IRCT20211221053475N1 | The effect of acupressure on pain intensity and sleep quality in patients with knee osteoarthritis | Related to an included article |
| IRCT20230304057605N1 | The effect of Benson relaxation technique on sleep quality, anxiety, stress and depression in the elderly with knee osteoarthritis | Wrong patient population |
| Irwin, 2012 | Sleep loss exacerbates fatigue, depression, and pain in rheumatoid arthritis | Related to an included article |
| ISRCTN14233189 | The REST feasibility trial: sleep and pain in patients undergoing total knee replacement | Related to an included article |
| Jamieson, 1995 | The effect of sleep and nocturnal movement on stiffness, pain, and psychomotor performance in ankylosing spondylitis | No full text |
| Jones, 2021 | The BSR-psa: study protocol for the British Society for Rheumatology psoriatic arthritis register | Wrong study design |
| Jung, 2018 | The association between osteoarthritis and sleep duration in Koreans: a nationwide cross-sectional observational study | Sleep not measured with a validated outcome measure |
| Kaeley, 2015 | Similar improvements in quality of sleep, satisfaction with medication and sexual impairment among rheumatoid arthritis patients upon initiation of adalimumab in combination with two doses of methotrexate | Wrong study design |
| KCT0007502 | Effects of Auricular Acupressure on Pain and Sleep of Older Adults undergoing Osteoarthritis in Long-term Care Facilities | Trial record not retrievable |
| Kessing, 2020 | Study of the relationship between arthritis, inflammation and good sleep | Wrong study design |
| Kim, 2016 | Association of sleep duration with rheumatoid arthritis in Korean adults: analysis of seven years of aggregated data from the Korea National Health and Nutrition Examination Survey (KNHANES) | Wrong patient population |
| Kratz, 2018 | (123) - Are nights of poor sleep related to greater next-day fatigue and pain in older adults with symptomatic osteoarthritis? An actigraphy and ecological momentary assessment study | No full text |
| Labie, 2024 | Description and Evaluation of a Commercially Available Smartwatch Device for Sleep Outcomes in People with Knee Osteoarthritis and Insomnia | Wrong study design |
| Lapane, 2021 | The relationship between restless sleep and symptoms of the knee: data from the Osteoarthritis Initiative | Sleep not measured with a validated outcome measure |
| Latocha, 2020 | Cognitive behavioural therapy for insomnia in patients with rheumatoid arthritis: protocol for the randomised, single-blinded, parallel-group Sleep-RA trial | Related to an included study |
| Latocha, 2022 | The Effect of Group-Based Cognitive Behavioural Therapy for Insomnia in Patients with Rheumatoid Arthritis: a Randomised Controlled Trial | Wrong study design |
| Lavie, 1992 | Actigraphic measurements of sleep in rheumatoid arthritis: comparison of patients with low back pain and healthy controls | No full text |
| Leigh, 1988 | Measurement of nocturnal body motility: behaviour of osteoarthritic patients and healthy controls | Wrong outcomes |
| Leigh, 1987 | A comparison of sleep in rheumatic and non-rheumatic patients | No full text |
| Li, 2024 | Causal association between sleep traits and autoimmune arthritis: Evidence from a bidirectional Mendelian randomization study | Wrong patient population |
| Li, 2012 | Sleep disturbances are associated with increased pain, disease activity, depression, and anxiety in ankylosing spondylitis: a case-control study | Wrong patient population |
| Loppenthin, 2019 | Efficacy of intermittent aerobic exercise on sleep efficiency in patients with rheumatoid arthritis. A randomized controlled trial | Wrong study design |
| Louie, 2011 | Sleep disturbances in adults with arthritis: prevalence, mediators, and subgroups at greatest risk. Data from the 2007 National Health Interview Survey | Self-reported IA/OA diagnosis |
| Ma, 2024 | Non-linear association of sleep duration with osteoarthritis among U.S. middle-aged and older adults | Wrong patient population |
| Martinez, 1995 | Fibromyalgia versus rheumatoid arthritis: a longitudinal comparison of the quality of life | No full text |
| McBeth, 2022 | Sleep Disturbance and Quality of Life in Rheumatoid Arthritis: Prospective mhealth Study | Self-reported IA/OA diagnosis |
| McCurry, 2014 | Who benefits from CBT for insomnia in primary care? Important patient selection and trial design lessons from longitudinal results of the Lifestyles trial | Related to an included study |
| McCurry, 2019 | Telephone interventions for co-morbid insomnia and osteoarthritis pain: The OsteoArthritis and Therapy for Sleep (OATS) randomized trial design | Related to an included study |
| McCurry, 2020 | Perceptions of an education only control group in the osteoarthritis and therapy for sleep (OATS) study: an ongoing statewide, telephone-delivered CBT for insomnia (CBT-I) randomized trial | Wrong study design |
| McKenna, 2020 | The Impact of Exercise on Sleep in People with Rheumatoid Arthritis: a Pilot Randomised Controlled Trial | Wrong study design |
| McKenna, 2018 | The impact of exercise on sleep in people with rheumatoid arthritis: a pilot randomised controlled trial | Wrong study design |
| McManus, 2015 | Effect of an Arthritis Foundation Exercise Program on Sleep Quality/Sleep Disturbance in Seniors With Rheumatoid Arthritis: A Pilot Study | Diagnosis of IA/OA not clinically verified |
| Mease, 2019 | Translating Improvements with Ixekizumab in Clinical Trial Outcomes into Clinical Practice: ASAS40, Pain, Fatigue, and Sleep in Ankylosing Spondylitis | Related to an included study |
| Metin Ökmen, 2022 | Central sensitisation in primary Sjögren Syndrome and its effect on sleep quality | Wrong patient population |
| Miura, 2025 | Obstructive sleep Apnoea in patients with knee osteoarthritis before total knee arthroplasty and its impact on post-operative recovery of blood oxygen concentrations | Wrong outcomes |
| Modarresi, 2022 | Does the Association of Sleep Quality with Central Sensitization in People with Knee Osteoarthritis Differ Depending on How It’s Measured? A Comparison of Quantitative Sensory Testing and the Central Sensitization Inventory | No full text |
| Moldofsky, 1989 | Sleep influences on regional and diffuse pain syndromes associated with osteoarthritis | No full text |
| Moldofsky, 1983 | Alpha EEG sleep and morning symptoms in rheumatoid arthritis | No full text |
| Mulligan, 2015 | Sleep quality and nocturnal pain in patients with shoulder disorders | Wrong patient population |
| Mutoh, 2016 | Study on the frequency and risk factors of moderate-to-severe sleep apnea syndrome in rheumatoid arthritis | Wrong outcomes |
| NCT00530556 | Insomnia and Daytime Function in Osteoarthritis | Sleep not measured with a validated outcome measure |
| NCT00592449 | Sleep in Osteoarthritis Project | Related to an included study |
| NCT01683799 | Sleep, Pain and Inflammatory Processes in Older Adults With Osteoarthritis | Related to an included study |
| NCT00694447 | Acupuncture for Pain and Sleep Disturbance in Knee Osteoarthritis | Wrong study design |
| NCT00948610 | Sleep and Immunity in Rheumatoid Arthritis: remicade Substudy | Trial record not retrievable |
| NCT01142349 | Cognitive Behavioral Therapy for Arthritis Pain and Insomnia in Older Adults | Related to an included study |
| NCT01966835 | Effect of Moderate to High Intensity Aerobic Interval Training on Polysomnographic Measured Sleep in Patients With Rheumatoid Arthritis | Related to an included study |
| NCT02946957 | OsteoArthritis and Therapy for Sleep | Related to an included study |
| NCT03140995 | Sleep and Exercise in Rheumatoid Arthritis | Related to an included study |
| NCT03166462 | Obstructive Sleep Apnea in Elective Orthopaedic Surgery | Wrong outcomes |
| NCT03269760 | Multimodal Sleep Pathway for Shoulder Arthroplasty | Wrong patient population |
| NCT03441191 | Audio-visual Stimulation for Sleep Promotion in Older Adults With Osteoarthritis Pain | Diagnosis of IA/OA not clinically verified |
| NCT03766100 | Cognitive Behavioural Therapy for Insomnia on Sleep in Rheumatoid Arthritis | Related to an included study |
| NCT04549857 | Effects of Aromatherapy on Pain, Sleep Quality, and Psychological Distress in Patients With Rheumatoid Arthritis | Related to an included study |
| NCT05295004 | The Effect of Mindfulness Training Before Primary Total Joint Arthroplasty on Sleep Quality | Diagnosis of IA/OA not clinically verified |
| NCT05861934 | The Effect of Distant Reiki Application on Pain, Functional Status and Sleep Quality | Wrong patient population |
| NCT06912750 | 4-7-8 Breathing and Teach-Back Method for Pain, Anxiety, and Sleep After Knee Surgery: a Clinical Trial | Diagnosis of IA/OA not clinically verified |
| NCT06918847 | Comparative Effectiveness of Myofacial Release vs Cupping Therapy on Quality of Life in Restless Leg Syndrome With Knee O.A. | Wrong patient population |
| NCT03766100 | Cognitive behavioural therapy for insomnia in patients with rheumatoid arthritis: protocol for the randomised, single-blinded, parallel-group Sleep-RA trial | Related to an included study |
| NCT04549857 | The Effects of Aromatherapy on Pain, Sleep Quality, and Psychological Distress in Patients with Rheumatoid Arthritis | Related to an included study |
| NCT00367965 | The Effect of Eszopiclone 3 mg Compared to Placebo on Daytime Function in Subjects with Insomnia Related to Rheumatoid Arthritis | Related to an included study |
| NCT05888220 | The Effect Of Warm Salt Water and Warm Water Bath Applied to Hands and Feet of Patients With Rheumatoid Arthritis on Pain, Fatigue, Sleep Quality and Functional Capacity | Related to an included study |
| NCT05381129 | The Effect of Accompanying Temporomandibular Joint Dysfunction on Pain, Quality of Life, Sleep and Functionality in Patients with Chronic Migraine | Wrong patient population |
| NCT00592449 | Sleep Disturbance, Central Pain Modulation, and Clinical Pain in Osteoarthritis (The SOAP Study) | Related to an included study |
| NR | Mobile Motivational Physical Activity Targeted Intervention (mobmpati) to Improve Sleep in Older Adults with Osteoarthritis | Trial record not retrievable |
| NR | Knee arthritis and sleep disturbances: more reasons to get moving. | Trial record not retrievable |
| NR | Exploring Outcomes and Mechanisms of Acupuncture in Treating Pain and Sleep Disturbance in Knee Osteoarthritis | Trial record not retrievable |
| NCT02946957 | Efficacy of Scalable CBT for Insomnia in Older Adults with Osteoarthritis Pain | Related to an included study |
| NCT04948541 | Effect Of Sleep Apnea Treatment on Temporo-Mandibular Disorders in Patients Suffering from Sleep Apnea | Wrong patient population |
| NCT01966835 | Effect of Moderate to High Intensity Aerobic Interval Training on Polysomnographic Measured Sleep in Patients with Rheumatoid Arthritis. A Randomized Controlled Trial | Related to an included study |
| NCT03140995 | Does Exercise Have an Impact on Sleep and Mood in People Who Have Rheumatoid Arthritis: a Pilot Randomised Controlled Trial | Related to an included study |
| NCT01422967 | Changes Of Sleep on the Sensoriomotor and Cytokine in Patients with Knee Osteoarthritis | Related to an included study |
| NR | Assessment of Anxiety, Depression, Sleep Quality and Quality of Life in Rheumatoid Arthritis Patients | Related to an included study |
| NCT03545048 | A Randomized Controlled Trial Evaluating the Efficacy of Internet-Based Exercise Program Aimed at Treating Knee Osteoarthritis (ibeat-OA) | Related to an included study |
| NCT01832155 | Yoga for Managing Knee Osteoarthritis in Older Women: a Feasibility Study | Related to an included study |
| NCT01282372 | 2-year HRQL Observational Study Evaluating the Effect of Treatment with Adalimumab on Work Productivity and Sleep in Patients with Rheumatic Diseases in Greece | Related to an included study |
| *No author listed* | Cognitive behavioral therapy for sleep disorders in patients with rheumatoid arthritis | No full text |
| Neelapala, 2025 | Latent transition analysis of pain phenotypes in people at risk of knee osteoarthritis: The MOST cohort study | Wrong patient population |
| Nicassio, 2014 | Confirmatory factor analysis of the Pittsburgh Sleep Quality Index in rheumatoid arthritis patients | Wrong study design |
| Nicassio, 1992 | Longitudinal relationships among pain, sleep problems, and depression in rheumatoid arthritis | Sleep not measured with a validated outcome measure |
| Ni, 2025 | Prevalence of complementary therapy use in patients with inflammatory arthritis: a cross-sectional study in China | Topic not alligned with the review scope |
| Ni, 2024 | Sleep patterns, physical activity, genetic susceptibility, and incident rheumatoid arthritis: a prospective cohort study | Wrong patient population |
| N., 2023 | Sleep Quality in Patients with Rheumatoid Arthritis: a Single Center Experience from Jodhpur, India | Wrong study design |
| NL-OMON52175 | Chronotherapy in Inflammatory Arthritis (ChronIA trial): a randomized controlled trial comparing the effectiveness of morning and evening dosing of tofacitinib extended-release | Topic not alligned with the review scope |
| Oyama, 1995 | [Sleep apnea syndrome in rheumatoid arthritis (RA) patients complicated with cervical and temporomandibular lesions] | No full text |
| Pacheco-Parés, 2021 | Physical Activity, Sedentary Time, And Sleep Quality Among Hispanic Women With And Without Rheumatoid Arthritis...2021 ACSM Annual Meeting & World Congresses [Virtual], June 1-5, 2021 | Wrong study design |
| PACTR20240648706305 | Compared effects of aerobic and resistance and combined trainings on sleep quality and fatigue in women with rheumatoid arthritis | Trial record not retrievable |
| Palominos, 2020 | Determinants of sleep impairment in psoriatic arthritis: An observational study with 396 patients from 14 countries | Sleep not measured with a validated outcome measure |
| Park, 2019 | Relationship between Sleep Duration and Osteoarthritis in Middle-Aged and Older Women: A Nationwide Population-Based Study | Sleep not measured with a validated outcome measure |
| Pépin, 1995 | Sleep apnoea syndrome secondary to rheumatoid arthritis | Wrong study design |
| Pope, 2012 | Rapid reductions in fatigue and sleep problems and correlation with improvements in patient-reported outcomes in patients with active rheumatoid arthritis treated with certolizumab pegol in the realistic 12-week phase iiib randomized controlled study | Wrong study design |
| Purabdollah, 2015 | Relationship between Sleep Disorders, Pain and Quality of Life in Patients with Rheumatoid Arthritis. | Related to an included study |
| Ran, 2021 | The multimorbidity of hypertension and osteoarthritis and relation with sleep quality and hyperlipemia/hyperglycemia in China's rural population | Wrong patient population |
| Raymond, 2024 | A concept elicitation study to understand the relationship between sleep and pain in rheumatoid arthritis and axial spondyloarthritis | Sleep not measured with a validated outcome measure |
| Reading, 2009 | Do rheumatoid arthritis patients have a higher risk for sleep apnea? | Wrong outcomes |
| Rodríguez De La Serna, 2004 | Comparison between a group of patients with fibromyalgia with another group suffering from rheumatoid arthritis to assess their psychopathology and sleep disorders | No full text |
| Roehrs, 2013 | Nocturnal sleep, daytime sleepiness and fatigue in fibromyalgia patients compared to rheumatoid arthritis patients and healthy controls: a preliminary study | Related to an included study |
| Sasahara, 2023 | Sleep Disturbance In Elderlies Is Associated With Knee Pain With Knee Osteoarthritis -The Bunkyo Health Study | Wrong study design |
| Schell, 2015 | Restless legs syndrome in psoriasis: an unexpected comorbidity | Wrong patient population |
| Seçkin, 2000 | The psychiatric symptoms and sleep disorders in patients with chronic rheumatic pain: The comparative evaluation of patients with rheumatoid arthritis, ankylosing spondylitis, fibromyalgia and degenerative osteoarthritis | Sleep not measured with a validated outcome measure |
| Sengupta, 2020 | Certolizumab pegol-treated patients with non-radiographic axspa demonstrate improvements in sleep quality and other patient reported outcome | Wrong study design |
| Sezgin, 2017 | Comparing the effects of physical therapy and non-steroidal anti-inflammatory treatment on sleep quality, quality of life and clinical status in knee osteoarthritis | Wrong study design |
| Sharma, 2022 | Effect of Hand Reflexology Massage On Pain, Fatigue And Sleep Deprivation In Patients Suffering From Rheumatoid Arthritis | Diagnosis of IA/OA not clinically verified |
| Sha, 2025 | Sleep patterns, genetic susceptibility, and osteoarthritis risk: insights from the UK biobank and external validation in the xiangya osteoarthritis study | Wrong patient population |
| Shen, 2016 | Risk of obstructive sleep apnoea in patients with rheumatoid arthritis: a nationwide population-based retrospective cohort study | Wrong outcomes |
| Shoda, 2009 | Sleep apnea in rheumatoid arthritis patients with occipitocervical lesions: the prevalence and associated radiographic features | Wrong outcomes |
| Shodiev, 2023 | Clinical and Biochemical Changes and Their Correction in Patients with Metabolic Phenotype of Osteoarthritis and Insomnia | Sleep not measured with a validated outcome measure |
| Silva, 2020 | Depression, disability and sleep disturbance are the main explanatory factors of fatigue in rheumatoid arthritis: a path analysis model | Sleep not measured with a validated outcome measure |
| Song, 2018 | Reallocating time spent in sleep, sedentary behavior and physical activity and its association with pain: a pilot sleep study from the Osteoarthritis Initiative | Wrong patient population |
| Song, 2021 | Sleep Disturbance Trajectories in Osteoarthritis | Sleep not measured with a validated outcome measure |
| Stanciu, 2022 | Associations of rheumatoid arthritis and rheumatoid factor with mental health, sleep and cognition characteristics in the UK Biobank | Self-reported IA/OA diagnosis |
| Stebbings, 2010 | A comparison of fatigue correlates in rheumatoid arthritis and osteoarthritis: disparity in associations with disability, anxiety and sleep disturbance | Sleep not measured with a validated outcome measure |
| Stebbing, 2014 | Yoga for Pain and Sleep Quality in Rheumatoid Arthritis: A Pilot Randomized Controlled Trial. | Wrong study design |
| Strand, 2014 | Impact of sarilumab on health related quality of life (HRQoL), fatigue, and sleep in rheumatoid arthritis patients at week 24-results of a phase 3, randomized, double-blind, placebo-controlled, multi-center study | Wrong study design |
| Tanfield, 1978 | Ankylosing spondylitis and sleep | Wrong study design |
| Tang, 2016 | Fatigue, dysfunctional beliefs about sleep, and pain catastrophizing are predictors of both sleep complaint and daytime function in a large cohort of older adults with co-morbid insomnia and osteoarthritis pain | No full text |
| Tang, 2018 | Open-loop audio-visual stimulation for insomnia in older adults with osteoarthritis pain | No full text |
| Temel, 2016 | Prevalence of restless legs syndrome among psoriasis patients and association with depression and sleep quality | Wrong patient population |
| Thewlis, 2019 | Objectively measured 24-hour activity profiles before and after total hip arthroplasty | Wrong patient population |
| Toledano, 2024 | Effect of a Mindfulness-Based Intervention on the Health and Well-Being of Patients with Psoriatic Arthritis (The Oniros Study) | Wrong study design |
| Treharne, 2007 | Sleep disruption frequency in rheumatoid arthritis: perceived stress predicts poor outcome over one year | Sleep not measured with a validated outcome measure |
| UMIN000014624 | Effects of postoperative administration of celecoxib on postoperative pain management in patients after total knee arthroplasty. A randomized controlled study | Wrong patient population |
| UMIN000018816 | Study on the relationship between arthritis and sleep disturbance | Sleep not measured with a validated outcome measure |
| UMIN000039116 | Effects of Sleep Management on Postoperative Pain in patients after total knee arthroplasty. A randomized controlled study | Wrong patient population |
| Valentino, 2023 | The impact of sleep apnea on primary reverse shoulder arthroplasty for the treatment of glenohumeral osteoarthritis | Wrong outcomes |
| Vasanthakumar, 1987 | The effects of differing pharmaceutical preparations of indomethacin on night pain and morning stiffness in patients with rheumatoid arthritis | Sleep not measured with a validated outcome measure |
| Vitiello, 2013 | Cognitive-behavioral treatment for comorbid insomnia and osteoarthritis pain in primary care: the lifestyles randomized controlled trial | Related to an included study |
| Vorsanger, 2007 | Post hoc analysis of a randomized, double-blind, placebo-controlled efficacy and tolerability study of tramadol extended release for the treatment of osteoarthritis pain in geriatric patients | Sleep not measured with a validated outcome measure |
| Wali, 2019 | Prevalence of obstructive sleep apnea in patients with rheumatoid arthritis | Wrong study design |
| Wali, 2020 | Prevalence of obstructive sleep apnea in patients with rheumatoid arthritis | Wrong outcomes |
| Walsh, 1996 | Effects of triazolam on sleep, daytime sleepiness, and morning stiffness in patients with rheumatoid arthritis | No full text |
| Wang 2024 | Effects of serum inflammatory factors, health index and disease activity scores on ankylosing spondylitis patients with sleep disorder | Sleep not measured with a validated outcome measure |
| Wang, 2023 | Association of Sleep Disturbance With Catastrophizing and Knee Pain: Data From the Osteoarthritis Initiative | Sleep not measured with a validated outcome measure |
| Wegener, 1988 | Treatment of sleep disturbance in rheumatoid arthritis: A single-subject experiment | Wrong study design |
| Wells, 2007 | Determining the minimal clinically important differences in activity, fatigue, and sleep quality in patients with rheumatoid arthritis | Related to an included article |
| Wells, 2008 | Responsiveness of patient reported outcomes including fatigue, sleep quality, activity limitation, and quality of life following treatment with abatacept for rheumatoid arthritis | Related to an included article |
| Weman, 2024 | Intense symptoms of pain are associated with poor sleep, fibromyalgia, depression and sleep apnea in patients with rheumatoid arthritis and psoriatic arthritis. A register-based study | Sleep not measured with a validated outcome measure |
| Whibley, 2022 | Sleep Efficiency Moderates the Next-day Association between Physical Activity and Pain Intensity among Adults with Knee Osteoarthritis | Wrong study design |
| Whibley, 2022 | Development of a hybrid sleep and physical activity improvement intervention for adults with osteoarthritis-related pain and sleep disturbance: a focus group study with potential users | Sleep not measured with a validated outcome measure |
| Wilcox, 2000 | Factors related to sleep disturbance in older adults experiencing knee pain or knee pain with radiographic evidence of knee osteoarthritis | Sleep not measured with a validated outcome measure |
| Winter, 2023 | Prevalence of Sleep-Related Breathing Disorders in Patients with Rheumatoid Arthritis, Psoriatic Arthritis, and Peripheral Spondyloarthritis | Wrong study design |
| Wong, 2011 | Increased Prevalence of Sleep Disorders and Medical Comorbidities in Psoriatic Arthritis Patients Compared with Patients with and without Psoriasis | Wrong patient population |
| Wu, 2022 | Needle-Warming Moxibustion plus Multirehabilitation Training to Improve Quality of Life and Functional Mobility of Patients with Rheumatoid Arthritis after Medication | Retracted article |
| Xu, 2015 | [An investigation of sleep disturbance and related factors in rheumatoid arthritis patients] | No full text |
| Zamarrón, 2004 | Worsening of obstructive sleep apnoeas in a patient with rheumatoid arthritis treated with anti-tumor necrosis factor | Wrong study design |
| Zaslavsky, 2019 | Use of a Wearable Technology and Motivational Interviews to Improve Sleep in Older Adults With Osteoarthritis and Sleep Disturbance: A Pilot Study | Diagnosis of IA/OA not clinically verified |
| Zhang, 2013 | [Relationship between sleep quality and nocturnal pain in ankylosing spondylitis] | No full text |
| Zhang, 2024 | Effects of auricular acupressure on sleep and pain in elderly people who have osteoarthritis and live in nursing homes: a randomized, single-blind, placebo-controlled trial | Wrong study design |
| Zhou 2024 | Association between nighttime sleep duration and quality with knee osteoarthritis in middle-aged and older Chinese: A longitudinal cohort study | Self-reported IA/OA diagnosis |
