# Supplementary material for: Sleep measurement and clinical impact in osteoarthritis and inflammatory arthritis: a scoping review
Source: Rheumatology (Oxford). 2026 Aug 11;65(8):keag412. doi: 10.1093/rheumatology/keag412 (PMC13523860; doi:10.1093/rheumatology/keag412)
Supplement: Supplementary file 1 [file keag412_supplementary_data.zip › Supplement_Data.zip!/rhe-25-3089-File009.docx]

**Supplementary Table S2**

**Characteristics of the included published studies**

| **Author, year** | **Country** | **Population** | | **Sample size** | | **Sex/Gender distinction reported (Yes/No)** | **Sex** | | **Notes on Sex/Gender** | **Age (years)** | |
| --- | --- | --- | --- | --- | --- | --- | --- | --- | --- | --- | --- |
|  |  | I | C | I | C |  | Male | Female |  | I | C |
| ***Cross-sectional studies*** | | | | | | | | | | | |
| Adawi, 2020 | Israel | RA | NA | 18 | NA | Yes | 2 | 16 | Sex and gender terms used interchangeably | 57.6 ± 13.6 | NA |
| Albayrak Gezer, 2017 | Turkey | RA | NA | Group 1= 52 Group 2= 84 | NA | Yes | 25 | 111 | NA | Group 1: 67.7 ± 4.3  Group 2: 45.7 ± 10.5 | NA |
| Anjum, 2022 | Pakistan | RA | NA | 185 | NA | Yes | 73 | 112 | Gender used as a synonym for sex | 44.3 ± 9.3 | NA |
| Austad, 2017 | Norway | RA | NA | 986 | NA | Yes | 228 | 758 | Gender used as a synonym for sex | 59.4 ± 12.5 | NA |
| Aydemir, 2024 | USA | RA | NA | 48 | NA | Yes | 4.2% | 95.8% | NA | 53.4 ± 14.7 | NA |
| Azizli, 2023 | Turkey | RA | HC | 34 | 30 | Yes | 20 | 44 | NA | 72.8 ± 6.7 | 70.5 ± 4.4 |
| Azzam, 2024 | Egypt | RA | NA | 300 | NA | Yes | 24 | 276 | NA | 38.5 ± 8.8 | NA |
| Bagnato, 2016 | Italy | RA  SSc | HC | RA: 34 SSc: 33 | 34 | No | 0 | 100 | Only no. of females reported, without specifying whether this refers to sex or gender | 52 ± 12.6 (RA)  56.1 ± 11.5 (SSc) | 53.2 ± 12.7 |
| Baldelli, 2019 | Italy | RA  Seronegative SpA | NA | RA: 64 SpA: 39 | NA | Yes | 36 | 67 | Gender used as a synonym for sex | 53.7 ± 14.6 | NA |
| Belt, 2009 | Finland | RA  FM | GP | RA: 31 FM: 37 | 1284 | Yes | 566 | 786 | Gender used as a synonym for sex | RA: 52.1 ± 8.0 FM: 45.7 ± 8.2 | 49.8 (SD: NR) |
| Bilge, 2017 | Turkey | RA | NA | 152 | NA | Yes | 28 | 124 | NA | 53.0 ± 11.4 | NA |
| Bjurström, 2017 | USA | RA | HC | 24 | 48 | Yes | 12.5 % (RA) 18.7 % (HC) | 87.5 % (RA) 81.3 % (HC) | Gender used as a synonym for sex | 60.4 ± 11.5 | 61.8 ± 10.2 |
| Borman, 1999 | Turkey | RA  FM | HC | RA: 26 FM: 22 | 25 | Yes | NA | 73 | NA | RA: 44.5 ± 8.03  FM: 38.09 ± 6.8 | 41.1± 9.2 |
| Brahem, 2024 | Tunisia | RA | NA | 100 | NA | Yes | 11 | 89 | Sex and gender terms used interchangeably | 53.2 ±11.2 | NA |
| Chalupczak, 2025 | USA | RA | NA | 48 | NA | Yes | 4.2% | 95.8% | Only no. of females reported, without specifying whether this refers to sex or gender | Median 55.0 (41.5–64.8) | NA |
| Chau, 2023 | China | RA | NA | 164 | NA | Yes | 20 | 144 | NA | 58.3 ± 14.0 | NA |
| Clarke, 2013 | UK | Active RA | NA | 10 | NA | No | 3 | 7 | Only no. of females/males reported, without specifying whether this refers to sex or gender | 64.7 ± 10.0 | NA |
| Demir, 2021 | Turkey | RA | HC | 72 | 72 | Yes | 28 | 116 | Gender used as a synonym for sex | 51.9 ± 11.4 | 49.5 ± 11.2 |
| Deniz, 2023 | Turkey | RA | HC | 50 | 50 | Yes | 36 | 64 | Gender used as a synonym for sex | Median 70 (65–86) | 73 (65–86) |
| Fouda, 2014 | Egypt | RA | NA | 30 | NA | No | 0 | 30 | Only no. of females reported, without specifying whether this refers to sex or gender | 40.3 ± 6.2 | NA |
| Gamal, 2020 | Egypt | RA | HC | 58 | 30 | Yes | 6 | 82 | NA | 44.1 ± 11.8 | 41.4 ± 10.9 |
| Gjevre, 2012 | Canada | RA with abonormal ESS scores | RA with normal ESS scores | 10 | 15 | Yes | 7 | 18 | NA | 58.2 ± 7.6 | 61.8 ± 11.0 |
| Goes, 2017 | Brazil | RA | NA | 112 | NA | No | 19 | 93 | Sex and gender terms used interchangeably | 55.4 ± 10.9 | NA |
| Goodchild, 2010 | UK | RA | pSS | 25 | 14 | Yes | NA | 39 | Gender used as a synonym for sex | 61.9 ± 10.6 | 58.1 ± 9.3 |
| Gouda, 2023 | Egypt | RA | HC | 247 | 60 | Yes | 67 | 240 | NA | 45.5 ± 8.2 | 44.0 ± 8.7 |
| Grabovac, 2018 | Austria | RA | NA | 95 | NA | Yes | 31 | 64 | NA | 50.6 ± 9.6 | NA |
| Guo, 2016 | China | RA | HC | 131 | 104 | Yes | 35 | 200 | NA | 54.7 ± 11.5 | 54.8 ± 11.2 |
| Hamdi, 2021 | Tunisia | RA | OA | 70 | 40 | Yes | 28 | 82 | NA | 51.9 ± 12.2 | 57.5 ± 12.9 |
| Hammam, 2020 | Egypt | RA | HC | 115 | 46 | Yes | 12 | 149 | NA | 45.5 ± 11.9 | 42.4 ± 9.9 |
| Hirsch, 1994 | Belgium | RA | HC | 19 | 19 | Yes | RA: 1 | RA: 18 | NR for HC | 47.6 ± 12.1 | 41.9 ± 8.2 |
| Hughes, 2021 | UK | RA | NA | 200 | NA | Yes | 53 | 147 | NA | 61.8 ± 13.9 | NA |
| Juárez-Rojop, 2023 | Mexico | RA | NA | 102 | NA | Yes | 7 | 95 | NA | 52.0 ± 12.6 | NA |
| Katz, 2016 | USA | RA | NA | 158 | NA | Yes | 24 | 134 | NA | 59.2 ± 11.3 | NA |
| Katz, 2023 | USA | RA | NA | 4200 | NA | Yes | 983 | 3517 | NA | 64.6 ± 12.0 | NA |
| Kayahan Satış, 2025 | Turkey | RA | NA | 77 | NA | Yes | 31 | 46 | NA | 70.8 ± 4.9 | NA |
| Kontodimopoulos, 2020 | Greece | RA | NA | 147 | NA | Yes | 32 | 115 | NA | 63.7 ± 10.9 | NA |
| Kruthik, 2025 | India | RA | NA | 98 | NA | Yes | 13 | 85 | NA | 52.1 ± 9.3 | NA |
| Kumar, 2022 | India | RA | NA | 110 | NA | Yes | 18 | 92 | NA | 50.2 ± 12.9 | NA |
| Lee, 2009 | USA | RA | NA | 59 | 0 | Yes | 0 | 59 | NA | 61.0 ± 15.2 | NA |
| Lee, 2013 | USA | RA | HC | 58 | 54 | Yes | 0 | 112 | NA | 60.9 ± 9.7 | 62.5 ± 9.3 |
| Lee, 2022 | South Korea | RA | NA | 100 | NA | Yes | 19 | 81 | NA | 55.6 ± 11.1 | NA |
| Liu, 2022 | China | RA | NA | 141 | 0 | Yes | 22 | 119 | NA | 56.9 ± 13.1 | NA |
| Lopes, 2021 | Brazil | RA | NA | 110 | NA | Yes | 0 | 110 | NA | 51.1 ± 13.0 | NA |
| Løppenthin, Esbensen, Jennum, 2015 | Denmark | RA | NA | 384 | NA | Yes | 75 | 309 | NA | 59 (range 22–88) | NA |
| Løppenthin, Esbensen, Østergaard, 2015 | Denmark | RA | NA | 443 | NA | Yes | 87 | 356 | NA | 60 (range 21–88) | NA |
| Luyster, 2011 | USA | RA | NA | 162 | NA | Yes | 39 | 123 | NA | 58.5 ± 10.2 | NA |
| Mahowald, 1989 | USA | RA | NA | 16 | NA | Yes | 14 | 2 | NA | 61.7 ± 4.4 | NA |
| McKenna, 2018 | Ireland | RA | NA | 79 | NA | Yes | 27 | 48 | NA | ≤49y: 20% 50–59y: 20%  60–69y: 40%  70–79y: 20% | NA |
| Mehr, 2023 | Iran | RA | NA | 202 | NA | Yes | 121 | 81 | NA | 51.0 ± 4.3 | NA |
| Minnock, 2015 | Ireland | RA | RA | 28 | 28 | Yes | 28 | 28 | NA | 58 ± 11 | 58 ± 11 |
| Miyauchi, 2021 | Japan | Sjögren’s syndrome | RA | 25 | 17 | Yes | 0 | 35 | NA | 56.8 ± 1.8 | 59.1 ± 2.8 |
| Mustafa, 2019 | Saudi Arabia | RA | NA | 101 | NA | Yes | 5 | 96 | NA | 48.7 ± 14.6 | NA |
| Nicassio, 2012 | USA | RA | NA | 106 | NA | Yes | 16 | 90 | NA | 56.2 ± 12.5 | NA |
| Pehlivan, 2016 | Turkey | RA | RA | 99 | 83 | Yes | I: 19  C: 15 | I: 80  C: 68 | NA | ≥65 | <65 |
| Petzinna , 2024 | Germany | RA  PsA | HC | RA: 22 PsA: 17 | 23 | Yes | RA: 16  PsA: 7 HC: 14 | RA: 6  PsA: 10  HC: 9 | NA | RA: 56.4 (21.7–81.9) PsA: 44.9 (20.2–67.0) | HC: 50.8 (25.3–67.5) |
| Punshi, 2023 | Pakistan | RA | NA | 143 | NA | Yes | 47 | 96 | NA | 51.2 ± 9.6 | NA |
| Purabdollah, 2017 | Iran | RA | NA | 210 | NA | Yes | 53 | 157 | NA | 48.4 ± 12.9 | NA |
| Radwan, 2021 | Egypt | RA | HC | 133 | 76 | Yes | RA: 8  HC: 6 | RA: 125  HC: 70 | NA | 42.5 ± 9.5 | 42.8 ± 9.1 |
| Rezazadeh, 2024 | Iran | RA | NA | 137 | NA | Yes | 19 | 118 | NA | 45 ± 23 | NA |
| Shen, 2021 | China | RA | NA | 580 | NA | Yes | 67 | 513 | Gender used as a synonym for sex | 51.04 ± 24.65 | NA |
| Solak, Dündar, 2009 | Turkey | RA | HC | 55 | 20 | Yes | 18 | 57 | NA | 51.8 ± 12.1 | 45.7 ± 14.0 |
| Son, 2015 | South Korea | RA | HC | 130 | 67 | Yes | 39 | 158 | Gender used as a synonym for sex | Median 53.4 (19-80) | 49.2 (26-79) |
| Song, 2023 | USA | RA | NA | 227 | NA | Yes | 43 | 184 | NA | 55.0 ± 14.1 | NA |
| Szady, 2017 | Poland | RA | NA | 38 | NA | Yes | 12 | 26 | Gender used as a synonym for sex | 56.3 ± 9.9 | NA |
| Tabaraii, 2021 | Iran | RA | NA | 142 | NA | Yes | 26 | 116 | NA | 52.3± 13 | NA |
| Tamm, 2021 | Sweden | RA Allergy | RA  Allergy | RA: 18 Allergy: 18 | RA: 19 Allergy: 13 | Yes | 24 | 44 | NA | RA: Median 53 (48.5–58.5) Allergy: 34 (30.2–44.2) | RA: 49 (45.5–54.0) Allergy: 34 (27.0–46.0) |
| Taylor-Gjevre, 2011a | Canada | RA OA | NA | RA: 145 OA: 78 | NA | Yes | RA: 32 OA: 25 | RA: 113 OA: 53 | Gender used as a synonym for sex | RA: 54.2 ± 15.7 OA: 59.9 ± 13.1 | NA |
| Tuncay, 2024 | Turkey | RA | HC | 78 | 48 | Yes | 22 | 104 | Gender used as a synonym for sex | 48.1 ± 11.0 | 45.7 ± 7.0 |
| Ulus, 2011 | Turkey | FM RA | HC | FM: 40 RA: 40 | 40 | Yes | 0 | 100 | NA | FM: 43.0 ± 10.3  RA: 43.8 ± 11.2 | 42.1 ± 11.0 |
| Waheed, 2024 | Pakistan | RA | NA | 250 | NA | Yes | 90 | 160 | Gender used as a synonym for sex | 41.9 ± 7.9 | NA |
| Westhovens, 2014 | Belgium | RA | NA | 305 | NA | Yes | 87 | 218 | NA | 57 ± 12.4 | NA |
| Wolfe, 2006 | USA | RA | Non-inflammatory disorders (OA or back pain problems) | 8676 | 1364 | Yes | 1961 | 6715 | Gender used as a synonym for sex | 61.1 ± 12.6 | NA |
| Bilge, 2016 | Turkey | RA | DM | 39 | 40 | Yes | 22 | 57 | Gender used as a synonym for sex | 49.0 ± 13.5 | 47.9 ± 9.7 |
| Yaseen, 2024 | Pakistan | RA | NA | 385 | NA | Yes | 93 | 292 | Gender used as a synonym for sex | 18–30y: 7.8%  31–40y: 14.3%  41–50y: 23.4%  51–60y: 26.0%  >60y: 28.6% | NA |
| Hörnberg , 2021 | Sweden | Early RA | NA | 78 | NA | Yes | 24 | 54 | NA | 55.4 ±14.4 | |
| Feehan, 2024 | Canada | RA | NA | 203 | NA | Yes | 17 | 186 | NA | 56.2 ± 13.0 | NA |
| Fawzy, 2022 | Egypt | RA KOA | HC | RA: 20 KOA: 20 | 20 | Yes | 15 | 45 | NA | RA: 41.7 ± 6.3 KOA: 43.2 ± 6.5 | 39.9 ± 4.3 |
| Akintayo, 2019 | Nigeria | KOA | NA | 250 | NA | Yes | 10 | 240 | Gender used as a synonym for sex | 59.9 ±10.6 | NA |
| Allen, 2008 | USA | Symptomatic hip or knee OA | Adults without symptomatic hip or knee OA | 759 | 1923 | Yes | 34.3% | 65.7% | NA | 45-54y: 16.2;  55-64y: 32.0%; ≥65y: 51.8% | 45-54y: 27.5;  55-64y: 32.9%; ≥65y: 39.6% |
| Chen, 2014 | Taiwan | OA | NA | 192 | NA | Yes | 54 | 138 | Gender used as a synonym for sex | Median 70 (42-89) | NA |
| Fertelli, 2019 | Turkey | KOA | HC | 151 | 157 | Yes | KOA: 15.2% HC: 8.2% | KOA: 84.8% HC: 91.8% | Gender used as a synonym for sex | 53.9 ± 6.8 | 52.9 ± 8.0 |
| Kakazu, 2024 | Brazil | OA | HC | 160 | 291 | Yes | 146 | 306 | NA | 71.0 ± 7.8 | 72.3 ± 7.9 |
| Kuralay, 2018 | Turkey | OA | NA | 90 | NA | Yes | 22 | 68 | NA | 62.3 ± 12.9 | NA |
| Lee, 2019 | South Korea | OA | NA | 222 | NA | Yes | 105 | 117 | NA | 75.6 ± 5.5 | NA |
| Lošťák, 2022 | Czech Republic | OA | OA | 27 | 59 | Yes | I: 17 C: 34 | I: 10  C: 25 | NA | 59.0 ± 4.9 | 72.4 ± 5.5 |
| Martinez, 2019 | USA | OA | NA | 106 | NA | Yes | 43 | 63 | NA | 62.7 ± 11.8 | NA |
| Mesci , 2015 | Turkey | OA | NA | Group 1: 27  Group 2: 28 | NA | Yes | 22 | 33 | NA | Group 1: 69.8 ± 4.6  Group 2: 72.7 ± 5.9 | NA |
| Murphy, 2011 | USA | OA | NA | 55 | NA | Yes | 0 | 55 | NA | 62.6 ± 7.5 | NA |
| Parmelee, 2017 | USA | OA | OA | 96 | 128 | Yes | I: 15  C: 36 | I: 81  C: 92 | NA | 63.2 ± 8.6 | 64.2 ± 9.1 |
| Petrov, 2015 | USA | OA | OA | 88 | 52 | Yes | 68 | 72 | NA | 55.1 ± 7.1 | 58.1 ± 7.3 |
| Sasaki, 2014 | Japan | OA | NA | 1214 | NA | Yes | 456 | 758 | NA | 58.1 ± 13.0 | NA |
| Spira, 2015 | USA | OA  OA + Insomnia | HC Only Insomnia | OA: 28 OA + Insomnia: 114 | HC: 28 Only Insomnia: 30 | Yes | 58 | 142 | Gender used as a synonym for sex | OA: 66.9 ± 10.2 OA + Insomnia: 59.4 ± 9.0 | HC: 57.6 ± 7.4 Only Insomnia: 58.9 ± 8.6 |
| Stocks, 2018 | UK | OA | NA | 423 | NA | Yes | 173 | 250 | NA | 69.7 ± 9.3 | NA |
| Taylor, 2018 | USA | OA | NA | 300 | NA | Yes | 273 | 27 | Gender used as a synonym for sex | 61.1 ± 9.2 | NA |
| Thorlund, 2025 | Denmark | OA | NA | 8162 | NA | Yes | 2736 | 5426 | Gender used as a synonym for sex | 66.7 ± 9.6 | NA |
| Yeung, 2018 | UK | FM OA | HC | FM: 19 OA: 17 | 10 | Yes | 0 | 46 | NA | FM: 40.7 ± 11.5 OA: 46.5 ± 11.6 | 38.4 ± 13.8 |
| Yilmaz, 2021 | Turkey | OA | NA | 151 | NA | Yes | 24 | 127 | Gender used as a synonym for sex | 60.1 ± 9.0 | NA |
| Aydin, 2015 | Turkey | AS | HC | 55 | 55 | Yes | 95 | 15 | Gender used as a synonym for sex | 43 ± 1 | 42 ± 9 |
| Demirci, 2016 | Turkey | AS | HC | AS with RLS: 39 AS without RLS: 69 | 64 | Yes | 114 | 58 | Sex and gender terms used interchangeably | Median 41.0 (32.0–50.0) | 37.0 (26.0–50.0) |
| Aykurt Karlıbel, 2023 | Turkey | axSpA | HC | 82 | 40 | Yes | 94 | 28 | Gender used as a synonym for sex | 38.8 ± 10.1 | 38.6 ± 7.5 |
| Cai, 2025 | China | axSpA | HC | Active: 45 inactive (remission): 49 | 94 | Yes | 96 | 92 | Sex and gender terms used interchangeably | Active: 44.3 ± 13.8 inactive: 45.4 ± 13.8 | 43.9 ± 12.8 |
| Cano-García, 2021 | Spain | axSpA PsA | NA | axSpA: 152 PsA: 149 | NA | Yes | 155 | 146 | NA | axSpA: 49.4 ± 10.4 PsA: 48.4 ± 10.1 | NA |
| Da Costa, 2009 | Canada | SpA | NA | 125 | NA | Yes | 58 | 67 | Gender used as a synonym for sex | 46.5 ± 12.5 | NA |
| Bedaiwi, 2021 | Saudi Arabia | SpA (AS, PsA, undifferentiated SpA, IBD-related arthropathy) | NA | 79 | NA | Yes | 39 | 40 | Gender used as a synonym for sex | 41.1 ± 11.8 | NA |
| Fongen, 2024 | Norway | axSpA | NA | 99 | NA | Yes | 47 | 52 | NA | 46.1 | NA |
| Frede, 2023 | Germany | axSpA PsA | NA | axSpA: 162 PsA: 168 | NA | Yes | 183 | 147 | NA | 53.3 ± 14.0 | NA |
| Ghani Sayed, 2025 | Egypt | axSpA | NA | 40 | NA | Yes | 20 | 20 | NA | 18-40y: 24 (60%)  >40y: 16 (40%) | NA |
| Günaydin , 2009 | Turkey | axSpA | NA | 62 | NA | Yes | 52 | 10 | NA | 39.6 ± 10.3 | NA |
| Gezer, 2017 | Turkey | PsA | HC | 41 | 38 | Yes | 50 | 29 | Sex and gender terms used interchangeably | 41.4 ± 12.6 | 38.0 ± 9.8 |
| Hultgren, 2000 | Sweden | axSpA | HC | 70 | 3558 | Yes | 1822 | 1806 | NA | 45.7 ± 7.7 | 46.6 ± 10.6 |
| İn, 2015 | Turkey | axSpA on Anti-TNF | AxSpa on NSAIDs | 28 | 31 | Yes | 46 | 13 | NA | 38.9 ± 8.3 | 39.2 ± 9.3 |
| İn, 2016 | Turkey | axSpA | NA | 42 | NA | Yes | 36 | 6 | NA | 39.4 ± 9.1 | NA |
| Jiang, 2018 | China | axSpA | HC | 683 | 697 | Yes | 1086 | 294 | NA | 27.3 ± 8.7 | 27.5 ± 7.7 |
| Karadağ, 2012 | Turkey | axSpA | HC | 171 | 86 | Yes | 111 | 146 | NA | 36.4 ± 11.1 | 38.4 ± 9.6 |
| Mansoor, 2024 | Pakistan | axSpA | NA | 100 | NA | Yes | 68 | 32 | NA | 37.5 ± 10.3 | NA |
| Nie, 2018 | China | axSpA | NA | 281 | NA | Yes | 191 | 90 | NA | 31.7 ± 9.8 | NA |
| Ozkan, 2016 | Turkey | AS | AS | 25 | 21 | Yes | I: 17  C: 14 | I: 8  C: 7 | NA | 29.8 ± 6.5 | 25.9 ± 5.7 |
| Skougaard, 2023 | Denmark | PsA | Psoriasis HC | 109 | Psoriasis: 20 HC: 20 | Yes | 64 | 85 | Gender used as a synonym for sex | Median 53.9 (45.5–62.2) | Psoriasis: 52.0 (41.6–62.5) HC: 51.3 (45.1–62.5) |
| Solak, Fidan, 2009 | Turkey | AS | NA | 31 | NA | Yes | 31 | No | NA | 35.5 ± 10.6 | NA |
| Song, 2017 | China | AS | NA | 300 | NA | Yes | 198 | 97 | Gender used as a synonym for sex | 31.7 ± 9.7 | NA |
| Song, 2021 | South Korea | AS | NA | 107 | NA | Yes | 88 | 19 | NA | 35.8 ± 11.8 | NA |
| Urkmez, 2020 | Turkey | AS | HC | 150 | 80 | Yes | 142 | 88 | Gender used as a synonym for sex | 38.4 ± 9.1 | 36.2 ± 9.9 |
| Wadeley, 2018 | UK | AS | NA | 659 | NA | Yes | 500 | 159 | Gender used as a synonym for sex | Median 50  Interquartile 39–61 | NA |
| Toledano, 2023 | Spain | PsA | NA | 247 | NA | Yes | 137 | 110 | NA | 52.4 ± 11.7 | NA |
| Toledano, 2024 | Spain | PsA | NA | 203 | NA | Yes | 108 | 95 | Gender used as a synonym for sex | 54.6 ± 11.3 | NA |
| Wong, 2017 | Canada | PsA | Psoriasis HC | 113 | Psoriasis: 62 HC: 52 | Yes | 102 | 125 | NA | 57.4 ± 11.6 | Psoriasis: 56.9 ± 14.2 HC: 42.2 ± 13.6 |
| Krajewska-Włodarczyk, 2018 | Poland | PsA | HC | 62 | 41 | Yes | 54 | 49 | NA | 47.8 ± 8.9 | 42.4 ± 11.6 |
| Ying, 2025 | China | AS | NA | 205 | NA | Yes | 138 | 67 | Gender used as a synonym for sex | 33.6 ± 11.2 | NA |
| Zúñiga-Vera, 2023 | Ecuador | SpA | NA | 72 | NA | No | 12 | 60 | Only no. of women reported, without specifying whether this refers to sex or gender | Median 42 (33.8-56.3) | NA |
| Jiang, 2015 | China | AS | NA | 683 | NA | Yes | 549 | 134 | Gender used as a synonym for sex | 27.3 ± 8.7 | NA |
| ***Case-control studies*** | | | | | | | | | | | |
| Abd Elazeem, 2018 | Egypt | RA | HC | 100 | 40 | Yes | 22 | 118 | Sex and gender terms used interchangeably | 48.1 ± 12.4 | 42.4 ± 12.4 |
| Abdelrahman, 2024 | Egypt | RA | HC | 30 | 30 | Yes | 8 | 52 | Gender used as a synonym for sex | 50.0 ± 9.4 | 49.4 ± 9.0 |
| Cakirbay, 2024 | Turkey | RA with Major Depression (MD) | RA without MD | 20 | 20 | No | 9 | 31 | Female/male ratio without specifying whether this refers to sex or gender | 44.1 ± 12.6 | 50.5 ± 10.8 |
| Crosby, 1988 | USA | RA | HC | RA flare: 5 RA non-flare: 10 | 12 | Yes | 7 | 20 | Gender used as a synonym for sex | Mean 44 | |
| Crosby, 1991 | USA | RA | HC | RA flare: 5 RA non-flare: 10 | 12 | Yes | 7 | 20 | Gender used as a synonym for sex | Mean 44 (29-73) | |
| Drewes, Svendsen, 1998 | Denmark | RA | HC | 41 | 19 | Yes | 15 | 45 | NA | 53.2 ± 13.9 | 55.5 ± 17.5 |
| George, 2025 | USA | RA | HC | 913 | 913 | Yes | 640 | 1186 | NA | 65.1 ± 10.1 | 65.2 ± 10.0 |
| Kim, 2018 | South Korea | RA | HC | 123 | 76 | Yes | 0 | 199 | NA | Median 56 (47-61) | 54 (45-61) |
| Manzo, 2020 | Italy | Polymyalgia  Rheumatica | RA | 20 | 33 | Yes | I: 6 C: 12 | I: 13 C: 21 | NA | Age range 61–100 | |
| Rajalingam, 2017 | Malaysia | RA | HC | 46 | 46 | Yes | RA: 3 C: 3 | RA: 43 C: 43 | NA | 48.2 ± 15.0 | 47.1 ± 12.2 |
| Roehrs, 2015 | USA | RA | FM | 16 | 18 | Yes | 0 | RA: 16 FM: 18 | NA | 52 ± 7 | 48 ± 9 |
| Sariyildiz, 2014 | Turkey | RA | HC | 96 | 52 | Yes | RA: 17 HC: 11 | RA: 77 HC: 41 | NA | 46.2 ± 12.3 | 44.1 ± 9.8 |
| Singh, 2024 | India | RA | HC | 112 | 93 | Yes | 38 | 167 | NA | 46.8 ± 11.8 | 45.4 ± 9.8 |
| Xu, 2021 | China | OA RA | HC | OA: 40 RA: 40 | 40 | Yes | 33 | 87 | Gender used as a synonym for sex | OA: 57.9 ± 17.51 RA: 59.4 ± 16.5 | 54.4 ± 15.9 |
| Kwiatkowska, 2018 | Poland | RA OA | HC | RA: 120 OA: 58 | 51 | Yes | 48 | 181 | Gender used as a synonym for sex | RA: 56.8 (range: 19.0-85)  OA: 64.6 (range: 40-93) | 42.8 (range: 22-68) |
| Alburquerque-García, 2015 | Spain | Bilateral KOA | HC | 18 | 18 | Yes | 0 | 36 | NA | 85 ± 4 | 85 ± 6 |
| Campbell, 2015 | USA | KOA with insomnia (KOA-I)  KOA without insomnia | Group 1: primary insomnia (PI) Group 2: HC | KOA-I: 116 KOA: 30 | PI: 27 HC: 28 | Yes | 51 | 150 | NA | KOA-I: 59.2 ± 9.1 KOA: 66.6 ±10.6 | PI: 58.9 ± 8.6 HC: 57.6 ± 7.3 |
| Cho, 2013 | South Korea | Diabetic Peripheral Neuropathy | OA | 199 | 220 | Yes | 128 | 291 | Gender used as a synonym for sex | 62.9 ± 11.0 | 67.7 ± 8.7 |
| Leigh, 1988 | UK | OA | HC | 14 | 16 | Yes | 30 | 0 | NA | Mean 63 (range 52-70) | 62 (52-70) |
| Zhu, 2023 | China | KOA | NA | Group 1:102 Group 2: 116 | NA | Yes | 71 | 147 | NA | 61.4 ± 6.6 | 60.6 ± 7.2 |
| Abdulaziez, 2012 | Egypt | AS | HC | 20 | 10 | Yes | 30 | 0 | NA | 37.8 ± 5.6 | NR |
| Altındağ, 2021 | Turkey | AS | HC | 53 | 47 | Yes | 72 | 28 | Sex and gender terms used interchangeably | 33.4 ± 7.4 | 34.2 ± 6.1 |
| Maatallah, 2022 | Tunisia | axSpA | LBP | 50 | 40 | Yes | I: 39  C: 29 | I: 11  C: 11 | NA | 42.0 ± 12.2 | 46.4 ± 13.8 |
| Yolbaş, 2017 | Turkey | AS | HC | 77 | 49 | Yes | 61 | 65 | NA | 40.6 ± 10.2 | 38.1 ± 10.8 |
| Yüce, 2023 | Turkey | AS | HC | 100 | 100 | Yes | 146 | 54 | Sex and gender terms used interchangeably | 42.0 ± 9.1 | 42.1 ± 9.1 |
| Yüksel, 2019 | Turkey | AS | HC | 20 | 20 | Yes | 30 | 10 | NA | 39.6 ± 11.3 | 39.01 ± 10.6 |
| Jeong, 2014 | South Korea | AS | HC | 40 | 80 | Yes | 67 | 53 | NA | 42.2 ± 12.0 | 43.1 ± 13.0 |
| Durmus, 2015 | Turkey | AS | HC | 80 | 80 | Yes | 108 | 52 | Gender used as a synonym for sex | 39.3 ± 11.0 | 36.4 ± 10.8 |
| Sandikci, 2019 | Turkey | PsA | Psoriasis | 50 | 50 | Yes | PsA: 17 Psoriasis: 22 | PsA: 33 Psoriasis: 28 | NA | 44.7 ± 12.0 | 40.4 ± 13.9 |
| Schneeberger, 2023 | Argentina | PsA | HC | 36 | 37 | Yes | 41 | 32 | Gender used as a synonym for sex | Median 57 (44.2–63.7) | 50 (44.5–61.5) |
| ***Cross-over studies*** | | | | | | | | | | | |
| Fu, 2019 | Australia | Hip OA | NA | 252 | NA | No | 53 | 199 | Only n. of females/males reported, without specifying whether this refers to sex or gender | 62.2 ± 8.3 | NA |
| Lavie, 1991 | Israel | RA | Same population (cross-over study) | 13 | NA | Yes | 0 | 13 | NA | 55.8 ± 10.5 | NA |
| ***Cohort studies*** | | | | | | | | | | | |
| Aydemir, 2025 | Canada | RA | NA | 502 | NA | Yes | 32% | 68% | NA | 56 ± 14 | NA |
| Guaracha-Basáñez, 2023 | Mexico | RA | NA | 187 | NA | Yes | 23 | 164 | NA | Median 41 (32–51.5) | NA |
| Karatas, 2017 | Turkey | RA with high disease activity on anti-TNF | RA on remission, no anti-TNF | 22 | 13 | Yes | 13 | 22 | NA | 55.4 ± 11.4 | 55.0 ± 5.8 |
| Lyne, 2022 | Sweden | RA | NA | 3265 | NA | Yes | 941 | 2324 | NA | Median 61 (Q1–Q3: 50–67) | NA |
| Sarvestan, 2024 | UK | RA | NA | 11 | NA | Yes | 5 | 6 | NA | 75 (70–76.5) | NA |
| Tektonidou, 2020 | Greece | RA PsA AS | NA | RA: 184 PsA: 166 AS: 150 | NA | Yes | RA: 39 PsA: 73 AS: 99 | RA: 145 PsA: 93 AS: 51 | NA | RA: Median 60 (50.8-69) PsA: 51 (41-60.8) AS: 45 (34.3-56) | NA |
| Viitanen, 2020 | Finland | FM | RA | 86 | 74 | Yes | FM: 8  RA: 14 | FM: 78  RA: 60 | NA | 45 ± 8 | 47 ± 8 |
| Bordvik, 2025 | Norway | Hand OA | NA | 299 | NA | Yes | 34 | 265 | NA | 60.7 ± 6.2 | NA |
| Bjurström, 2021 | Sweden | OA | NA | 52 | NA | Yes | 21 | 31 | Gender used as a synonym for sex | 70.4 ± 8.3 | NA |
| Cremeans-Smith, 2006 | USA | OA | NA | 110 | NA | Yes | 35 | 75 | Gender used as a synonym for sex | 69.2 ± 10.2 | NA |
| Chen, 2016 | USA | OA | NA | 34 | NA | Yes | 13 | 21 | NA | 65.4 ± 9.2 | NA |
| Fielden, 2003 | New Zealand | OA | NA | 48 | NA | No | 44% | 56% | Only no. of women/men reported, without specifying whether this refers to sex or gender | 66 ± 12.8 | NA |
| Hawker, 2010 | Canada | OA | NA | 613 | NA | Yes | 137 | 476 | NA | 77.8 ± 7.0 | NA |
| Koken, 2019 | Turkey | OA | NA | 80 | NA | Yes | 23 | 57 | NA | 63.8 ± 6.5 | NA |
| Manning, 2017 | USA | OA | NA | 105 | NA | Yes | 43 | 62 | NA | 62.5 ± 11.7 | NA |
| Mehta, 2025 | India | OA | NA | 104 | NA | Yes | 31 | 73 | NA | 63.8 ± 15 | NA |
| Mont, 2025 | USA | OA | NA | 481 | NA | Yes | NR | NR | NA | NR | NA |
| Mukartihal, 2022 | India | OA | NA | 168 | NA | Yes | 28 | 140 | NA | 64.6 ± 7.5 | NA |
| Myoji, 2015 | Japan | OA | NA | 34 | NA | Yes | 6 | 28 | NA | 61.9 ± 9.5 | NA |
| Owens, 2023 | USA | OA | NA | 109 | NA | Yes | 49 | 57 | NA | 63.9 ± 8.8 | NA |
| Tang, 2017 | USA | OA | NA | 367 | NA | Yes | 79 | 288 | NA | 72.9 ± 8.2 | NA |
| Van Meirhaeghe, 2021 | Australia | OA | NA | 780 | NA | Yes | 364 | 416 | Gender used as a synonym for sex | 69 (range, 42-99) | NA |
| Whibley, 2019 | USA | OA | NA | 160 | NA | Yes | 61 | 99 | NA | Median: 71; IQR: 67−75.5 | NA |
| Er, 2014 | Turkey | KOA | NA | 42 | NA | Yes | 10 | 32 | NA | 65.8 ± 7.8 | NA |
| Fatah, 2020 | Iraq | KOA | NA | 67 | NA | Yes | 16.4% | 83.6% | Gender used as a synonym for sex | 64.2 ± 7.5 | NA |
| Bandinelli, 2025 | Italy | Early-SpA Long-term SpA | NA | Tot: 166 e-SpA: 60 l-SpA: 106 | NA | Yes | 87 | 79 | Gender used as a synonym for sex | e-SpA: 51 (SD: NR) l-SpA: 55.3 ± 1.3 | NA |
| Hu, 2020 | China | AS | NA | 62 | NA | Yes | 56 | 6 | NA | 33.9 ± 6.9 | NA |
| Karatas et al, 2018 | Turkey | AS with high disease activity on anti-TNF | AS on remission, no anti-TNF | 15 | 19 | Yes | 29 | 9 | NA | 37.6 ± 7.6 | 37.2 ± 8.6 |
| Rudwaleit, 2011 | Multinational | AS | NA | 1250 | NA | Yes | 888 | 362 | NA | 43.6 ± 11.4 | NA |
| Tymms, 2022 | Australia | AS | NA | 495 | NA | No | 274 | 220 | Gender used as a synonym for sex | 48.3 ± 13.6 | NA |
| ***Pre-post studies*** | | | | | | | | | | | |
| Farzana, 2020 | India | RA | NA | 30 | NA | Yes | NR | NR | NA | Age range: 20-70 | NA |
| Fragiadaki, 2012 | Greece | RA + sleep disturbances | NA | 15 | NA | No | NA | 15 | Only n. of women reported, without specifying whether this refers to sex or gender | 48 ± 9 | NA |
| Genty, 2017 | France | RA | RA | 99 | NA | Yes | 27.3% | 72.7% | Gender used as a synonym for sex | 58.2 ± 12.1 | NA |
| Zamarrón, 2004 | Spain | RA | NA | 6 | NA | No | NA | 6 | Only n. of female patients reported, without specifying whether this refers to sex or gender | Median: 57 (IR: 17) | NA |
| Kaya, 2016 | Turkey | OA | NA | 199 | NA | Yes | 76 | 123 | NA | 67.8 ± 7.3 | NA |
| Kim, 2015 | South Korea | OA | OA | 23 | 25 | Yes | 0 | 48 | NA | 76.5 ± 6.4 | 73.8 ± 6.0 |
| Zhang, 2021 | China | AS | NA | 35 | NA | Yes | 31 | 4 | Gender used as a synonym for sex | 32.2 ± 5.7 | NA |
| ***Non-RCTs*** | | | | | | | | | | | |
| Detert, 2016 | Germany | RA | RA | 17 | 19 | No | 11 | 25 | Only n. of women without specifying whether this refers to sex or gender | 49.4 ± 13.6 | 48.6 ± 8.8 |
| Taylor-Gjevre,  2011b | Canada | RA | NA | 12 | NA | Yes | 4 | 8 | Gender used as a synonym for sex | 57.6 ± 10.5 | NA |
| Kim, 2014 | South Korea | OA | OA | 22 | 25 | Yes | NA | 47 | NA | 65-70y: 6 71-75y: 1 76-80y: 8 81-85y: 5 ≥86y: 2 | 65-70y: 8 71-75y: 4 76-80y: 7 81-85y: 4 ≥86y: 2 |
| Rosenthal, 2007 | Usa | OA | NA | 34 | NA | Yes | 7 | 27 | NA | 53.7 (range 26–75) | NA |
| Suhas Masilamani, 2025 | India | OA | OA | 200 | NA | Yes | 54 | 146 | Gender used as a synonym for sex | 63.33 ± 7.97 | 62.53 ± 8.56 |
| Taibi, 2011 | USA | OA | NA | 13 | NA | No | NA | 13 | Only n. of women reported, without specifying whether this refers to sex or gender | 65.2 ± 6.9 | NA |
| Coksevim, 2018 | Turkey | AS | AS | Group 1: 20 Group 2: 20 | Group 3: 20 | Yes | 42 | 18 | Gender used as a synonym for sex | Group 1: 33.9 ± 11.0 Group 2: 33.3 ± 10.5 | Group 3: 33.5 ± 11.2 |
| ***RCTs*** | | | | | | | | | | | |
| Aktaş, 2025 | Turkey | RA | RA | Group 1: 18 Group 2: 18 | 18 | Yes | 15 | 39 | Gender used as a synonym for sex | Group 1: 53.9 ± 13.0 Group 2: 51.6 ± 14.8 | 52.9 ± 15.4 |
| Bakir, 2018 | Turkey | RA | RA | 30 | 30 | Yes | 14 | 46 | Gender used as a synonym for sex | 50.4 ± 12.1 | 49.5 ± 16.4 |
| Drewes, 1998 | Denmark | RA | RA | 20 | 20 | No | 11 | 29 | Only n. of female/male patients reported, without specifying whether this refers to sex or gender | 51.2 ± 10.5 | 50.5 ± 8.4 |
| Durcan, 2014 | Ireland | RA | RA | 40 | 38 | No | 28 | 50 | Only n. of female patients reported, without specifying whether this refers to sex or gender | 61 ± 8.0 | 59 ± 12 |
| Kılıç, 2023 | Turkey | RA | RA | 36 | 36 | Yes | 11 | 25 | NA | 44.8 ± 11.2 | 46.2 ± 11.9 |
| Loeppenthin, 2022 | Denmark | RA | RA | 17 | 21 | Yes | I: 13  C: 20 | I: 4  C: 1 | NA | 57.8 ± 9.8 | 54.8 ± 9.6 |
| Lu, 2023 | Taiwan | RA | RA | Group 1: 32 Group 2: 36 | 34 | Yes | 20 | 82 | NA | 55.4 ± 12.2 | 54.9 ± 12.6 |
| McKenna, 2021 | Ireland | RA | RA | 10 | 10 | Yes | 0 | 20 | NA | 58 ± 7.4 | 56 ± 7.9 |
| Palimi, 2022 | Iran | RA | RA | 32 | 32 | Yes | IG: 3  C: 8 | I: 29  C: 24 | NA | 42.15 ± 7.47 | 43.40 ± 6.40 |
| Straub, 2017 | Germany | RA | NA | 31 | NA | Yes | 8 | 23 | NA | 48.6 ± 2.1 | NA |
| Ward, 2018 | New Zealand | RA | RA | 13 | 13 | Yes | I: 0 C: 1 | I: 13 C: 12 | Gender used as a synonym for sex | 50 ± 12 | 59 ± 8 |
| Yousefi, 2023 | Iran | RA | RA | Group 1: 19 Group 2: 19 | 19 | Yes | 9 | 48 | Gender used as a synonym for sex | Group 1: 51.3 ± 5.7 Group 2: 48.7 ± 7.3 | 50.9 ± 6.8 |
| Roth, 2009 | Usa | RA + insomnia | RA + insomnia | 77 | 76 | Yes | 20 | 133 | Gender used as a synonym for sex | 52.3 ± 8.1 | 51.9 ± 9.5 |
| Latocha, 2023 | Denmark | RA with Chronic Insomnia | RA with Chronic Insomnia | 31 | 31 | Yes | 7 | 55 | NA | 60 ± 10 | 57 ± 11 |
| Lu, 2019 | China | RA + sleep disturbances | RA + sleep disturbances | 38 | 38 | Yes | NR | NR | NA | NR | NR |
| Atçi, 2022 | Turkey | OA | OA | Group 1: 26 group 2: 16; group 3: 29; group 4: 31 | 20 | Yes | 25 | 97 | Gender used as a synonym for sex | Group 1: 56.9 ± 5.2 Group 2: 58.5 ± 4.1  Group 3: 57.4 ± 5.4 Group 4: 56.7 ± 5.1 | 57 ± 4.3 |
| Buchanan, 2017 | USA | OA | NA | Group 1: 10 group 2: 7 | NA | Yes | 9 | 8 | Gender used as a synonym for sex | Median: 55 | NA |
| Kivitz, 2006 | USA | OA | OA | 279 | 91 | Yes | 146 | 224 | NA | 62.3 ± 11.2 | 60.0 ± 11.2 |
| Kosinski, 2007 | USA | OA | OA | 815 | 205 | Yes | 322 | 493 | NA | 58.5 ± 10.0 | 56.4 ± 9.8 |
| LeBrun, 2024 | USA | OA | OA | 86 | 86 | Yes | 77 | 95 | NA | 66.8 ± 9.0 | 67.8 ± 8.4 |
| Lee, 2023 | South Korea | OA | OA | 26 | 26 | Yes | 22 | 30 | NA | 78.4 ± 4.6 | 79.3 ± 4.5 |
| Leigh, 1989 | UK | OA | NA (Cross-over design) | 12 | Same patients | Yes | 12 | 0 | NA | 63 (range 48-72) | NA |
| Londhe, 2025 | India | OA | OA | 34 | 34 | Yes | NR | NR | NA | 66.5 ± 12.5 | 63.7 ± 11.7 |
| Lü, 2017 | China | OA | OA | 65 | 65 | Yes | I: 39 C: 41 | I: 26 C: 24 | NA | 60.4 ± 6.9 | 59.8 ± 7.2 |
| McCurry, 2021 | USA | OA | OA | 163 | 164 | Yes | 83 | 244 | NA | 70.1 ± 7.1 | 70.4 ± 6.5 |
| Shakya, 2019 | China | OA | OA | 80 | 80 | Yes | 29 | 131 | NA | 64.5 ± 7.4 | 67.7 ± 9.1 |
| Shourabi, 2024 | Iran | OA | OA | 38 | Sham: 38 Control: 38 | Yes | 16 | 98 | Gender used as a synonym for sex | 48.8 ± 68.87 | Sham: 51.1 ± 13.1 Control: 47.1 ± 61.5 |
| Tang, 2021 | USA | OA | OA | 16 | 15 | Yes | I: 1 C: 1 | I: 15 C: 14 | NA | 66.7 ± 5.2 | 68.9 ± 5.0 |
| Vitiello, 2009 | USA | OA | OA | 23 | 28 | Yes | I: 5 C: 1 | I: 18 C: 27 | Gender used as a synonym for sex | 69.2 ± 8.9 | 66.5 ± 7.7 |
| Von Korff, 2012 | USA | OA | OA | 1) 122 2) 122 | 123 | Yes | NR | NR | NA | ≥ 60 | ≥ 60 |
| Altaş, 2020 | Turkey | KOA | KOA | 20 | 20 | Yes | 9 | 31 | Sex and gender terms used interchangeably | 57.6 ± 8.7 | 55.5 ± 9.6 |
| Babul, 2004 | USA | KOA | KOA | 124 | 122 | Yes | 95 | 151 | NA | 61.2 ± 10.0 | 61.5 ± 10.2 |
| Bertram, 2024 | UK | KOA | KOA | 28 | 29 | Yes | 23 | 34 | Gender used as a synonym for sex | NR | NR |
| Cheung, 2014 | USA | KOA | KOA | 18 | 18 | No | NA | 36 | Only no. of women patients reported, without specifying whether this refers to sex or gender | 71.9 (SD: NR) | 71.9 |
| Döner, 2024 | Turkey | KOA | KOA | Group 1: 20 Group2: 20 | 20 | No | 40 | 20 | Only no. of women reported, without specifying whether this refers to sex or gender | Group 1: 53.1 ± 5.2 Group 2: 53.7 ± 4.8 | 54.9 ± 4.4 |
| Durmuş et al, 2016 | Turkey | KOA | KOA | Group 1: 24 Group 2: 24 | 25 | No | NA | 73 | Only no. of female patients reported, without specifying whether this refers to sex or gender | Group 1: median 56.5 (49-65) Group 2: 57.0 (42-70) | 56.0 (48-70) |
| Lerman, 2017 | USA | OA | OA + insomnia | 50 | 50 | Yes | 21 | 79 | NA | 59.2 ± 9.9 | 59.6 ± 9.1 |
| Heffner, 2018 | USA | KOA + insomnia | KOA + insomnia | 16 | 14 | Yes | 12 | 18 | NA | 60.8 ± 6.6 | |
| Acar, 2023 | Turkey | AS | AS | 28 | 27 | Yes | 31 | 24 | Gender used as a synonym for sex | 44.14 ± 8.0 | 45.33 ± 7.2 |
| Acharya, 2024 | India | AS | NA | Group 1: 15 Group 2: 18 | NA | Yes | 30 | 3 | Gender used as a synonym for sex | Group 1: 32.2 ± 5.7 Group 2: 31.6 ± 6.2 | NA |
| Bao, 2014 | China | AS | AS | 108 | 105 | No | 177 | 36 | Only no. of men patients reported, without specifying whether this refers to sex or gender | 30.5 ± 10.3 | 30.5 ± 8.6 |
| Deodhar, 2020 | Multinational | AS | AS | Tot: 278 Group 1: 138 Group 2: 140 | 78 | No | 255 | 101 | Only no. of men reported, without specifying whether this refers to sex or gender | Median: 38.0 (29.0-46.0) | 41.0  (31.0-50.0) |
| Oksüz , 2021 | Turkey | AS | NA | Group 1: 13 Group 2: 13 | NA | Yes | 18 | 8 | NA | Group 1: 46.2 ± 11.6 Group 2: 41.7 ± 12.5 | NA |
| Sveaas , 2020 | Norway | axSpA | axSpA | 50 | 50 | Yes | 47 | 63 | NA | 45.1 | 47.2 |
| Dubreuil, 2024 | Multinational | BE MOBILE 1: Non-radiographic (nr)-axSpA  BE MOBILE 2: Radiographic (r)-axSpA | BE MOBILE 1: nr-axSpA BE MOBILE 2: r-axSpA | BE MOBILE 1:  128 BE MOBILE 2:  221 | BE MOBILE 1: 126 BE MOBILE 2: 111 | Yes | 378 | 308 | NA | BE MOBILE 1: 39.5 ± 11.1 BE MOBILE 2: 41.0 ± 12.1 | BE MOBILE 1: 39.4 ± 11.8 BE MOBILE 2: 39.2 ± 12.6 |
| Deodhar, 2021 | Multinational | nr-AS | nr-AS | Group 1: 96 Group 2: 102 | 105 | No | 143 | 160 | Only no. of female/male patients reported, without specifying whether this refers to sex or gender | Group 1:50 ± 52.1 Group 2: 49 ± 48.0 | RCT |
| Dougados, 2015 | Multinational | nr-axSpA | nr-axSpA | 106 | 109 | No | 60.5% | 38.5% | Only no. of men patients reported, without specifying whether this refers to sex or gender | 32.0 ± 7.8 | |
| Sieper, 2015 | Multinational | AS nr-axSpA | AS nr-axSpA | Group 1: 111 Group 2: 107 | 107 | Yes | 61.5% | 38.5% | Gender used as a synonym for sex | Group 1: 39.1 ± 11.9 Group 2: 39.8 ± 11.3 | 39.9 ± 12.4 |
| Taibi, 2009 | USA | Inflamamtory arthritis | Inflamamtory arthritis | 7 | 8 | Yes | I: 2 C: 1 | I: 5 C: 7 | NA | 39.3 ± 18.7 | 53.1 ± 10.7 |
| ***Secondary analysis of RCTs*** | | | | | | | | | | | |
| Wells, 2010 | Canada | RA | RA | 1) 258 2) 433 | 1) 133 2) 219 | Yes | 1) I: 59 C: 27 2) I: 95 C: 39 | 1) I: 199 C: 106 2) I: 338 C: 178 | NA | 1) 53.5 ± 12.4 2) 51.5 ± 12.9 | 1) 52.7 ± 11.3 2) 50.4 ± 12.4 |
| Bartlett, 2022 | Multinational | RA | RA | Tot: 2265 Group 1: 826; group 2: 821 | Group 3: 199; group 4: 419 | No | 395 | 1870 | Only no. of female patients reported, without specifying whether this refers to sex or gender | Group 1: 53.2 ± 11.7: Group 2: 52.2 ± 11.6 | Group 3: 52.7 ± 11.6; Group 4: 52.7 ± 11.9 |
| Vitiello, 2014 | USA | OA | NA | 354 | NA | Yes | 76 | 278 | Gender used as a synonym for sex | Improvers: 71.9 ± 7.9 non-improvers: 73.8 ± 8.2 | NA |
| Vitiello, 2022 | USA | OA | NA | 327 | NA | Yes | 85 | 242 | NA | Improvers: 70.1 ± 6.8 non-improvers: 70.3 ± 6.8 | NA |
| Boye Larsen, 2021 | Denmark | Hip or knee OA | NA | 163 | NA | Yes | 99 | 64 | Gender used as a synonym for sex | 66.7 ± 13.6 | NA |
| Florete, 2008 | USA | Hip or knee OA | Hip or knee OA | Group 1: 403 Group 2: 400 Group 3: 400 | 405 | Yes | 593 | 1015 | Gender used as a synonym for sex | Group 1: 59.0 ± 10.5 Group 2: 60.5 ± 10.0 Group 3: 59.07 ± 10.5 | 57.7 ± 10.8 |
| Salwen, 2017 | USA | KOA + insomnia | KOA + insomnia | 36 | 38 | Yes | 17 | 57 | NA | 59.6 ± 9.8 | |
| de Leeuw, 2025 | Netherlands | axSpA | NA | 18 | NA | Yes | 10 | 8 | NA | Median 60.5 (48.0-66.3) | NA |
| ***Protocols*** | | | | | | | | | | | |
| Løppenthin, 2014 | Denmark | RA + sleep disturbances | RA + sleep disturbances | 22 | 22 | Yes | NA | NA | NA | NR at baseline (planned sample: 18–70 yrs) | NA |
| Gohir, 2019 | UK | KOA | KOA | 60 (sample size calculation) | 60 (sample size calculation) | No | NA | NA | NA | NA | NA |

AS, Ankylosing Spondylitis; axSpA, Axial Spondyloarthritis; BME, Bone Marrow Edema; C, Control; CBT, Cognitive Behavioural Therapy; CBT-I, Cognitive Behavioural Therapy for Insomnia; CBT-P, Cognitive Behavioural Therapy for Pain; CBT-PI, Cognitive Behavioural Therapy for Pain and Insomnia; CRP, C-Reactive Protein; DM, Diabetes Mellitus; DMARDs, Disease-Modifying Antirheumatic Drugs; ESS, Epworth Sleepiness Scale; F, Female; FM, Fibromyalgia; GP, General Population; HC, Healthy Controls; I, Intervention; IL-6, Interleukin-6; IBD, Inflammatory Bowel Disease; KOA, Knee Osteoarthritis; LBP, Low Back Pain; M, Male; MD, Major Depression; MOS-SS, Medical Outcomes Study Sleep Scale; MTX, Methotrexate; NA, Not Applicable; NR, Not Reported; OA, Osteoarthritis; pSS, Primary Sjögren’s Syndrome; PsA, Psoriatic Arthritis; QoL, Quality of Life; RA, Rheumatoid Arthritis; RCT, Randomized Controlled Trial; RLS, Restless Legs Syndrome; SSc, Systemic Sclerosis; SpA, Spondyloarthritis; TNF, Tumour Necrosis Factor; TNFi, Tumour Necrosis Factor inhibitor; y, years.
