# Supplementary material for: Sleep measurement and clinical impact in osteoarthritis and inflammatory arthritis: a scoping review
Source: Rheumatology (Oxford). 2026 Aug 11;65(8):keag412. doi: 10.1093/rheumatology/keag412 (PMC13523860; doi:10.1093/rheumatology/keag412)
Supplement: Supplementary file 1 [file keag412_supplementary_data.zip › Supplement_Data.zip!/rhe-25-3089-File010.docx]

**Table S3.** Sleep disorders or impairments prevalence across the included prevalence published studies

| **First author, year** | **Intervention group (IG)** | **Control group (CG)** | **Sleep disorder(s) or impairment** | **Measurement tool** | **Sleep disorder(s) or impairment prevalence (%)** |
| --- | --- | --- | --- | --- | --- |
| Abdelrahman, 2024 | RA | HC | Global Sleep Disturbance | PSQI with cut-off > 5 | 63.3 (RA) vs 13.3 (HC) |
|  |  |  | Insomnia | ISI (cut-off NR) | 26.7 (RA) vs 3.3 (HC) |
| Albayrak Gezer, 2017 | RA | NA | Global Sleep Disturbance | PSQI with cut-off > 5 | 56.4 |
| Anjum, 2022 | RA | NA | Global Sleep Disturbance | PSQI with cut-off > 5 | 77.8 |
| Aydemir, 2025 | RA | NA | Global Sleep Disturbance | PROMIS-SD with cut-off ≥ 55 | 44 |
| Azzam, 2024 | RA | NA | Global Sleep Disturbance | PSQI with cut-off > 5 | 65.3 |
| Bagnato, 2016 | RA SSc | HC | Global Sleep Disturbance | PSQI with cut-off > 5 | 68 (RA) vs 94 (SSc) vs 20 (HC) |
| Baldelli, 2019 | RA  Seronegative SpA | NA | Global Sleep Disturbance | PSQI with cut-off > 5 | 60.9 (RA) vs 66.7 (SpA) |
|  |  |  | RBD and other REM sleep parasomnias | Semi-structured neurological interview following ICSD‐3 criteria | RBD: 3 (RA) vs 0 (SpA)  Nightmare disorder: 34.4 vs 23.1(Tot: 30)  Recurrent  isolated sleep paralysis: 9.4 vs 5.1 (Tot: 8)  REM sleep parasomnias: 35% (Tot) |
| Bilge, 2016 | RA | Type 2 DM | Global Sleep Disturbance | PSQI with cut-off > 5 | 87.2 (RA) vs 79.5 (DM) |
| Bjurström, 2017 | RA | HC | Global Sleep Disturbance | PSQI with cut-off > 5 | 67 (RA) vs 22 (HC) |
|  |  |  | Insomnia | Structured clinical interview (DSM-IV) | 33 (RA) vs 11 (HC) |
| Brahem, 2024 | RA | NA | Global Sleep Disturbance | PSQI with cut-off > 5 | 51 |
| Chau, 2023 | RA | NA | Global Sleep Disturbance | PSQI with cut-off > 5 | 64 |
| Deniz, 2023 | RA | HC | Global Sleep Disturbance | PSQI with cut-off > 5 | 62 (RA) vs 38 (HC) |
| Demir, 2021 | RA | HC | Global Sleep Disturbance | PSQI with cut-off > 5 | 59.7 (RA) vs 31.9 (HC) |
|  |  |  | RLS | IRLSSG criteria | 29.1 (RA) vs 13.8 (HC) |
| Fouda, 2024 | RA | NA | OSA | AHI ≥ 5 (PSG) | 46.7 |
| George, 2025 | RA | HC | Insomnia, hypersomnia, OSA, parasomnia, RLS, PLMD | ICD-9/ICD-10 codes, medical records review | Baseline prevalence (before index date):  any sleep disorders 25 (RA) vs 24 (HC); insomnia 13 vs 11; hypersomnia 1 vs 0; OSA 12 vs 12; parasomnia 0 vs 0; RLS 2 vs 2; PLMD 1 vs 1  Incidence during follow-up:  any sleep disorder 25.6 (RA) vs 22.6 (HC); 5-year cumulative incidence 13 vs 11 |
| Goes, 2017 | RA | NA | Global Sleep Disturbance | PSQI with cut-off > 5 | 81.3 |
| Grabovac, 2018 | RA | NA | Global Sleep Disturbance | MOS-SS (Non-optimal sleep duration= < 7h or > 8h) | 56.8 |
| Guaracha-Basáñez, 2023 | RA | NA | Global Sleep Disturbance | MOS-SS (Non-optimal sleep duration= < 7h or > 8h) | 48.7 |
| Guo, 2016 | RA | HC | Global Sleep Disturbance | PSQI with cut-off > 5 | 78.6 |
| Hammam, 2020 | RA | HC | Global Sleep Disturbance | PSQI with cut-off > 5 | 54.8 (RA) vs 34.8 (HC) |
| Hirsch, 1994 | RA | HC | PLM, RLS, OSA | PSG-based criteria | PLM: 73.7 (RA)  RLS: 68.4 (RA)  Hypopnea: 10.5 (RA) |
| Hughes, 2021 | RA | NA | Global Sleep Disturbance | PSQI with cut-off ≥ 6 | 86.5 |
| Juárez-Rojop, 2023 | RA | NA | Global Sleep Disturbance | PSQI with cut-off ≥ 5 | 49 |
| Karatas, 2017 | RA | NA | Global Sleep Disturbance | PSQI with cut-off ≥ 5 | 60 |
|  |  |  | OSAS, PLM | PSG (AHI with cut-off ≥ 5; PLMI with cut-off ≥ 15) | PLM: 45.7  OSAS: 82.9 |
| Katz, 2023 | RA | NA | Global Sleep Disturbance | MOS-SS (highest quartile; top 25%) | 30 |
|  |  |  | RLS, OSA | Self-reported | RLS: 13  OSA: 18 |
| Kayahan Satış, 2024 | RA | NA | Global Sleep Disturbance | PSQI with cut-off > 5 | 50.7 |
|  |  |  | Insomnia | ISI ≥ 15 | 7.8 |
| Kim, 2018 | RA | HC | Global Sleep Disturbance | PSQI with cut-off > 5 | 61 (RA) vs 39.5 (HC) |
| Kontodimopoulos, 2020 | RA | NA | Global Sleep Disturbance | PSQI with cut-off ≥ 5 | 77.6 |
| Kruthik, 2025 | RA | NA | Global Sleep Disturbance | MOS-SS without cut-off | 28.6 |
| Kumar, 2022 | RA | NA | Global Sleep Disturbance | PSQI with cut-off ≥ 5 | 63.6 |
| Lopes, 2021 | RA | NA | Global Sleep Disturbance | PSQI with cut-off > 5 | 70.9 |
| Løppenthin, Esbensen, Jennum et al., 2015 | RA | NA | Global Sleep Disturbance | PSQI with cut-off > 5 | 61 |
| Luyster, 2011 | RA | NA | Global Sleep Disturbance | PSQI with cut-off > 5 | 61 |
| Lyne, 2022 | RA | NA | Global Sleep Disturbance | Karolinska Sleep Questionnaire with domain-specific cut-offs | ≥1 sleep domain: 38 |
| Miyauchi | RA  pSS | HC | Global Sleep Disturbance | PSQI with cut-off ≥ 6 | 30 (RA) vs 56 (pSS) vs 29.4 (HC) |
| Mustafa, 2019 | RA | NA | Global Sleep Disturbance | PSQI with cut-off > 5 | 20 |
|  |  |  | Insomnia | AIS (cut-off NR) | 63 |
|  |  |  | RLS | IRLSSG criteria | 63 |
| Nicassio, 2012 | RA | NA | Global Sleep Disturbance | PSQI with cut-off ≥ 5 | > 50 |
| Pehlivan, 2016 | RA | RA | Global Sleep Disturbance | PSQI with cut-off > 5 | > 50 |
| Punshi, 2023 | RA | NA | Global Sleep Disturbance | PSQI with cut-off ≥ 5 | 55.8 |
| Purabdollah, 2017 | RA | NA | Global Sleep Disturbance | Sleep Disorders Questionnaire (symptom-based, no cut-off) | 78.6 |
| Radwan, 2021 | RA | HC | Global Sleep Disturbance | PSQI with cut-off > 5 | 54.1(RA) vs 6.6 (HC) |
| Rajalingam, 2017 | RA | HC | Global Sleep Disturbance | PSQI with cut-off > 5 | 47.8 (RA) vs 9.6 (HC) |
| Rezazadeh, 2024 | RA | NA | Global Sleep Disturbance | PSQI with cut-off > 5 | 78.8 |
| Sariyildiz, 2014 | RA | HC | Global Sleep Disturbance | PSQI with cut-off ≥ 6 | 64.1 (RA) |
| Singh, 2024 | RA | HC | Global Sleep Disturbance | PSQI with cut-off ≥ 5 | 92 (RA) vs 28 (HC) |
| Solak, Dündar et al., 2009 | RA | HC | Global Sleep Disturbance | PSQI with cut-off ≥ 5 | 63.6 (RA) vs 25.0 (HC) |
| Son, 2015 | RA | HC | Global Sleep Disturbance | PSQI with cut-off > 5 | 38.5 (RA) vs 13.4 (HC) |
| Szady, 2017 | RA | HC | Global Sleep Disturbance | PSQI with cut-off > 5 | 63.2 |
| Waheed, 2024 | RA | NA | Global Sleep Disturbance | LEVEL  2 – Sleep Disturbance – Adult scale (cut-offs NR) | 41.6 (moderate), 35.6 (severe) |
| Yaseen, 2024 | RA | NA | Global Sleep Disturbance | PSQI with cut-off > 5 | 75.3 |
|  |  |  | Insomnia | Self-reported | 38.7 |
|  |  |  | Sleep apnea |  | 18.2 |
|  |  |  | RLS |  | 15.6 |
|  |  |  | Hypersomnia |  | 10.4 |
|  |  |  | Other sleep disorders |  | 7.8 |
| Akintayo, 2019 | KOA | NA | Global Sleep Disturbance | PSQI with cut-off ≥ 5 | 56.4 |
| Allen, 2008 | Symptomatic hip or knee OA | Adults without symptomatic hip or knee OA | Global Sleep Disturbance | Adapted MOS-SS | Any sleep problems: 76.4% (IG) vs 68.5% (CG) |
|  |  |  | Insomnia |  | 62.9 (IG) vs 53.3 (CG) |
| Taylor, 2018 | OA | NA | Insomnia | ISI with cut-off >10  ICD-9 code, medical records review | 53 (ISI), of whom 22 had an ICD-9 diagnosis |
|  |  |  | OSA | ICD-9 code, medical records review | 33.7 |
| Thorlund, 2025 | Knee or hip OA | NA | Insomnia | ISI-3 with cut-off ≥ 7 | 17 (KOA) vs 20 (HOA) |
| Fertelli, 2019 | KOA | HC | Global Sleep Disturbance | PSQI with cut-off > 5 | 64.9 (KOA) vs 16.3 (HC) |
| Kuralay, 2018 | OA | NA | Global Sleep Disturbance | PSQI with cut-off > 5 | 81.1 |
| Lee, 2019 | OA | NA | Global Sleep Disturbance | PSQI with cut-off ≥ 5 | 41.9 |
| Chen, 2014 | OA | NA | Global Sleep Disturbance | PSQI with cut-off > 5 | 70.3 |
| Cho, 2013 | OA | DPN | RLS | IRLSSG criteria + Hopkins Telephone Diagnostic Interview confirmation | 3.6 (OA) vs 8% (DPN) |
| Hawker, 2020 | OA | NA | Global Sleep Disturbance | PSQI with cut-off > 5 | 70 |
|  |  |  | RLS | IRLSSG criteria | 25.5 |
|  |  |  | Any diagnosed sleep disorder | Self-reported physician-diagnosed | 6.5 |
| Martinez, 2019 | Hip OA | NA | Global Sleep Disturbance | PSQI with cut-off ≥ 5 | 83 |
| Mehta, 2025 | OA | NA | Global Sleep Disturbance | PSQI with cut-off > 5 | 91 |
| Petrov, 2015 | OA | OA | Insomnia | ISI with cut-off ≥ 10 | ≈ 50 |
| Sasaki, 2014 | OA | NA | Global Sleep Disturbance | PSQI with cut-off > 5.5 | 11.5 |
| Yilmaz, 2021 | OA | NA | Global Sleep Disturbance | PSQI with cut-off ≥ 5 | 100 |
| Taylor-Gjevre, 2011a | RA  OA | NA | Global Sleep Disturbance | PSQI with cut-off > 5 | 62 (RA) vs 67 (OA) |
|  |  |  | RLS | IRLSSG criteria | 27 (RA) vs 22 (OA) |
| Fawzy, 2022 | RA KOA | HC | Insomnia | AIS with cut-off > 6 | 75 (RA) vs 25 (KOA) vs 0 (HC) |
| Kwiatkowska, 2018 | RA  OA | HC | Insomnia | AIS with cut-off ≥ 6 | ~71 (RA) vs ~64% OA vs ~33 (HC) |
| Abdulaziez, 2012 | AS | HC | Global Sleep Disturbance | PSQI with cut-off > 5 | 90 (AS) |
| Aydin, 2015 | AS | HC | Global Sleep Disturbance | PSQI with cut-off > 5 | 58.1 (AS) |
| Aykurt Karlıbel, 2023 | axSpA | HC | Global Sleep Disturbance | PSQI with cut-off > 5 | 61 (axSpA) vs 20 (HC) |
| Bandinelli, 2025 | Early-SpA Long-term SpA | NA | Insomnia | DSM-5 criteria | 45 (baseline) |
|  |  |  | Global Sleep Disturbance | PSQI with cut-off > 5 | 51.5 (follow-up; N=17/33) |
| Cai, 2025 | Active and inactive axSpA | HC | Global Sleep Disturbance | PSQI with cut-off ≥ 8 | 78 (active axSpA) vs. 39 (inactive axSpA) vs. 4 (HC) |
| Da Costa, 2009 | SpA | NA | Global Sleep Disturbance | PSQI with cut-off ≥ 5 | 69 |
| Fongen, 2024 | axSpA | NA | Global Sleep Disturbance | PSQI with cut-off > 5 | 85 |
| Frede, 2023 | axSpA PsA | NA | Insomnia | Regensburg Insomnia Scale with cut-offs ≥ 13 (abnormal sleep behavior) and ≥ 25 (severe insomnia) | 46.6 (≥ 13)  4.8 (≥ 25) |
| Gezer, 2017 | PsA | HC | Global Sleep Disturbance | PSQI with cut-off ≥ 6 | 85.4 (PsA) vs 28.9 (HC) |
| Ghani Sayed, 2025 | axSpA | NA | Global Sleep Disturbance | PSQI with cut-off > 5 | 27.5 |
| Günaydin, 2009 | axSpA | NA | Global Sleep Disturbance | PSQI with cut-off ≥ 5 | 55 |
| Hultgren, 2000 | axSpA | HC | Global Sleep Disturbance | Uppsala Sleep Inventory (Yes/no items or dichotomization; scores 4–5 = major problem) | 62 |
| Jiang, 2015 | AS | NA | Global Sleep Disturbance | PSQI with cut-off > 7 | 37.3 |
| Jeong, 2014 | AS | NA | Global Sleep Disturbance | PSQI with cut-off > 5 | 60 |
| Jiang, 2018 | axSpA | HC | Global Sleep Disturbance | PSQI with cut-off > 7 | 37.3 (axSpA) vs 9.5 (HC) |
| Karatas, 2018 | AS with high disease activity on anti-TNF | AS on remission, no anti-TNF | Global Sleep Disturbance | PSQI with cut-off ≥ 5 | 57.9 (tot sample) |
|  |  |  | OSA, PLM | PSG (AHI ≥5; PLMI ≥15) | 73.7; 26.3 (tot sample) |
| Mansoor, 2024 | axSpA | NA | Global Sleep Disturbance | PSQI with cut-off > 5 | 74 |
| Nie, 2018 | axSpA | NA | Global Sleep Disturbance | PSQI with cut-off > 5 | 67.6 |
| Tymms, 2024 | AS | NA | Insomnia | ISA with cut-off > 14 (moderate) and > 21 (severe) | 16 (moderate), 3.2 (severe), 19 (both) |
| Wadeley, 2018 | AS | NA | Global Sleep Disturbance | Jenkins Sleep Evaluation Questionnaire with cut-off ≥ 20 | 19 |
| Ying, 2025 | AS | NA | Global Sleep Disturbance | PSQI (cut-off NR) | 66.8 |
| Yüksel, 2019 | AS | HC | Global Sleep Disturbance | PSQI with cut-off ≥ 5 | 80 (AS) |
|  |  |  | RLS | IRLSSG-2003 | 55 (AS) |
| Yüce, 2023 | AS | HC | Global Sleep Disturbance | PSQI with cut-off > 5 | 57 (AS) |
| Solak, Fidan et al., 2009 | AS | NA | OSAS | PSG (AHI ≥ 5) | 22.6 |
| Song, 2021 | AS | NA | Global Sleep Disturbance | PSQI with cut-off > 5 | 65.4 |
| Song, 2017 | AS | NA | Global Sleep Disturbance | PSQI with cut-off > 7 | ~ 49% |
| Zúñiga-Vera, 2023 | SpA |  | Global Sleep Disturbance | PSQI with cut-off > 5 | 81.9 |
| Krajewska-Włodarczyk, 2018 | PsA  Ps | HC | Global Sleep Disturbance | PSQI with cut-off ≥ 6 | 67.7 (PsA) vs 57.7 (Ps) vs 14.6 |
| Sandikci, 2019 | PsA  Ps | HC | RLS | IRLSSG criteria | 64 (PsA) vs 20 (Ps) vs 14 (HC) |
| Schneeberger, 2023 | PsA | HC | Global Sleep Disturbance | PSQI with cut-off ≥ 5 | 75 (PsA) vs 40.5 (HC) |
| Skougaard, 2023 | PsA | Ps HC | Global Sleep Disturbance | PSQI with cut-off > 5 | 66.1 (PsA) vs 45.0 (Ps) vs 15 (HC) |
| Toledano, 2023 | PsA | NA | Global Sleep Disturbance | PSQI with cut-off > 5 | 63 |
| Wong, 2017 | PsA | Ps HC | Global Sleep Disturbance | PSQI with cut-off > 5 | 84.1 (PsA) vs 69.4 (Ps) vs 50.0 (HC) |

AHI, Apnoea-Hypopnea Index; AIS, Athens Insomnia Scale; AS, Ankylosing Spondylitis; axSpA, Axial Spondyloarthritis; DPN, Diabetic peripheral neuropathy; DM, Diabetes Mellitus; DSM, Diagnostic and Statistical Manual of Mental Disorders; HC, Healthy Controls; ICD, International Classification of Diseases; IRLSSG, International Restless Legs Syndrome Study Group; ICSD, International Classification of Sleep Disorders; ISI, Insomnia Severity Index; KOA, Knee Osteoarthritis; MOS-SS, Medical Outcomes Study Sleep Scale; NA, Not Applicable; NR, Not Reported; OA, Osteoarthritis; OSAS, Obstructive Sleep Apnoea Syndrome; PLM, Periodic Limb Movements; PLMI, Periodic Limb Movements Index; PS, Psoriasis; PsA, Psoriatic Arthritis; PSG, polysomnography; PSQI, Pittsburgh Sleep Quality Index; pSS, Primary Sjögren’s Syndrome; PROMIS-SD, Patient-Reported Outcomes Measurement Information System – Sleep Disturbance; RA, Rheumatoid Arthritis; RDB, REM Sleep Behaviour Disorder; RLS, Restless Legs Syndrome; SpA, Spondyloarthritis; SSc, Systemic Sclerosis; TNF, Tumour Necrosis Factor.
