# Supplementary material for: Sleep measurement and clinical impact in osteoarthritis and inflammatory arthritis: a scoping review
Source: Rheumatology (Oxford). 2026 Aug 11;65(8):keag412. doi: 10.1093/rheumatology/keag412 (PMC13523860; doi:10.1093/rheumatology/keag412)
Supplement: Supplementary file 1 [file keag412_supplementary_data.zip › Supplement_Data.zip!/rhe-25-3089-File011.docx]

**Supplementary Table S4**

Summary of self-reported sleep measures and Ru-SATED dimensions covered across included published studies

| **No.** | **First author, year** | **Self-reported outcome measures (Yes/No)** | **RU-SATED Sleep Health Dimensions** | | | | | | **Other (specify)** | **Tool(s) used (specify)** |
| --- | --- | --- | --- | --- | --- | --- | --- | --- | --- | --- |
|  |  |  | **Regularity** | **Satisfaction** | **Alertness/Sleepiness** | **Timing** | **Efficiency** | **Duration** |  |  |
| *Cross-sectional studies* | | | | | | | | | | |
| 1 | Albayrak Gezer et al., 2017 | Yes | No | Yes | Yes | No | Yes | Yes | Sleeping medicine use, disturbances in sleep | Pittsburgh Sleep Quality Index (PSQI) |
| 2 | Anjum et al., 2022 |  |  |  |  |  |  |  |  |  |
| 3 | Azizli et al., 2023 |  |  |  |  |  |  |  |  |  |
| 4 | Azzam et al., 2024 |  |  |  |  |  |  |  |  |  |
| 5 | Bagnato et al., 2016 |  |  |  |  |  |  |  |  |  |
| 6 | Chau et al., 2023 |  |  |  |  |  |  |  |  |  |
| 7 | Deniz et al., 2023 |  |  |  |  |  |  |  |  |  |
| 8 | Gamal et al., 2020 |  |  |  |  |  |  |  |  |  |
| 9 | Guo et al., 2016 |  |  |  |  |  |  |  |  |  |
| 10 | Hammam et al., 2020 |  |  |  |  |  |  |  |  |  |
| 11 | Juárez-Rojop et al., 2023 |  |  |  |  |  |  |  |  |  |
| 12 | Katz et al., 2016 |  |  |  |  |  |  |  |  |  |
| 13 | Hughes et al., 2021 |  |  |  |  |  |  |  |  |  |
| 14 | Kontodimopoulos et al., 2020 |  |  |  |  |  |  |  |  |  |
| 15 | Kumar et al., 2022 |  |  |  |  |  |  |  |  |  |
| 16 | Lee et al., 2022 |  |  |  |  |  |  |  |  |  |
| 17 | Liu et al., 2022 |  |  |  |  |  |  |  |  |  |
| 18 | Løppenthin, Esbensen, Østergaard et al., 2015 |  |  |  |  |  |  |  |  |  |
| 19 | Luyster et al., 2011 |  |  |  |  |  |  |  |  |  |
| 20 | Mehr et al., 2023 |  |  |  |  |  |  |  |  |  |
| 21 | Minnock et al., 2015 |  |  |  |  |  |  |  |  |  |
| 22 | Miyauchi et al., 2021 |  |  |  |  |  |  |  |  |  |
| 23 | Nicassio et al., 2012 |  |  |  |  |  |  |  |  |  |
| 24 | Pehlivan et al., 2016 |  |  |  |  |  |  |  |  |  |
| 25 | Radwan et al., 2021 |  |  |  |  |  |  |  |  |  |
| 26 | Rezazadeh et al., 2024 |  |  |  |  |  |  |  |  |  |
| 27 | Shen et al., 2021 |  |  |  |  |  |  |  |  |  |
| 28 | Solak, Dündar et al., 2009 |  |  |  |  |  |  |  |  |  |
| 29 | Son et al., 2015 |  |  |  |  |  |  |  |  |  |
| 30 | Punshi et al., 2023 |  |  |  |  |  |  |  |  |  |
| 31 | Szady et al., 2017 |  |  |  |  |  |  |  |  |  |
| 32 | Tuncay et al., 2024 |  |  |  |  |  |  |  |  |  |
| 33 | Ulus et al., 2011 |  |  |  |  |  |  |  |  |  |
| 34 | Allen et al., 2008 |  |  |  |  |  |  |  |  |  |
| 35 | Chen et al., 2014 |  |  |  |  |  |  |  |  |  |
| 36 | Fertelli et al., 2019 |  |  |  |  |  |  |  |  |  |
| 37 | Kuralay et al., 2018 |  |  |  |  |  |  |  |  |  |
| 38 | Lee et al., 2019 |  |  |  |  |  |  |  |  |  |
| 39 | Martinez et al., 2019 |  |  |  |  |  |  |  |  |  |
| 40 | Mesci et al., 2015 |  |  |  |  |  |  |  |  |  |
| 41 | Akintayo et al., 2019 |  |  |  |  |  |  |  |  |  |
| 42 | Aykurt Karlıbel et al., 2023 |  |  |  |  |  |  |  |  |  |
| 43 | Cai et al., 2025 |  |  |  |  |  |  |  |  |  |
| 44 | Sasaki et al., 2014 |  |  |  |  |  |  |  |  |  |
| 45 | Yilmaz et al., 2021 |  |  |  |  |  |  |  |  |  |
| 46 | Aydin et al., 2015 |  |  |  |  |  |  |  |  |  |
| 47 | Mansoor et al., 2024 |  |  |  |  |  |  |  |  |  |
| 48 | Nie et al., 2018 |  |  |  |  |  |  |  |  |  |
| 49 | Ozkan et al., 2016 |  |  |  |  |  |  |  |  |  |
| 50 | Skougaard et al., 2023 |  |  |  |  |  |  |  |  |  |
| 51 | Wong et al., 2017 |  |  |  |  |  |  |  |  |  |
| 52 | Krajewska-Włodarczyk et al., 2018 |  |  |  |  |  |  |  |  |  |
| 53 | Ying et al., 2025 |  |  |  |  |  |  |  |  |  |
| 54 | Zúñiga-Vera et al., 2023 |  |  |  |  |  |  |  |  |  |
| 55 | Jiang et al., 2015 |  |  |  |  |  |  |  |  |  |
| 56 | Ghani Sayed et al., 2025 |  |  |  |  |  |  |  |  |  |
| 57 | Günaydin et al., 2009 |  |  |  |  |  |  |  |  |  |
| 58 | Gezer et al., 2017 |  |  |  |  |  |  |  |  |  |
| 59 | Jiang et al., 2018 |  |  |  |  |  |  |  |  |  |
| 60 | Da Costa et al., 2009 |  |  |  |  |  |  |  |  |  |
| 61 | Fongen et al., 2024 |  |  |  |  |  |  |  |  |  |
| 62 | Urkmez et al., 2020 |  |  |  |  |  |  |  |  |  |
| 63 | İN et al., 2015 |  |  |  |  |  |  |  |  |  |
| 64 | Song et al., 2017 |  |  |  |  |  |  |  |  |  |
| 65 | Toledano et al., 2023 |  |  |  |  |  |  |  |  |  |
| 66 | Grabovac et al., 2018 | Yes | No | Yes | Yes | No | Yes | Yes | Sleep disturbance, snoring, awakening short of breath or with a headache | Medical Outcomes Study Sleep Scale (MOS-SS) |
| 67 | Hamdi et al., 2021 |  |  |  |  |  |  |  |  |  |
| 68 | Kruthik et al., 2025 |  |  |  |  |  |  |  |  |  |
| 69 | Karadağ et al., 2012 |  |  |  |  |  |  |  |  |  |
| 70 | Stocks et al., 2018 | Yes | No | Yes | Yes | No | Yes | Yes | Sleep disturbances categories | MOS Sleep Subscale |
| 71 | Lee et al., 2009 | Yes | No | Yes | No | No | Yes | Yes | NA | MOS Sleep Problems Index II |
| 72 | Lee et al., 2013 |  |  |  |  |  |  |  |  |  |
| 73 | Petzinna et al., 2024 | Yes | No | No | Yes | No | No | No | NA | Epworth Sleepiness  Scale (ESS) |
| 74 | Petrov et al., 2015 | Yes | No | Yes | No | No | No | No | Insomnia severity | Insomnia severity index (ISI) |
| 75 | Thorlund et al., 2025 |  |  |  |  |  |  |  |  |  |
| 76 | Toledano et al., 2024 |  |  |  |  |  |  |  |  |  |
| 77 | Fawzy et al., 2022 | Yes | No | Yes | Yes | No | Yes | Yes | NA | Athens Insomnia Scale (AIS) |
| 78 | Wadeley et al., 2018 | Yes | No | No | Yes | No | Yes | No | NA | Jenkins Sleep Evaluation Questionnaire (JESQ) |
| 79 | Belt et al., 2009 | Yes | No | Yes | Yes | No | Yes | Yes | Sleeping medicine use, nightmares, insomnia | Adapted version of the Sleep Habit Questionnaire of the Social Insurance Institution |
| 80 | Clarke et al., 2013 | Yes | No | Yes | Yes | No | Yes | Yes | NA | St Mary’s Hospital Sleep Questionnaire |
| 81 | Borman et al., 1999 | Yes | No | Yes | No | No | Yes | No | Sleeping medicine use | Sleep component from the Nottingham Health Profile |
| 82 | Cano-García et al., 2021 | Yes | No | Yes | No | No | No | No | Insomnia, hypersomnia | Oviedo Sleep Quality questionnaire |
| 83 | Frede et al., 2023 | Yes | No | Yes | Yes | No | Yes | Yes | Psychophysiological insomnia, sleeping medicine use, focus on sleep | Regensburg Insomnia Scale |
| 84 | Hultgren et al., 2000 | Yes | No | Yes | Yes | No | Yes | Yes | Causes of sleep disturbance: pain, psychosocial stress, others | Uppsala Sleep Inventory |
| 85 | Waheed et al., 2024 | Yes | No | Yes | No | No | Yes | No | NA | PROMIS Sleep Disturbance |
| 86 | Aydemir et al., 2024 | Yes | No | Yes | Yes | No | Yes | Yes | Amount of dreaming | Karolinska Sleep Diary (KSD) |
| 87 | Chalupczak et al., 2025 |  |  |  |  |  |  |  |  |  |
| 88 | Tamm et al., 2021 |  |  |  |  |  |  |  |  | KSD, original questionnaire |
| 89 | Austad et al., 2017 | Yes | No | Yes | No | No | Yes | No | NA | MOS sleep disturbance scale; sleep questions from the Rheumatoid Arthritis Impact of Disease, the Multi-Dimensional Health Assessment Questionnaire and the 15-dimensional (15-D) |
| 90 | Katz et al., 2023 | Yes | No | Yes | Yes | No | Yes | Yes | OSA risk/diagnosis; RLS symptoms/diagnosis, snoring, sleep shortness of breath or headache; self-reported sleep duration | MOS-SS, STOP-Bang, RLS screening Q |
| 91 | Wolfe et al., 2006 | Yes | No | Yes | Yes | No | Yes | Yes | Sleep dsturbance, snoring, awakening short of breath or with a headache | MOS-SS and VAS "How much of a problem has sleep been for you in the past week?" |
| 92 | Baldelli et al., 2019 | Yes | No | Yes | Yes | No | Yes | Yes | Sleeping medicine use, disturbances in sleep, BD and other REM sleep parasomnias | PSQI Semi-structured neurological interview ICSD-3 diagnostic criteria for RBD |
| 93 | Bilge et al., 2017 | Yes | No | No | Yes | No | No | No | Obstructive Sleep Apnea Syndrome (OSAS) risk | ESS, ad hoc questions (OSAS risk) |
| 94 | Purabdollah et al., 2017 | Yes | No | No | Yes | No | No | Yes | NA | Sleep Disorders Questionnaire, ESS |
| 95 | Demir et al., 2021 | Yes | No | Yes | Yes | No | Yes | Yes | Sleeping medicine use, disturbances in sleep, RLS prevalence/severity | PSQI, ESS, IRLSSG-RS scale |
| 96 | Gjevre et al., 2012 | Yes | No | Yes | Yes | No | Yes | Yes | Sleeping medicine use, disturbances in sleep, RLS diagnostic criteria | PSQI, ESS, Berling Questionnaire for Assessmnent of OSA risk |
| 97 | Goes et al., 2017 |  |  |  |  |  |  |  | Sleeping medicine use, disturbances in sleep |  |
| 98 | Gouda et al., 2023 |  |  |  |  |  |  |  |  |  |
| 99 | Goodchild et al., 2010 | Yes | No | Yes | Yes | No | No | Yes | Latency, how they feel refreshed | Ad hoc sleep diary |
| 100 | Hirsch et al., 1994 | Yes | No | Yes | Yes | No | No | Yes | Latency, No. of awakenings | Ad hoc questionnaire |
| 101 | Song et al., 2023 | Yes | No | Yes | Yes | No | No | No | Disturbances in sleep, perceived sleep quality, insomnia severity, emotional stress, comfortable positions | PROMIS sleep disturbance. Computer Adaptive Test |
| 102 | Tabaraii et al., 2021 | Yes | Yes | No | Yes | Yes | Yes | Yes | Sleeping medicine use, snoring, headaches, excessive movement during sleep, | Mini-Sleep Questionnaire and Original questions |
| 103 | Taylor-Gjevre et al., 2011a | Yes | No | Yes | Yes | No | Yes | Yes | RLS diagnostic criteria, sleeping medicine use, disturbances in sleep | PSQI, ESS, Berling Questionnaire for Assessment of OSA risk |
| 104 | İn et al., 2016 | Yes | No | Yes | Yes | No | Yes | Yes | Sleeping medicine use, disturbances in sleep | PSQI, ESS |
| 105 | Bilge et al., 2016 |  |  |  |  |  |  |  |  |  |
| 106 | Yaseen et al., 2024 |  |  |  |  |  |  |  |  |  |
| 107 | Kakazu et al., 2024 |  |  |  |  |  |  |  |  |  |
| 108 | Yeung et al., 2018 |  |  |  |  |  |  |  |  |  |
| 109 | Lopes et al., 2021 |  |  |  |  |  |  |  |  |  |
| 110 | Løppenthin, Esbensen, Jennum et al., 2015 |  |  |  |  |  |  |  |  |  |
| 111 | Brahem et al., 2024 |  |  |  |  |  |  |  |  |  |
| 112 | Bjurström et al., 2017 |  |  |  |  |  |  |  |  |  |
| 113 | Demirci et al., 2016 | Yes | No | Yes | Yes | No | Yes | Yes | Sleeping medicine use, disturbances in sleep, insomnia severity | PSQI, ISI |
| 114 | Bedaiwi et al., 2021 |  |  |  |  |  |  |  |  |  |
| 115 | Kayahan Satış et al., 2025 |  |  |  |  |  |  |  |  |  |
| 116 | Song et al., 2021 | Yes | No | Yes | Yes | No | Yes | Yes | Sleeping medicine use, disturbances in sleep, insomnia severity | PSQI, ESS, ISI |
| 117 | Mustafa et al., 2019 | Yes | No | Yes | Yes | No | Yes | Yes | Sleeping medicine use, disturbances in sleep, functioning during the day | PSQI, ESS, AIS |
| 118 | Westhovens et al., 2014 |  |  |  |  |  |  |  |  |  |
| 119 | Spira et al., 2015 | Yes | No | Yes | Yes | No | Yes | Yes | Latency, perceived sleep quality, insomnia severity | ISI, sleep diary |
| 120 | Taylor et al., 2018 | Yes | No | Yes | Yes | No | Yes | Yes | Latency, perceived sleep quality, insomnia severity | ISI, Berling Questionnaire for Assessment of OSA risk, ICD-9 codes |
| 121 | Adawi et al., 2020 | No |  |  |  |  |  |  |  |  |
| 122 | Hörnberg et al., 2021 |  |  |  |  |  |  |  |  |  |
| 123 | Feehan et al., 2024 |  |  |  |  |  |  |  |  |  |
| 124 | Lošťák et al., 2022 |  |  |  |  |  |  |  |  |  |
| 125 | Murphy et al., 2011 |  |  |  |  |  |  |  |  |  |
| 126 | Parmelee et al., 2017 |  |  |  |  |  |  |  |  |  |
| 127 | Fouda et al., 2014 |  |  |  |  |  |  |  |  |  |
| 128 | Mahowald et al., 1989 |  |  |  |  |  |  |  |  |  |
| 129 | McKenna et al., 2018 |  |  |  |  |  |  |  |  |  |
| 130 | Solak, Fidan et al., 2009 |  |  |  |  |  |  |  |  |  |
| *Case-control studies* | | | | | | | | | | |
| 1 | Sariyildiz et al., 2014 | Yes | No | Yes | Yes | No | Yes | Yes | Sleeping medicine use, disturbances in sleep | PSQI |
| 2 | Singh et al., 2024 |  |  |  |  |  |  |  |  |  |
| 3 | Xu et al., 2021 |  |  |  |  |  |  |  |  |  |
| 4 | Cakirbay et al., 2024 |  |  |  |  |  |  |  |  |  |
| 5 | Kim et al., 2018 |  |  |  |  |  |  |  |  |  |
| 6 | Rajalingam et al., 2017 |  |  |  |  |  |  |  |  |  |
| 7 | Zhu et al., 2023 |  |  |  |  |  |  |  |  |  |
| 8 | Abdulaziez et al., 2012 |  |  |  |  |  |  |  |  |  |
| 9 | Alburquerque-García et al., 2015 |  |  |  |  |  |  |  |  |  |
| 10 | Yüce et al., 2023 |  |  |  |  |  |  |  |  |  |
| 11 | Schneeberger et al., 2023 |  |  |  |  |  |  |  |  |  |
| 12 | Jeong et al., 2014 |  |  |  |  |  |  |  |  |  |
| 13 | Durmus et al., 2015 |  |  |  |  |  |  |  |  |  |
| 14 | Cho et al., 2013 | Yes | No | Yes | Yes | No | Yes | Yes | Sleep disturbance, snoring, awakening short of breath or with a headache, RLS diagnosis | MOS-SS |
| 15 | Manzo et al., 2020 |  |  |  |  |  |  |  | Sleep disturbance, snoring, awakening short of breath or with a headache |  |
| 16 | Maatallah et al., 2022 |  |  |  |  |  |  |  |  |  |
| 17 | Altındağ et al., 2021 | Yes | No | No | Yes | No | No | No | NA | ESS |
| 18 | Drewes, Svendsen et al., 1998 | Yes | No | Yes | Yes | No | Yes | Yes | Frequency of naps | Basic Nordic Sleep Questionnaire |
| 19 | Yolbaş et al., 2017 | Yes | No | Yes | Yes | No | Yes | Yes | Sleeping medicine use, disturbances in sleep, insomnia severity | PSQI, ISI |
| 20 | Yüksel et al., 2019 | Yes | No | Yes | Yes | No | Yes | Yes | Sleeping medicine use, disturbances in sleep; RLS diagnosis | PSQI; International Restless Legs Syndrome Study Group (IRLSSG-2003) |
| 21 | Kwiatkowska et al., 2018 | Yes | No | Yes | Yes | No | Yes | Yes | Predisposition to insomnia | Ford Insomnia Response to Stress Test, AIS |
| 22 | Abd Elazeem et al., 2018 | Yes | No | Yes | Yes | No | Yes | Yes | Sleeping medicine use, disturbances in sleep, insomnia | PSQI, ESS, AIS |
| 23 | Abdelrahman et al., 2024 | Yes | No | Yes | Yes | No | Yes | Yes | Sleeping medicine use, disturbances in sleep, insomnia severity | PSQI, ESS, ISI |
| 24 | Sandikci et al., 2019 |  |  |  |  |  |  |  |  |  |
| 25 | Campbell et al., 2015 | Yes | No | Yes | Yes | No | Yes | Yes | Sleeping medicine use, disturbances in sleep, insomnia severity | PSQI, ESS, ISI, sleep diary |
| 26 | George et al., 2025 | Yes | No | No | Yes | No | No | No | NA | ESS, ICD-9/10 codes, medical records |
| 27 | Roehrs et al., 2015 | No |  |  |  |  |  |  |  |  |
| 28 | Crosby et al., 1988 |  |  |  |  |  |  |  |  |  |
| 29 | Crosby et al., 1991 |  |  |  |  |  |  |  |  |  |
| 30 | Leigh et al., 1988 |  |  |  |  |  |  |  |  |  |
| *Cross-over studies* | |  |  |  |  |  |  |  |  |  |
| 1 | Fu et al., 2019 | Yes | No | Yes | Yes | No | Yes | Yes | Sleeping medicine use, disturbances in sleep | Modified PSQI (over the previous 7 days + specific focus on sleep duration, categorized by standard cut-offs) |
| 2 | Lavie et al., 1991 | No |  |  |  |  |  |  |  |  |
| *Cohort studies* | |  |  |  |  |  |  |  |  |  |
| 1 | Karatas et al., 2017 | Yes | No | Yes | Yes | No | Yes | Yes | Sleeping medicine use, disturbances in sleep | PSQI |
| 2 | Bjurström et al., 2021 |  |  |  |  |  |  |  |  |  |
| 3 | Cremeans-Smith et al., 2006 |  |  |  |  |  |  |  |  |  |
| 4 | Hawker et al., 2010 |  |  |  |  |  |  |  |  |  |
| 5 | Koken et al, 2019 |  |  |  |  |  |  |  |  |  |
| 6 | Mehta et al., 2025 |  |  |  |  |  |  |  |  |  |
| 7 | Mukartihal et al., 2022 |  |  |  |  |  |  |  |  |  |
| 8 | Hu et al., 2020 |  |  |  |  |  |  |  |  |  |
| 9 | Karatas et al, 2018 |  |  |  |  |  |  |  |  |  |
| 10 | Van Meirhaeghe et al., 2021 |  |  |  |  |  |  |  |  |  |
| 11 | Er et al., 2014 |  |  |  |  |  |  |  |  |  |
| 12 | Fatah et al., 2020 |  |  |  |  |  |  |  |  |  |
| 13 | Tektonidou et al., 2020 | Yes | No | Yes | Yes | No | Yes | Yes | Sleep disturbance, snoring, awakening short of breath or with a headache | MOS-SS |
| 14 | Rudwaleit et al., 2011 |  |  |  |  |  |  |  |  |  |
| 15 | Guaracha-Basáñez et al., 2023 |  |  |  |  |  |  |  |  |  |
| 16 | Aydemir et al., 2025 | Yes | No | Yes | No | No | Yes | No | NA | PROMIS Sleep Disturbance |
| 17 | Mont et al., 2025 |  |  |  |  |  |  |  |  |  |
| 18 | Owens et al., 2023 | Yes | No | Yes | No | No | Yes | Yes | Insomnia severity | ISI |
| 19 | Lyne et al., 2022 | Yes | No | Yes | No | No | Yes | Yes | NA | Karolinska Sleep Questionnaire |
| 20 | Myoji et al., 2015 | Yes | No | Yes | No | No | Yes | Yes | Frequent dreaming | Oguri–Shirakawa–Azumi Sleep Inventory, Middle-Aged and Aged Version (OSA-MA) |
| 21 | Bordvik et al., 2025 | Yes | No | Yes | No | No | Yes | No | Sleeping medicine use, sleep disturbances severity | Sleep component from the Norwegian 15D |
| 22 | Viitanen et al., 2020 | Yes | No | No | Yes | No | Yes | No | Day time sleepiness | ESS, Patients' Self-Assessment Multivariant Questionnaire |
| 23 | Chen et al., 2016 | Yes | No | Yes | Yes | No | Yes | Yes | Sleeping medicine use, disturbances in sleep | PSQI, ESS |
| 24 | Fielden et al., 2003 | Yes | No | Yes | Yes | No | Yes | Yes | Daytime napping, Sleeping medicine use, alcohol intake before bedtime, and symptoms of common sleep disorders | Sleep questionnaire adapted from an existing survey tool (including ESS); validated sleep diaries |
| 25 | Manning et al., 2017 | Yes | No | No | Yes | No | No | No | Current sleeping habits, perspectives on the association between sleep quality, joint replacement, and daily activities | ESS, ad-hoc questionnaires |
| 26 | Bandinelli et al., 2025 | Yes | No | Yes | Yes | No | Yes | Yes | Insomnia subtypes (difficulty initiating sleep, difficulty maintaining sleep, early awakening); sleeping medicine use, disturbances in sleep | PSQI, DSM-5 insomnia criteria |
| 27 | Tang et al., 2017 | Yes | No | Yes | Yes | No | Yes | Yes | Sleeping medicine use, disturbances in sleep, insomnia severity | PSQI, ISI |
| 28 | Tymms et al., 2022 | Yes | No | Yes | Yes | No | Yes | Yes | Insomnia severity, API | ISI, Multivariate Apnoea Prediction Index (MAPI) |
| 29 | Whibley et al., 2019 | Yes | No | Yes | No | No | Yes | No | NA | Original questions |
| 30 | Sarvestan et al., 2024 | No |  |  |  |  |  |  |  |  |
| *Pre-post studies* | | | | | | | | | | |
| 1 | Farzana et al., 2020 | Yes | No | Yes | Yes | No | Yes | Yes | Sleeping medicine use, disturbances in sleep | PSQI |
| 2 | Zhang et al., 2021 |  |  |  |  |  |  |  |  |  |
| 3 | Genty et al., 2017 | Yes | No | Yes | Yes | No | Yes | Yes | Frequency of dreams | SSQ |
| 4 | Fragiadaki et al., 2012 | Yes | No | Yes | Yes | No | Yes | Yes | Sleeping medicine use, disturbances in sleep | PSQI, ESS |
| 5 | Kaya et al., 2016 |  |  |  |  |  |  |  |  |  |
| 6 | Zamarrón et al., 2004 | Yes | No | No | Yes | No | No | No | Level of vigilance while driving and ability to perform a task requiring protracted alertness | ESS, steer clear test |
| 7 | Kim et al., 2015 | Yes | No | Yes | No | No | No | Yes | NA | Korean Sleep Scale A, VAS |
| *Non-RCTs* | | | | | | | | | | |
| 1 | Coksevim  et al., 2018 | Yes | No | Yes | Yes | No | Yes | Yes | Sleeping medicine use, disturbances in sleep | PSQI |
| 2 | Suhas Masilamani et al., 2025 |  |  |  |  |  |  |  |  |  |
| 3 | Kim et al., 2014 | Yes | No | Yes | No | No | Yes | No | Sleep pattern, causes of sleep disturbances | Korean Sleep Scale |
| 4 | Taylor-Gjevre et al.,  2011b | Yes | No | Yes | Yes | No | Yes | Yes | RLS diagnostic criteria, sleeping medicine use, disturbances in sleep | PSQI, ESS, Berling Questionnaire for Assessmnent of OSA risk |
| 5 | Taibi et al., 2011 | Yes | No | Yes | Yes | No | Yes | Yes | Sleeping medicine use, disturbances in sleep, insomnia severity | PSQI, ESS, ISI, daily sleep diary |
| 6 | Rosenthal et al., 2007 | No |  |  |  |  |  |  |  |  |
| 7 | Detert et al., 2016 |  |  |  |  |  |  |  |  |  |
| *RCTs* | | | | | | | | | | |
| 1 | Aktaş et al., 2025 | Yes | No | Yes | Yes | No | Yes | Yes | Sleeping medicine use, disturbances in sleep | PSQI |
| 2 | Bakir et al., 2018 |  |  |  |  |  |  |  |  |  |
| 3 | Durcan et al., 2014 |  |  |  |  |  |  |  |  |  |
| 4 | Kılıç et al., 2023 |  |  |  |  |  |  |  |  |  |
| 5 | McKenna et al., 2021 |  |  |  |  |  |  |  |  |  |
| 6 | Palimi et al., 2022 |  |  |  |  |  |  |  |  |  |
| 7 | Lu et al., 2019 |  |  |  |  |  |  |  |  |  |
| 8 | Atçi et al., 2022 |  |  |  |  |  |  |  |  |  |
| 9 | Yousefi et al., 2023 |  |  |  |  |  |  |  |  |  |
| 10 | LeBrun et al., 2024 |  |  |  |  |  |  |  |  |  |
| 11 | Lee et al., 2023 |  |  |  |  |  |  |  |  |  |
| 12 | Londhe et al., 2025 |  |  |  |  |  |  |  |  |  |
| 13 | Lü et al., 2017 |  |  |  |  |  |  |  |  |  |
| 14 | Bertram et al., 2024 |  |  |  |  |  |  |  |  |  |
| 15 | Cheung et al., 2014 |  |  |  |  |  |  |  |  |  |
| 16 | Döner et al., 2024 |  |  |  |  |  |  |  |  |  |
| 17 | Durmuş et al, 2016 |  |  |  |  |  |  |  |  |  |
| 18 | Oksüz et al., 2021 |  |  |  |  |  |  |  |  |  |
| 19 | Sveaas et al., 2020 |  |  |  |  |  |  |  |  |  |
| 20 | Shourabi et al., 2024 |  |  |  |  |  |  |  |  |  |
| 21 | Acar et al., 2023 |  |  |  |  |  |  |  |  |  |
| 22 | Acharya et al., 2024 |  |  |  |  |  |  |  |  |  |
| 23 | Dubreuil et al., 2024 | Yes | No | Yes | Yes | No | Yes | Yes | Frequency of snoring, awakening short of breath or with a headache, and of naps, sleep problems index | MOS- SS-­R Index II |
| 24 | Dougados et al., 2015 | Yes | No | Yes | Yes | No | Yes | Yes | Sleep dsturbance, snoring, awakening short of breath or with a headache | MOS-Sleep Index I and II |
| 25 | Sieper et al., 2015 | Yes | No | Yes | Yes | No | Yes | Yes | Sleep dsturbance, snoring, awakening short of breath or with a headache | MOS-Sleep Problems Index II |
| 26 | Ward et al., 2018 | Yes | No | Yes | Yes | No | Yes | Yes | Insomnia severity | ISI |
| 27 | McCurry et al., 2021 |  |  |  |  |  |  |  |  |  |
| 28 | Heffner et al., 2018 |  |  |  |  |  |  |  |  |  |
| 29 | Bao et al., 2014 | Yes | No | No | Yes | No | Yes | No | Total sleep disturbance score | JSEQ |
| 30 | Deodhar et al., 2020 |  |  |  |  |  |  |  |  |  |
| 31 | Deodhar et al., 2021 |  |  |  |  |  |  |  |  |  |
| 32 | Leigh et al., 1989 | Yes | No | Yes | No | No | No | No | Ease of getting to sleep (GTS), the perceived quality of sleep (QOS), the ease of awakening from sleep (AFS) and the integrity and coordination of behaviour following wakefulness (BFW) | LSEQ |
| 33 | Kivitz et al., 2006 | Yes | No | Yes | No | No | Yes | No | Sleeping medicine use, pain at wake-up | CPSI (Chronic Pain Sleep Inventory) |
| 34 | Babul et al., 2004 |  |  |  |  |  |  |  |  |  |
| 35 | Kosinski et al., 2007 |  |  |  |  |  |  |  |  | CPSI (+ Sleep Problems Index) |
| 36 | Lu et al., 2023 | Yes | No | Yes | Yes | No | Yes | Yes | Sleeping medicine use, disturbances in sleep | PSQI, ESS |
| 37 | Shakya et al., 2019 |  |  |  |  |  |  |  |  |  |
| 38 | Altaş et al., 2020 |  |  |  |  |  |  |  |  |  |
| 39 | Latocha et al., 2023 | Yes | No | Yes | Yes | No | Yes | Yes | Sleeping medicine use, disturbances in sleep, insomnia severity | PSQI, ISI |
| 40 | Loeppenthin et al., 2022 | Yes | Yes | Yes | Yes | No | Yes | Yes | WASO, daytime functioning, sleep during the daytime, hypnotics, time getting up in the morning, time going to sleep, SOL, no. of awakenings | PSQI, ESS, Sleep diary |
| 41 | Tang et al., 2021 | Yes | No | Yes | Yes | No | Yes | Yes | Sleeping medicine use, disturbances in sleep, insomnia severity, TST, SOL, SE, WASO | PSQI, ISI, sleep diary |
| 42 | Drewes et al., 1998 | Yes | No | Yes | Yes | No | Yes | Yes | Frequency of dreams, general evaluation of treatment on sleep, sense of balance and coordination | Spiegel Sleep Questionnaire; Leeds Sleep Evaluation Questionnaire (LSEQ) |
| 43 | Roth et al., 2009 | Yes | No | Yes | Yes | No | Yes | Yes | WASO, SL, TST, depth and quality of sleep, daytime function; insomnia severity | Interactive voice response system, ISI |
| 44 | Von Korff et al., 2012 | Yes | No | Yes | Yes | No | Yes | Yes | SOL, perceived sleep quality, insomnia severity | ISI, sleep diary |
| 45 | Buchanan et al., 2017 | Yes | No | Yes | Yes | No | Yes | Yes | Insomnia severity, sleep disturbance and sleep related-impairment scores | Sleep diary, PROMIS short forms (Sleep Disturbance, Sleep-Related Impairment), ISI |
| 46 | Vitiello et al., 2009 | Yes | No | No | Yes | Yes | Yes | Yes | SLAT, nighttime awakenings, TIB, naps, medication used, TST, WASO, SE | Sleep diary |
| 47 | Lerman et al., 2017 | Yes | No | No | No | No | No | No | WASO | Sleep Diary |
| 48 | Taibi et al., 2009 | Yes | No | Yes | No | No | No | No | No | Original questions |
| 49 | Straub et al., 2017 | No |  |  |  |  |  |  |  |  |
| *Secondary analysis of RCT* | | | | | | | | | | |
| 1 | Wells et al., 2010 | Yes | No | Yes | Yes | No | Yes | Yes | Sleep dsturbance, snoring, awakening short of breath or with a headache | MOS-SS, optimal sleep report |
| 2 | Bartlett et al., 2022 | Yes | No | Yes | Yes | No | Yes | Yes | Sleep dsturbance, snoring, awakening short of breath or with a headache | MOS-SS |
| 3 | Boye Larsen et al., 2021 | Yes | No | Yes | Yes | No | Yes | Yes | Sleeping medicine use, disturbances in sleep | PSQI |
| 4 | Florete et al., 2008 | Yes | No | Yes | No | No | Yes | No | Sleeping medicine use, pain at wake-up | CPSI |
| 5 | Salwen et al., 2017 | Yes | No | No | No | No | Yes | Yes | SE, SOL, TST, and WASO | Sleep diaries (using electronic personal digital assistants) |
| 6 | Vitiello et al., 2014 | Yes | No | Yes | Yes | No | Yes | Yes | Sleeping medicine use, disturbances in sleep; insomnia severity; dysfunctional beliefs about sleep | PSQI, ESS, ISI, Dysfunctional Beliefs and Attitudes about Sleep questionnaire, Functional Outcomes of Sleep Questionnaire |
| 7 | Vitiello et al., 2022 | Yes | No | Yes | Yes | No | Yes | Yes | Sleeping medicine use, disturbances in sleep, insomnia severity | PSQI, ISI |
| 8 | de Leeuw et al., 2025 | No |  |  |  |  |  |  |  |  |
| *Protocols* | | | | | | | | | | |
| 1 | Løppenthin et al., 2014 | Yes | No | Yes | Yes | No | Yes | Yes | Sleeping medicine use, disturbances in sleep | PSQI |
| 2 | Gohir et al., 2019 | Yes | No | Yes | Yes | No | Yes | Yes | Sleeping medicine use, disturbances in sleep | PSQI, ESS |
| *Registered Clinical Trials* | | | | | | | | | | |
| 1 | NCT05861934  NCT04809168  NCT03606707  CTRI/2024/09/073634  ISRCTN61333726 | Yes | No | Yes | Yes | No | Yes | Yes | Sleeping medicine use, disturbances in sleep | PSQI |
| 2 |  |  |  |  |  |  |  |  |  |  |
| 3 |  |  |  |  |  |  |  |  |  |  |
| 4 |  |  |  |  |  |  |  |  |  |  |
| 5 |  |  |  |  |  |  |  |  |  |  |
| 6 | NCT06430918 | Yes | No | No | Yes | No | Yes | No | Total sleep disturbance score | JSEQ |
| 7 | NCT04728581 | Yes | No | Yes | Yes | No | Yes | No | Sense of balance and coordination upon awakening | LSEQ |
| 8 | TCTR20240425005 | Yes | No | Yes | Yes | No | Yes | Yes | // | Modified AIS |
| 9 | NCT04059419 | Yes | No | Yes | Yes | No | Yes | Yes | Insomnia severity | ISI, ESS |
| 10 | RBR-69cyr2  RBR-8pjqh2  CTRI/2021/02/031562 | Yes | No | Yes | Yes | No | Yes | Yes | Sleeping medicine use, disturbances in sleep | PSQI, ESS |
| 11 |  |  |  |  |  |  |  |  |  |  |
| 12 |  |  |  |  |  |  |  |  |  |  |
| 13 | ChiCTR-IOR-16007858 | Yes | No | Yes | Yes | No | Yes | Yes | Sleeping medicine use, disturbances in sleep, insomnia severity | PSQI, ISI |
| 14 | NCT05387473 | Yes | No | Yes | Yes | No | Yes | Yes | Sleeping medicine use, disturbances in sleep, insomnia severity, dysfunctional beliefs about sleep | PSQI, ESS, ISI, DBAS) |
| 15 | NCT00374556 | Yes | No | Yes | No | No | Yes | Yes | TIB (min), WASO, no. of awakenings, insomnia severity | Daily Sleep Diary, VAS, ISI |
| 16 | NCT06527547 | Yes | No | Yes | Yes | No | Yes | Yes | Sleeping medicine use, disturbances in sleep; frequency of snoring, awakening short of breath or with a headache, and of naps, sleep problems index; time into bed, time out of bed | PSQI, MOS-SS, ESS, consenus sleep diary (CSD) |
| 17 | NCT00287794  NCT01919645 | No |  |  |  |  |  |  |  |  |
| 18 |  |  |  |  |  |  |  |  |  |  |

**Supplementary Table S5**

Summary of objective sleep measures and sleep architecture/continuity outcomes covered across the included published studies

| **No.** | **First author, year** | **Sleep Architecture** | | | | | **Sleep Continuity** | | | | | | | **Other** | **Tool(s) used** |
| --- | --- | --- | --- | --- | --- | --- | --- | --- | --- | --- | --- | --- | --- | --- | --- |
|  |  | **NREM (% or min)** | | | **REM (% or min)** | **REM latency (min)** | **Total Sleep Time (TST, min)** | **Timing** | **Sleep latency (SL, min)** | **Sleep efficiency (SE, %)** | **Wake After Sleep Onset (WASO, min)** | **No. of awakenings** | **Sleep Fragmentation Index (SFI)** |  |  |
|  |  | **N1** | **N2** | **N3+N4/Slow-Wave Sleep** |  |  |  |  |  |  |  |  |  |  |  |
| *Cross-sectional* | | | | | | | | | | | | | | | |
| 1 | Adawi et al., 2020 | No | No | No | No | No | Yes | No | No | Yes | Yes | No | No | NA | Mini-Act AMA-32 (Ambulatory Monitoring Inc.) |
| 2 | Tamm et al., 2021 | Yes | Yes | Yes | Yes | No | Yes | No | No | Yes | No | Yes | No | No | Actiwatch Spectrum (Philips Respironics, Bend, OR, USA) |
| 3 | Aydemir et al., 2024 | No | No | No | No | No | Yes | No | Yes | Yes | Yes | Yes | Yes | NA | Actiwatch Spectrum (Philips Respironics, Bend, OR, USA) |
| 4 | Chalupczak et al., 2025 | No | No | No | No | No | Yes | No | Yes | Yes | Yes | Yes | Yes | NA | Actiwatch Spectrum (Philips Respironics, Bend, OR, USA) |
| 5 | Clarke et al., 2013 | No | No | No | No | No | Yes | No | No | Yes | No | No | Yes | NA | Actiwatch (model NS, Cambridge Neurotechnology Ltd.) |
| 6 | Goodchild et al., 2010 | No | No | No | No | No | Yes | No | Yes | Yes | No | No | No | Total awaken time | Actiwatch-Score™ (Cambridge Neurotechnology Ltd., Cambridge, UK) |
| 7 | Spira et al., 2015 | No | No | No | No | No | No | No | No | No | No | No | No | Circadian rhythm parameters (amplitude, MESOR, acrophase, rhythm robustness [F-statistic]) | Actiwatch-2™ (Mini MitterNoPhilips Respironics, Bend, OR, USA) |
| 8 | Chen et al., 2014 | No | No | No | No | No | Yes | No | Yes | Yes | Yes | Yes | No | NA | Wrist-worn actigraph (model NS) |
| 9 | Murphy et al., 2011 | No | No | No | No | No | Yes | No | No | Yes | No | No | No | Time in bed | Actiwatch-Score™ (Cambridge Neurotechnology Ltd., Cambridge, UK) |
| 10 | Parmelee et al., 2017 | No | No | No | No | No | Yes | No | No | Yes | Yes | Yes | Yes | NA | ActiGraph® GT3X-W (ActiGraph LLC, Pensacola, FL, USA) |
| 11 | Lošťák et al., 2022 | No | No | No | No | No | Yes | No | No | No | No | No | No | NA | Axivity AX3 (Axivity Ltd., Newcastle, UK) |
| 12 | McKenna et al., 2018 | No | No | No | No | No | Yes | No | No | No | No | No | No | NA | SenseWear Pro3 Armband™ (BodyMedia Inc., Pittsburgh, PA, USA) |
| 13 | Miyauchi et al., 2021 | No | No | No | No | No | Yes | No | Yes | Yes | No | Yes | No | NA | Motionlogger Actigraph (Ambulatory Monitoring Inc., Ardsley, NY, USA) |
| 14 | Hörnberg et al., 2021 | No | No | No | No | No | Yes | No | No | No | No | No | No | Sleeping heart rate | Actiheart (CamNTech): combined ECG and accelerometer monitor |
| 15 | Feehan et al., 2024 | No | No | No | No | No | Yes | No | No | Yes | No | No | No | Time in bed (laying down sleeping & awake); sleep-awake activities over 24 h | SenseWear Mini™ (BodyMedia Inc., Pittsburgh, PA, USA) |
| 16 | Bjurström et al., 2017 | Yes | Yes | Yes | Yes | Yes | Yes | No | Yes | Yes | Yes | Yes | No | REM duration 1st period (min), REM density | PSG |
| 17 | Fouda et al., 2014 | No | No | No | No | No | No | No | No | No | No | No | No | Apnea index, hypopnea index, apnea-hypopnea index |  |
| 18 | Gjevre et al., 2012 | Yes | Yes | Yes | Yes | No | Yes | No | Yes | Yes | Yes | Yes | No | Number of incidents of apnoeas and hypopnoeas per hour of sleep (AHI), RDI, PLMs, Sleep Stage Transition Index |  |
| 19 | Hirsch et al., 1994 | Yes | Yes | Yes | Yes | Yes | Yes | No | Yes | Yes | Yes | Yes | No | NA |  |
| 20 | Mahowald et al., 1989 | Yes | Yes | Yes | Yes | Yes | Yes | No | Yes | Yes | No | Yes | Yes | Alpha-delta sleep, PLM, apnea-hypopnea index, O2 desaturation) |  |
| 21 | Yeung et al., 2018 | Yes | Yes | Yes | Yes | No | Yes | No | Yes | Yes | Yes | Yes | No | NA |  |
| 22 | İN et al., 2015 | Yes | Yes | Yes | Yes | No | Yes | No | Yes | Yes | No | Yes | No | O₂ saturation |  |
| 23 | İn et al., 2016 | Yes | Yes | Yes | Yes | No | Yes | No | No | Yes | No | Yes | No | AHI; O₂ saturation, desaturation number, arousal index |  |
| 24 | Solak, Fidan et al., 2009 | Yes | Yes | Yes | No | Yes | Yes | No | No | Yes | No | No | No | RDI |  |
| 25 | Petzinna et al., 2024 | No | No | No | No | No | No | No | No | No | No | No | No | AHI, apnea index, hypopnea index, ODI, oxygen saturation | Cardiorespiratory polygraph |
|  | *Case-control studies* |  |  |  |  |  |  |  |  |  |  |  |  |  |  |
| 1 | Crosby et al., 1988 | No | No | No | No | Yes | Yes | No | Yes | Yes | Yes | Yes | No | Number of sleep stage changes | PSG |
| 2 | Crosby et al., 1991 | No | No | No | No | No | Yes | No | No | Yes | No | Yes | No | NA |  |
| 3 | Drewes, Svendsen et al., 1998 | Yes | Yes | Yes | Yes | Yes | Yes | No | Yes | Yes | Yes | Yes | No | No. of stage shifts, arousal index, apnoea index, PMLs, total movements per hour, NREM (N1, N2, N3, N4) latency |  |
| 4 | George et al., 2025 | Yes | Yes | Yes | Yes | Yes | Yes | No | Yes | Yes | No | No | No | CPAP data, mean O₂ saturation |  |
| 5 | Roehrs et al., 2015 | Yes | Yes | Yes | Yes | Yes | Yes | No | Yes | Yes | Yes | Yes | No | NA |  |
| 6 | Leigh et al., 1988 | Yes | Yes | Yes | Yes | Yes | Yes | No | Yes | Yes | Yes | Yes | No | NA |  |
| 7 | Abdulaziez et al., 2012 | Yes | Yes | Yes | Yes | Yes | No | No | Yes | Yes | No | No | No | Respiratory disturbance index (RDI), periodic leg movement index (PLMI), arousal index |  |
| 8 | Campbell et al., 2015 | Yes | Yes | Yes | Yes | Yes | Yes | No | Yes | Yes | Yes | No | No | Apnea Hypopnea Index, Respiratory Disturbance Index, Average SPO2 Low during sleep (min), Periodic Limb Movement Index (PLMI), PLMI with Arousal Index, Spontaneous Arousal Index, Overall Arousal Index | Actigraph 2 (Philips Healthcare/Respironics, Bend, OR) |
| 9 | Xu et al., 2021 | / | No | No | No | No | Yes | No | Yes | Yes | Yes | No | No | NA | SenseWear Armband™ (SWA; Model MF-SW; BodyMedia Inc., Pittsburgh, PA, USA) |
| *Cross-over studies* | | | | | | | | | | | | | | | |
| 1 | Lavie et al., 1991 | Yes | Yes | Yes | Yes | No | Yes | No | Yes | Yes | Yes | Yes | No | PLMs, sleep apnea | PSG (EEG, EMG, EOG, ECG, actigraphy, respiratory monitoring) |
| *Cohort studies* | | | | | | | | | | | | | | | |
| 1 | Karatas et al., 2017 | Yes | Yes | Yes | Yes | No | Yes | No | No | Yes | No | Yes | Yes | AHI, Apnea–Hypoapnea Index, PLMI, snoring score | PSG |
| 2 | Karatas et al, 2018 | Yes | Yes | Yes | Yes | No | No | No | No | No | No | No | Yes | AHI, Apnea–Hypoapnea Index, PLMI |  |
| 4 | Sarvestan et al., 2024 | No | No | No | No | No | Yes | No | No | No | No | Yes | No |  | Axivity AX6 (Axivity Ltd, York, UK) |
| 5 | Fielden et al., 2003 | No | No | No | No | No | Yes | No | Yes | Yes | No | No | Yes | Assumed sleep, actual sleep, sleep activity (average amount of activity per minute during the assumed sleep time), time in bed | Actiwatch-16™ (Mini Mitter, Bend, OR, USA) |
| 6 | Myoji et al., 2015 | No | No | No | No | No | Yes | No | No | Yes | No | Yes | No | Yes (longest continuous sleep time) | Motionlogger Actigraph (Ambulatory Monitoring Inc., Ardsley, NY, USA) |
| 7 | Whibley et al., 2019 | No | No | No | No | No | Yes | No | Yes | Yes | Yes | No | No | NA | Actiwatch-Score™ (Philips Respironics/Mini Mitter, Bend, OR, USA) |
| *Pre-post studies* | | | | | | | | | | | | | | | |
| 1 | Zamarrón et al., 2004 | Yes | Yes | Yes | Yes | Yes | Yes | No | Yes | Yes | No | No | No | Sleep stage transitions/hour, no. of arousals/hour | PSG |
| *Non-RCTs* | | | | | | | | | | | | | | | |
| 1 | Detert et al., et al., 2016 | Yes | Yes | Yes | Yes | No | Yes | No | No | Yes | Yes | No | No | NA | PSG |
| 2 | Taylor-Gjevre et al., 2011b | Yes | Yes | Yes | Yes | No | No | No | Yes | Yes | Yes | Yes | No | NA |  |
| 3 | Rosenthal et al., 2007 | Yes | Yes | Yes | Yes | Yes | Yes | No | Yes | Yes | Yes | Yes | No | NA |  |
| 4 | Taibi et al., 2011 | No | No | No | No | No | Yes | No | Yes | Yes | Yes | No | No | NA | Actiwatch (Mini Mitter Company, Inc., Bend, OR, USA) |
| *RCTs* | | | | | | | | | | | | | | | |
| 1 | Drewes et al., 1998 | Yes | Yes | Yes | Yes | No | Yes | No | Yes | Yes | Yes | Yes | No | Number of stage shifts, arousal index, apnoea index, periodic movements of the legs (PMLs), total movements per hour | PSG |
| 2 | Latocha et al., 2023 | No | No | No | No | No | Yes | No | No | Yes | No | No | No | NA |  |
| 3 | Leigh et al., 1989 | Yes | Yes | Yes | Yes | Yes | Yes | Yes | Yes | Yes | Yes | Yes | No | NA |  |
| 4 | Loeppenthin et al., 2022 | Yes | Yes | Yes | Yes | No | Yes | No | Yes | Yes | Yes | No | No | Apnea-Hypopnea Index (AHI), leg movements |  |
| 5 | Straub et al., 2017 | Yes | Yes | Yes | Yes | No | Yes | No | No | Yes | Yes | No | No | NA |  |
| 6 | Taibi et al., 2009 | No | No | No | No | No | Yes | Yes | Yes | Yes | Yes | No | Yes | NA | Mini-Motionlogger Actigraph (Ambulatory Monitoring Inc., Ardsley, NY, USA) |
| 7 | Von Korff et al., 2012 | No | No | No | No | No | No | No | No | Yes | No | No | No | NA | Actiwatch-2™ (Mini Mitter/Philips Respironics, Bend, OR, USA) |
| *Secondary analysis of RCTs* | | | | | | | | | | | | | | | |
| 1 | de Leeuw et al., 2025 | No | No | No | No | No | Yes | No | No | No | No | No | No | Light and deep sleep duration (hours/min, WAT-estimated) | Withings PULSE HR® (Withings, Issy-les-Moulineaux, France) |
| 2 | Salwen et al., 2017 | No | No | No | No | No | Yes | No | Yes | Yes | Yes | No | No | NA | Actiwatch-2™ (Mini Mitter/Philips Respironics, Bend, OR, USA) |
| 3 | Vitiello et al., 2014 | No | No | No | No | No | No | No | No | Yes | No | No | No | NA | Actiwatch-2™ (Mini Mitter/Philips Respironics, Bend, OR, USA) |
| *Protocols* | | | | | | | | | | | | | | | |
| 1 | Løppenthin et al., 2014 | Yes | Yes | Yes | Yes | Yes | Yes | No | Yes | Yes | Yes | Yes | Yes | NA | PSG |
