# Supplementary material for: Sleep measurement and clinical impact in osteoarthritis and inflammatory arthritis: a scoping review
Source: Rheumatology (Oxford). 2026 Aug 11;65(8):keag412. doi: 10.1093/rheumatology/keag412 (PMC13523860; doi:10.1093/rheumatology/keag412)
Supplement: Supplementary file 1 [file keag412_supplementary_data.zip › Supplement_Data.zip!/rhe-25-3089-File012.docx]

**Supplementary Table S6**

**Intervention characteristics and key findings of the included published studies**

| **First author, year** | **Intervention** | | | **Comparator** | | | **Key findings** |
| --- | --- | --- | --- | --- | --- | --- | --- |
|  | **Type** | **Duration** | **Other characteristics** | **Type** | **Duration** | **Other characteristics** |  |
| Acar, 2023 | Tele-yoga classes including warm-up, vinyasa flow, pranayama, deep relaxation, and meditation | 8 weeks, 3 sessions/week, 60 min/session | Conducted via Zoom by a Yoga Alliance–registered instructor and physiotherapist; max 5 participants per group | Wait-list (usual care/routine activities) | 8 weeks | Instructed not to start any new exercise program; offered tele-yoga after study completion | Tele-yoga improved function, disease activity, spinal mobility, flexibility, muscular endurance, balance, sleep quality, stress, depression, and QoL vs. control. Intervention was safe and well-tolerated in AS patients |
| Acharya, 2024 | Group 1: supervised yoga sessions  Group 2: conventional exercise program | 8 weeks, 3x/week | // | NA | NA | NA | Both yoga and traditional exercise groups showed significant within-group improvements in mobility function, sleep quality, chest expansion, and pain post-treatment and at FU. No statistically significant between-group differences were observed in any of the outcomes |
| Aktaş, 2025 | Group 1: warm saltwater hand-foot bath  Group 2: warm water hand-foot bath | 6 weeks, 20 min/day (between 21.00-22.00) | Instructions for the hand-footh bath were given through a video-based demonstration training | Usual care | // | // | Both types of baths improved pain, fatigue, and sleep quality in RA. Saltwater baths gave the greatest pain relief and improved function, while warm water baths most reduced fatigue and fatigue-related QoL |
| Altaş, 2020 | 15 sessions of exercise therapy + 10 consecutive physical therapy sessions + home‑based programme of isometric and isotonic exercises | Exercise therapy sessions: NR  Physical therapy sessions: 1/day, 5 days/week; each included 20 min of hot‑pack, 20 min of TENS and 5 min of therapeutic ultrasound (US).  Home-based exercises: 10 repetitions, 3 times/day | // | Same as the IG except for the physical therapy sessions | Same as the IG | Same as the IG | After 10 sessions of physical therapy, the IG showed greater improvement in sleep quality and daytime sleepiness vs. exercise-only group; pain, depression and QoL also improved more in the IG |
| Atçi, 2022 | Group 1: 10 consecutive outpatient physical therapy sessions  Group 2: 10 outpatient spa treatment sessions  Group 3: inpatient physical therapy  Group 4: inpatient spa treatment | Group 1: 1 hour/day, 5 days/week; Group 2: 5 weeks; 20 min/day; 2 times/week; Group 3: 2 weeks; 1 hour/day, 5 days/week; Group 4: NR | // | Only paracetamol in case of pain | Followed up for 15 days | // | OA impairs sleep; all interventions improved sleep quality in patients with OA, but only the inpatient spa treatment group showed a statistically significant improvement in PSQI scores |
| Azizli, 2023 | Use of DMARDs | 6 months | // | NA | NA | NA | DMARDs improved sleep quality and QoL after 6 months in older RA patients; poor sleepers had higher disease activity and lower QoL |
| Babul, 2004 | Tramadol ER (starting 100 mg once daily, titrated up to 200–400 mg once daily as tolerated) | 12 weeks | // | Placebo | 12 weeks | // | Tramadol ER once daily was effective in reducing OA pain and improving physical function and sleep quality vs placebo, though associated with more adverse events (79% vs 64%; mainly nausea, constipation, dizziness, headache, somnolence; mostly mild/moderate) |
| Bagnato, 2016 | Corticosteroid therapy: 12 RA, 4 SSc  DMARDs therapy: 25 RA, 13 SSc  Biologics: 22 RA  DMARDs + Biologic therapy: 13 RA | NR | // | NA | NA | NA | PSQI scores higher in SSc vs RA and HC; in RA, higher disease activity and depression associated with poor sleep; corticosteroid treatment associated with worse sleep |
| Bao, 2014 | Golimumab 50 mg SC q4w from baseline (week 0) to week 48 | 48 weeks | // | Placebo subcutaneous injections every 4 weeks (week 0–20), then switched to golimumab 50 mg SC q4w (week 24-48) | 48 weeks | At week 16, “early escape” allowed if <20% improvement in pain + morning stiffness → switched to golimumab 50 mg | Golimumab significantly improved ASAS20 at week 14 (49.1% vs 24.8%, p<0.001) and week 24 (50.0% vs 22.9%, p<0.001). Greater improvements in BASFI and BASMI vs placebo. Significant improvements in SF-36 (physical + mental scores) and JSEQ sleep scores at week 24, with further gains through week 52. Adverse event rates were comparable between golimumab and placebo groups, no unexpected safety concerns |
| Bakir, 2018 | Foot reflexology + routine medical treatment | 6 weeks, weekly 30-minute session | // | Routine medical treatment | NR | // | Foot reflexology significantly improved pain and sleep quality in RA patients compared to CG after 6 weeks of treatment |
| Bartlett, 2022 | Group 1: Tofacitinib 5 mg BID + csDMARDs Group 2: Tofacitinib 10 mg BID + csDMARDs | ORAL Standard/Sync = 12 months; ORAL Scan = 24 months | // | Group 3: adalimumab 40 mg Q2W;  group 4: placebo | Group 3: 12 months (ORAL Standard trial) Group 4: 3–6 months, then switch to tofacitinib | Same background csDMARDs therapy | Tofacitinib significantly improved sleep, fatigue, and HRQoL compared to placebo; improvements observed by month 1 and sustained through 6–12 months |
| Bertram, 2024 | 1 pre-op clinic session (3 months before surgery) with assessment, sleep education/hygiene, SMART goals, and shared decision on an evidence-based sleep intervention (e.g., digital CBT-I “Sleepstation”, Calm/Headspace audio) + usual care | 4-week FU phone call to review/adjust plan | // | Usual care | Assessment 3 months pre-op, 1 week pre-op and 3 months post-op (as the intervention group) | // | With some amendments to processes and design, an RCT to evaluate the clinical and cost-­effectiveness of the REST intervention is feasible |
| Bjurström, 2021 | THA | 1 day | // | NA | NA | NA | Poor pre-op sleep predicted worse 6-mo pain outcomes, higher opioid use, more pain sensitivity |
| Boye Larsen, 2021 | TKA or THA | // | // | NA | NA | NA | Poor pre-op sleepers had higher pre-op pain, catastrophizing, anxiety. High pre-op pain intensity, but not poor sleep quality, was associated with higher chronic post-op pain intensity |
| Buchanan, 2017 | Group 1: shared yoga (practiced with a partner) Group 2: individual yoga (without a partner) | 12 weeks, weekly 75-min Viniyoga classes, 5-min morning “warm up” routine, daily 30-min home practice | The program included relaxation and partner support in group 1 | NA | NA | NA | Both yoga formats were feasible and led to improvements in ISI and PROMIS sleep disturbance scores, with no significant differences between the SY and IY groups |
| Cremeans-Smith, 2006 | TKR | Assessment at 2–3 weeks pre-op, 1-month post-op and 3 months post-op | // | NA | NA | NA | Sleep disruptions at 1 month (PSQI) mediated the relationship between post-op pain and functional limitations at 3 months. Highlights the importance of adequate sleep for faster and better recovery after TKR |
| Chen, 2016 | TKA | // | // | NA | NA | NA | Sleep worsened in the first weeks after surgery but improved by three months. Pain decreased and sleep changes were not directly related to pain |
| Cheung, 2014 | Hatha yoga | 8 weeks, weekly 60-minute group sessions + 30-min home-based session, 4x/week | // | Wait-list | Second baseline assessment at week 8, then received same 8-week yoga program, with FU to week 20 | // | An 8-week yoga program improved pain, stiffness, function, and chair stand performance in older women with KOA. Sleep disturbance improved, but overall sleep quality declined at 20 weeks; the program was safe and well accepted |
| Kim, 2014 | Foot reflexology | 4 weeks, 2x/week | // | No treatment | // | // | Foot reflexology improved joint pain, stiffness, depression, and sleep quality in older women with OA |
| Coksevim, 2018 | Group 1: Anti-TNF + GPR Group 2: Anti-TNF + conventional exercise program | 3 months, daily session of at least 30-min, 5 days/week | Step-by-step written instructions with illustrations + weekly telephone monitoring by the researchers | Group 3: conventional exercises program + usual therapy (NSAIDs and/or SLZ) | 3 months, daily session of at least 30-min, 5 days/week | // | Both intervention groups improved in all evaluated clinical parameters, including sleep quality; Group 1 (anti-TNF + GPR) > Group 2 (anti-TNF + conventional exercise) in pain, walking performance, and mobility |
| Deodhar, 2010 | Subcutaneous golimumab 50 mg (group 1) or 100 mg (group 2) | Switched to golimumab 50 mg at week 16 for non-responders (early escape) | Non-responders in group 1 switched to golimumab 100 mg at week 16 (early escape) | Subcutaneous placebo | Every 4 weeks up to 24 weeks | Switched to golimumab 50 mg at week 16 for non-responders (early escape) | Patients with AS had moderate-to-severe sleep disturbance at baseline. Golimumab significantly improved sleep quality at weeks 14 and 24 vs. placebo. Improvements correlated with better QoL, function, pain, and disease activity; reduction in night back pain was the strongest predictor of improved sleep |
| Deodhar, 2021 | Subcutaneous 80-mg ixekizumab | Group 1: every 4 weeks (QW4) Group 2: every 2 weeks (QW2) Both up to 52 weeks | Switch to open-label ixekizumab Q2W or TNFi allowed between weeks 16–44 | Subcutaneous placebo | Every 2 weeks up to 52 weeks | Same switch option | Ixekizumab (Q2W and Q4W) improved sleep, work productivity, and activity impairment vs placebo through Week 52. In the Q4W group, improvements in presenteeism and overall work impairment were sustained from Week 16 to 52; in the Q2W group, improvements in activity impairment were sustained over the same period |
| Detert, 2016 | ETA + MTX (one patient received only ETA) | 16 weeks, ETA 25 mg x 2 times/weeks, MTX 12.5–17 mg/week | // | MTX | 16 weeks, MTX 12.5–17 mg/week | NA | Both ETA and MTX reduced disease activity, CRP, and improved function and QoL at 16 weeks. Only ETA increased SE, TST, and N2 sleep, while MTX showed no significant sleep changes |
| Dougados, 2015 | ETN 50 mg subcutaneous | 12 weeks, once/week | Stable NSAID allowed | Placebo | 12 weeks | Stable NSAID allowed | At baseline, patients had impaired sleep (MOS Sleep Index), fatigue, and poor HRQoL compared to norms. At week 12, ETN significantly improved global disease activity, pain, and physical function. Sleep (MOS Sleep Index I & II) showed a trend toward improvement with ETN, but not statistically significant. By week 24, patients switching from placebo to ETN also improved across QoL domains. |
| Döner, 2024 | Aromatherapy message (with Effleurage and Petrisage techniques) to the affected knee(s) using BEO (group 1) or pure sweet almond (placebo, group 2) + conventional physiotherapy (TENS, hot pack, US) | 4 weeks, biweekly 15-min aromatherapy message 4 weeks, 45-min sessions, 5 days/week | // | Only conventional physiotherapy | 4 weeks, 45-min sessions, 5 days/week | // | All groups improved in pain, function, sleep quality, and menopausal symptoms. Aromatherapy massage with BEO was superior to control and placebo for pain, function, and menopausal symptoms, but showed no additional benefit for sleep quality |
| Drewes, 1998 | 7.5 mg zopiclone | 2 weeks, 1 tablet/day | Any other psychotropic drugs discontinued 14 days before the study | Similar placebo tablet | 2 weeks, 1 tablet/day | Any other psychotropic drugs discontinued 14 days before the study | Zoplicone improved subjective sleep but not pain or other clinical outcomes. PSG showed only minor changes, while EEG suggested altered sleep microstructure. Zoplicone may be of value for subjective sleep complaints, but it is doubtful whether hypnotics improve daytime symptoms in RA patients |
| Dubreuil, 2024 | Subcutaneous BKZ 160 mg | Up to 52 weeks, every 4 weeks | // | Subcutaneous placebo | Up to 52 weeks, every 4 weeks | Placebo until week 16, then all switched to BKZ | BKZ significantly improved physical function, QoL, and PROs vs. placebo at Week 16, with sustained benefits to Week 52. Sleep and work productivity also improved, particularly in patients with impaired baseline sleep |
| Durcan, 2014 | Home-based exercise intervention | 12 weeks, resistance 3×/week, ROM daily, walking daily, balance 2–3 x/week | // | Advice only on the benefits of exercise | 12-week FU | // | The exercise program resulted in significant improvement in functioning, pain, stiffness, sleep quality and fatigue vs usual care |
| Durmuş, 2016 | Group 1: PP with capsaicin + exercise therapy Group 2: topical agents as capsaicin gel + exercise therapy | 6 weeks, 3x/week | // | Exercise therapy only | 6 weeks, 3x/week | // | All groups improved significantly in pain, disability, functionality, sleep quality, and depression. Pain reduction was greatest in the PP group (maintained at FU). Capsaicin gel was as effective as PP for functionality, disability, and sleep quality, and superior to exercise alone for functionality and sleep |
| Er et al., 2014 | TKA; standard surgical protocol | Evaluation baseline and 3 months post-op | // | NA | NA | NA | Sleep quality and pain improved significantly after TKA; no correlation was found between these two parameters |
| Farzana, 2020 | JPRT | 10 days, 1 session/day | // | NA | NA | NA | Sleep disturbance and QoL significantly improved after JPRT in chronic RA |
| Fatah, 2020 | TKA (by different surgeons from different hospitals) | Evaluation baseline, immediately, 1 month and 3 months post-op | // | NA | NA | NA | Pain decreased steadily during FU, while sleep quality improved immediately post-op, worsened at 1 month, and then gradually improved by the end of FU |
| Fielden, 2003 | THA | Assessment at least 4 days pre-op and 3 months post-op | NA | NA | NA | NA | After THA, 75% of participants reported no or rare sleep disturbance from hip pain. Actigraphy showed reduced time in bed, lower activity during sleep, greater sleep efficiency, and less fragmentation. Improvements were more evident in patients <65 year |
| Florete, 2008 | Tramadol ER 100 mg (group 1), 200 mg (group 2), 300 mg (group 3) | 12 weeks, once/daily | NA | Placebo | 12 weeks, once/daily | // | Tramadol ER significantly improved sleep quality vs. placebo from week 1 onward, with sustained benefits through 12 weeks. Improvements included reduced nocturnal and morning awakenings due to pain and less difficulty falling asleep. Pain reduction was associated with fewer pain-related sleep disturbances |
| Fragiadaki, 2012 | Tocilizumab 8 mg/kg | 6 months, once/monthly | // | NA | NA | NA | Tocilizumab improved sleep quality and reduced daytime sleepiness in RA, independent of disease activity, suggesting IL-6 involvement in sleep disturbances |
| Genty, 2017 | Biologic therapy (anti-TNF n=50, tocilizumab n=19, abatacept n=16, rituximab n=14) | 3 months | // | NA | NA | NA | Fatigue improved in 59% of patients after 3 months, sleep quality in 26%, and depression in 34%; fatigue improvement was mainly linked to reduced disease activity, not sleep changes |
| Gohir, 2019 | Web-based exercise programme |  |  | Standard community care provided by GPs in the UK |  |  | This study is a protocol. They have published the main results on the primary outcomes (e.g., pain) but the results on sleep will be published on another paper |
| Hu, 2020 | Corrective spinal surgery | Assessment at pre-op and 24 months post-op | // | NA | NA | NA | Pre-op: 82% “poor sleepers”. Post-op: significant improvements in total PSQI and subscales (except sleep medication use). Improvements correlated with sagittal realignment parameters |
| Heffner, 2018 | CBT-I | 6 weeks | // | No intervention, 6 "check-in” calls | 6 weeks, 1x/week | // | Improvement of insomnia was linked to better physical function, reduced knee pain during transfers, and favorable inflammatory responses (lower IL-6 rise, less TNF-α decrease) |
| Kivitz, 2006 | Oxymorphone ER tablets, 10 mg, 40 mg, 50 mg, q12h (BID) | 12 weeks (double-blind treatment) | Multicentre, randomized, dose-ranging Phase III trial; patients had moderate severe OA pain (hip/knee); both opioid-naïve and experienced included | Placebo tablets, q12h (BID) | 12 weeks | Same trial conditions as intervention; matched appearance; opioid-naïve and experienced | Oxymorphone ER 40 & 50 mg improved CPSI sleep scores vs. placebo; 10 mg not significant. Sleep improvement paralleled pain reduction. AEs included somnolence, nausea, constipation, dizziness |
| Kılıç, 2023 | Progressive Muscle Relaxation (PMR), guided training by nurse specialist + CD for self-practice at home | 6 weeks; performed twice daily (8–10 a.m. & 20–22 p.m.), each session 10–35 minutes | Training session + guideline booklet; reinforcement by weekly phone calls; face-to-face supervised PMR session at week 3; muscles systematically contracted/relaxed from toes → face | Usual care | 6 weeks | Patients completed the same assessments (PSQI, FSS) but no PMR training; received weekly phone calls only to check health status | PMR significantly improved sleep quality (PSQI) and reduced fatigue (FSS) compared with control. Improvements were clinically meaningful and maintained at 6 weeks |
| Kosinski, 2007 | Different dosages of Tramadol: Tramadol ER (100, 200, 300, 400 mg once daily, titrated over 15 days) | 12 weeks (2-week titration + 10-week treatment) | Patients with chronic hip/knee OA pain (ACR criteria). All had pain VAS ≥40 mm at baseline. Allowed stable use of glucosamine/chondroitin | Placebo | 12 weeks | Same conditions as intervention; matched appearance; all underwent washout of prior analgesics before randomization | Tramadol ER produced greater and clinically meaningful improvements in pain-related sleep disturbance (no matter the dosage) vs. placebo. Sleep benefit was correlated with pain relief |
| Latocha, 2023 | Group-based nurse-led CBT-I (2 h/week, 6 weeks, manual-based, in hospital) | 6 weeks + FU | // | Usual care | // | // | CBT-I did not improve PSG outcomes, but led to significant improvements in the insomnia index, PSQI scores, fatigue, depression, and RAID (Rheumatoid Arthritis Impact Disease) at week 26 |
| Lavie, 1991 | Tenoxicam 20 mg/day, oral NSAID, preceded by 3-7 days of NSAID washout and four days of placebo | 90 days | All-night PSG was performed in a sleep laboratory during the last 2 days of placebo treatment and on days 13, 14, 89, and 90 of tenoxicam treatment | NA | NA | NA | Tenoxicam improved clinical RA outcomes but did not improve sleep. 8/13 patients (62%) had primary sleep disorders (PLMs, sleep apnea). Patients with sleep apnea showed poorest clinical response |
| LeBrun, 2024 | Oral melatonin 5 mg nightly | 6 weeks after TKA | // | Oral vitamin C 125 mg (placebo) nightly | 6 weeks after TKA | // | Melatonin did not improve subjective sleep quality or PROMs at 6 weeks or 90 days. Poor sleep quality associated with worse function and pain, but melatonin did not alter this |
| Lee, 2023 | Auricular acupressure at sleep/pain points (shenmen, subcortex, sympathetic, liver, heart), with vaccaria seed tapes, 5 days/week | 8 weeks | // | Placebo auricular acupressure at irrelevant points (upper/lower tragus, eyes, duodenum, LI) | 8 weeks | // | Auricular acupressure significantly improved subjective sleep quality, SE, latency, awakenings, N2 sleep, melatonin, and pain outcomes in older adults with OA living in nursing homes, compared to placebo |
| Leigh, 1989 | Tenoxicam 20 mg nocte (oral NSAID) | 3 weeks (after 1-week washout) | All patients also received paracetamol 500 mg QID for pain | Placebo (oral tablets) | 3 weeks | Crossover after washout (1 week); matched placebo | In men with OA, tenoxicam did not improve subjective (LSEQ) or objective sleep outcomes (PSG) |
| Lerman, 2017 | CBT-I, weekly sessions | 8 weeks | // | Behavioral Desensitization (active placebo, relaxation-like control) | 8 weeks | Not sleep-focussed | Both CBT-I and Behavioural Desensitization significantly reduced trait, daytime, and nocturnal pain-catastrophizing in KOA with insomnia. Early reduction in WASO predicted later reductions in catastrophizing, highlighting sleep continuity as a mechanism. Gains were maintained at 6 months |
| Londhe, 2025 | Robotic-assisted TKA, spinal anaesthesia with Adductor Canal Block, multimodal analgesia, standardized physiotherapy and rehab protocol | // | // | Conventional TKA, same anaesthesia, implant type, pain management and rehab protocol as IG | // | // | RATKA group had significantly better sleep quality at 8 weeks (PSQI 5.7 ± 0.7 vs 6.3 ± 0.9 in CTKA; p=0.0057, Cohen’s d=0.68). |
| Løppenthin, 2014 | Intermittent aerobic exercise (cycle ergometer) | 18 sessions over 6–8 weeks; 2–3 sessions/week; 20–30 min/session | High-intensity intervals alternating with recovery; supervised sessions | Usual care | // | // | Protocol paper – trial registered (NCT01966835); no results reported. |
| Lü, 2017 | Baduanjin exercise | 12 weeks, 3 sessions/week, 40 min/session | Group-based, led by certified instructor | Usual care | // | // | Baduanjin significantly improved PSQI global score, sleep efficiency, and sleep duration vs control at 12 weeks; also reduced pain and improved physical function |
| Lu, 2019 | Auricular plaster therapy with Codonopsis seeds; acupoints based on TCM syndrome differentiation | 1 month; patients pressed seeds 4–5 times/day, including 30 min before bedtime | // | Oral estazolam 1 mg nightly before bedtime | // | // | Both groups improved, but auricular plaster group showed greater reductions in PSQI dimensions (sleep quality, latency, efficiency, daytime dysfunction) and AIS scores compared to estazolam (p<0.05). |
| Lu et al., 2023 | Self-aromatherapy hand massage with 5% compound (Cedarwood Atlas, Sweet Marjoram, Sweet Orange in almond oil) | 3 weeks; 10 min/session; 3 sessions/week | Guided by manual + video, with weekly monitoring | Placebo massage (sweet almond oil only) and control (no intervention) | 3 weeks (placebo); 3 weeks (control) | NA | Intervention group had significant reduction in PSQI after 1 week vs control (B = -1.19, p = 0.046); both intervention and placebo improved PSQI and ESS within-group across 3 weeks; no between-group difference in pain. |
| Manning, 2017 | THA or TKA | // | // | NA | NA | NA | Early postoperative period (~5 weeks): worsened sleep with increased sleep latency and nocturnal awakenings. At 10 months: sleep quality and daytime sleepiness improved compared with baseline. Suggests a transient negative effect of surgery on sleep, followed by recovery |
| McCurry, 2021 | Telephone-delivered CBT-I | 6 sessions over 8 weeks; 20–30 min/session | Included components on sleep restriction, stimulus control, cognitive restructuring, and sleep hygiene; delivered by trained clinicians | Education-Only Control | Same as CBT-I | Telephone-delivered educational sessions on sleep and OA management, social support, and lifestyle information; no behavioural or cognitive components | Telephone CBT-I was effective in improving sleep, fatigue, and, to a lesser degree, pain. Results support provision of telephone CBT-I as an accessible, individualized, effective, and scalable insomnia treatment |
| McKenna, 2021 | Walking-based physical activity intervention | 8 weeks; 28 sessions (2–5 times/week); one supervised session weekly | Home sessions supported by activity diaries | Exercise advice only | 8 weeks | // | Walking intervention improved PSQI and sleep duration vs advice-only; feasible and safe |
| Mehta, 2025 | Primary TKA with posterior stabilized prosthesis and patellar resurfacing; standardized analgesic protocol (regional block + multimodal pain management); standard postoperative rehabilitation | // | // | NA | NA | NA | Pre-op mean PSQI 9.23 (SD 3.03); worsened at 6 wks (12.58), improved by 1 yr (5.69). 91% poor sleepers pre-op; 51% still poor at 1 yr. Sleep improvement greater in non-obese (61%) vs obese (0%), and non-diabetics (55%) vs diabetics (8%). BMI was a significant predictor of poor sleep at 1 yr (β=0.43, p<0.001). Functional outcomes (OKS, KSS) improved markedly and correlated moderately with sleep quality. |
| Oksüz, 2021 | Group 1: aerobic training + CPE Group 2: aerobic training | 8 weeks, 3days/weeks | // | NA | NA | NA | In group 1, significant improvements were observed in disease activity (BASDAI), mobility (BASMI, BST, functional reach, 6MWT), function (BASFI), muscle strength, QoL (ASQoL), fatigue (MAF), and sleep quality (PSQI). In group 2, significant improvements were limited to BASDAI, chair sit-to-stand, 6MWT, and ASQoL. Overall, adding CPE to aerobic training was more effective in improving functional, psychosocial outcomes, and sleep quality in patients with AS |
| Roehrs, 2015 | Sleep restriction (4 h time-in-bed, 3 am–7 am) followed by MSLT and recovery sleep (8 h, 11 pm–7 am) with polysomnography | // | // | Baseline 8 h TIB (PSG + MSLT), RA and HC groups | // | // | After bedtime reduction, all groups had shorter MSLT latencies (greater sleepiness). On recovery night, FM patients increased sleep efficiency (84%→88%) and showed REM rebound (21.6%→25.2%), unlike RA and HC. RA patients failed to increase recovery sleep efficiency (84%→81%), suggesting joint pain disrupted restorative sleep |
| Rosenthal, 2007 | Once-daily extended-release morphine sulfate (AVINZA®, A-MQD) | 14–20 days | Morning dosing (30 mg/day, titrated to 60 mg/day if inadequate pain relief after 6 days); no rescue pain/sleep meds allowed | Single-blind placebo lead-in used as within-subject comparator; prior pain medications withdrawn | 5 days (placebo run-in) | // | A-MQD improved sleep continuity (↑TST, ↑SE, ↓WASO) and preserved sleep architecture with slight REM reduction but reduced REM latency. Subjective sleep quality and hours slept improved; pain (BPI) decreased significantly. Neurocognitive tests also improved. A-MQD generally well tolerated (mild nausea, sedation, constipation) |
| Roth, 2009 | Eszopiclone 3mg capsules | 4 weeks, once/day + 2 weeks placebo run out | // | Placebo | 4 weeks | // | Eszopiclone 3 mg improved all assessed sleep and daytime function measures over the treatment period, as well as some measures of RA-associated pain, disability, and QoL |
| Salwen, 2017 | 8-session multicomponent CBT-I | 8 weeks | // | Active behavioural desensitization control condition (BD) | 8 weeks | // | Clinically significant pain reduction (≥30% at 6 months, N=31 responders) was predicted only by mid-treatment TST. A cut-off of ~382 minutes (≈6.5 h) best predicted pain improvement; findings were consistent for diary and actigraphy. Other sleep continuity measures (SE, SL, WASO) were not predictive. Early increases in TST during CBT-I or BD may drive long-term pain reduction |
| Shakya, 2019 | Zolpidem 10 mg + perioperative sleep care education | 2 days preoperative to 5 days postoperative (30min prior to  going to bed) | // | Placebo + perioperative sleep care education | Same as IG | // | Sleep quality is impaired both before and after THA surgery to varying degrees. Short-term application of Zolpidem can improve the quality of peri-op sleep. It reduces perioperative nighttime, walking, rest, and pain, effective in alleviating pain resulting less doses of opioids |
| Shourabi, 2024 | Self-administered acupressure (ST34, ST36, SP9, SP10, GB34) | 1 month (daily before bedtime, 1 min per acupoint) | Participants trained individually by certified researcher; practiced at home using provided guide; weekly FU calls for adherence; continued routine medical care | Group 1: sham acupressure (ST40, SP6, LV3, non-acupoints)  Group 2: control (routine care only) | 1 month | // | Self-administered acupressure as a non-pharmacological approach may be a helpful tool in enhancing sleep quality for individuals with KOA |
| Straub, 2017 | Golimumab (50 mg or 100 mg, subcutaneous, every 4 weeks) with or without MTX, compared to placebo + MTX | 52 weeks (1 year) | // | NA | NA | NA | Inflammation control leads to a positive interrelation of serum cortisol and sleep parameters. This investigation could be a hypothesis-generating forerunner for similar studies that examine HPA axis hormones and feedback in RA patients by making use of sleep parameters, cytokines, and HPA axis hormones |
| Suhas Masilamani, 2025 | Robotic-assisted total knee arthroplasty (RATKA, MAKO® system) | // | // | Conventional TKA (same surgeon and peri-operative care) | // | // | Both RATKA and conventional TKA resulted in similar patterns of postoperative sleep disturbances, peaking shortly after surgery and progressively improving over 6 months without fully returning to pre-operative levels. Despite significantly better pain control in the RATKA cohort, postoperative sleep quality did not differ significantly between the two surgical techniques |
| Sieper, 2015 | Group 1: CZP 200 mg every 2 weeks Group 2: CZP 400 mg every 4 weeks Both after 3 loading doses (400 mg at weeks 0, 2, 4**)** | 24 weeks | // | Placebo | Same schedule as IG | // | At week 12 and 24, patients treated with CZP showed significant improvements in MOS-SPI-II scores compared with placebo, indicating reduced sleep problems. Sleep benefits paralleled improvements in pain, fatigue, and overall disease activity |
| Sveaas, 2020 | High-intensity cardiorespiratory (40 min; intervals at 90–95% HRmax) and strength (8–10 reps, 2–3 sets of major muscle groups) exercise | 3 months, 3 sessions/week | Two supervised sessions per week by physiotherapists and one home-based session | Usual care | 3 months | // | High-intensity cardiorespiratory and strength exercise program has a short-term beneficial effect on fatigue, vitality, sleep, and mood in patients with axSpA, but as expected, long-term effects were not seen |
| Taibi, 2009 | 600 mg valerian (Valeriana officinalis) | 5 nights | // | Placebo | 5 nights | // | Sleep disturbance is a common symptom in chronic conditions, including arthritis, and treatment should include direct care of sleep disturbance Persons with arthritis-related sleep disturbance show interest in using herbs, but little evidence exists on the efficacy of herbal products for improving sleep in this population |
| Taibi, 2011 | Yoga classes, each started with relaxation and body awareness, and then progressed to seated poses, supine poses, breathing exercises, and deep relaxation. | 8 weeks (including 75-min weekly classes and 20 min of nightly home practice) | // | NA | NA | NA | Women with OA and sleep disturbance were highly interested in yoga as a potential treatment for their symptoms. Efficacy findings from the study were promising, showing significant improvements in several self-report sleep outcomes |
| Tang, 2021 | 30-minute pulsing light (goggles) and pulsing binaural sounds (headphones). The active AVS protocol consisted of stimulation from 10 to 6 Hz during the first 3 minutes, then 6 to 3 Hz at minutes 4-28, and 3 to 2 Hz at minutes 29-30 | 2 weeks | // | 30 minutes of constant dim light (goggles) that slowly changed in colour, and a steady monotone (headphones) at an ultra-low frequency (below 1 Hz) out of entrainment range. The placebo control AVS protocol consisted of stimulation from 1 to 0.4 Hz during the first 3 minutes, then 0.4 to 0.2 Hz at minutes 4–6, and 0.2 Hz at minutes 7–30 | 2 weeks | // | Active 30-minute AVS program has the potential to improve sleep in older adults with OA pain and sleep onset insomnia. The advantages of the AVS intervention include its short nightly delivery time (30 minutes), its non-pharmacological nature, its acceptability to users, and the fact that it can be readily used by individuals at home. Subjects were highly adherent to the protocol, rated the AVS device easy to use, and said they would be likely to continue to use it in the future. |
| Taylor-Gjevre, 2011b | anti-TNF-a therapy | PSG assessment in one overnight at two times | // | NA | NA | NA | Observed improvement in the objective PSG parameters of sleep efficiency and awakenings following initiation of anti-TNF-a therapy. These results lend support to the hypothesis that TNF-a plays a role in mediation of sleep health in RA patients. |
| Vitiello, 2014 | Group 1: CBT for pain and insomnia (CBT-PI)  Group 2: CBT for pain alone (CBT-P) | 6 weeks, 90–120 min/week | Group-based interventions in primary care clinics. CBT-PI integrated pain coping and insomnia components. CBT-P included only pain coping strategies. Conducted by trained therapists. | Education-Only Control | 6 weeks | Attention control group with educational sessions on pain and sleep health, but without behavioral or cognitive skills training | FU analyses showed that participants who achieved ≥30% short-term improvement in sleep had sustained 9–18-month improvements in sleep quality, pain, fatigue, and arthritis symptoms, regardless of treatment arm. Improvements in sleep preceded and predicted later improvements in pain and fatigue, suggesting that better sleep mediates long-term pain relief |
| Vitiello, 2009 | CBT-I | 8 weekly 2-hour classes | Class sizes ranged from 4 to 8 participants, averaging 5 members per class | Stress management and wellness (not including an active treatment for relaxation  or specifically mention pain control) | Same as the IG | // | In addition to improving sleep, and even without directly addressing pain management, CBT-I appears to decrease pain in older patients with OA and co-morbid insomnia both after treatment and at 1-year FU |
| Vitiello, 2022 | Telephone-delivered  CBT-I | 8 weeks (6 sessions) | Conducted by trained therapists for older adults (≥60 years) with comorbid OA pain and insomnia. | Telephone-delivered education program on sleep and pain | 8 weeks | // | Sleep or pain improvers (≥30% reduction in ISI or BPI at 2 months) showed sustained 12-month improvements in insomnia, sleep quality, pain, depression, and fatigue. Sleep improvement predicted long-term benefits in pain, depression, and fatigue; effects small to moderate but significant |
| Von Koff, 2012 | Group 1: CBT for pain and insomnia (CBT-PI)  Group 2: CBT for pain alone (CBT-P) | 6 weeks, 90–120 min/week | Group-based interventions in primary care clinics. CBT-PI integrated pain coping and insomnia components. CBT-P included only pain coping strategies. Conducted by trained therapists. | Education-Only Control (EOC) | 6 weeks | Attention control group with educational sessions on pain and sleep health, but without behavioral or cognitive skills training | CBT-PI significantly improved insomnia severity and sleep efficiency vs EOC, with modest effects on pain |
| Ward, 2018 | Yoga classes + home practice | 3 months, one 75‐min class/week, (+20-min home practice) | 5‐min check-in, 5‐min introduction to the class lesson and yoga philosophy theme, 7‐min centring and breathing practice; 10‐min warm‐up practice, 28 min session of supine, seated and standing yoga postures (5 − 10 postures per class); 15‐min guided relaxation and 5‐min closing discussion | Usual care | // | // | An 8‐week relaxation‐focused yoga programme, designed with reference to Delphi recommendations of yoga interventions for musculoskeletal conditions, was feasible and safe to deliver in an RA population experiencing mild pain, mild to moderate functional disability and moderate disease activity. Some participants depended on the use of specialized equipment and props to achieve certain yoga postures within the constraints of their physical disability |
| Yousefi, 2023 | Group 1: MBSR Group 2: CBT | MBSR: 8 weekly 90-min group sessions CBT: 10 sessions, duration NR | // | Usual care | // | // | Both interventions improved sleep, fatigue, and executive function compared to usual care, with effects maintained at 3 month-FU. No impact was seen on disease activity |
| Zamarrón, 2004 | Infliximab infusion 3 mg/kg | Over 2 hours after the initial baseline measurements | // | NA | NA | NA | After the first infliximab infusion, patients showed better SE, shorter SL, more REM sleep, and fewer arousals, with improved alertness. Joint symptoms did not change, suggesting a central effect rather than pain relief |
| Zhang, 2021 | Thalidomide at a dose of 150 mg/day | 6 months, once/daily | // | NA | NA | NA | Thalidomide for 6 months reduced pain, disease activity, depression, anxiety, and sleep problems in refractory AS, with further improvement at 6 months. Side effects were mild and included drowsiness, dry mouth, constipation, dandruff, and dizziness |
| Zhu, 2023 | Unicompartmental knee arthroplasty | // | // | NA | NA | NA | Patients with better pre-op sleep had less post-op pain, lower opioid use, smaller blood changes, faster knee flexion recovery, and shorter hospital stay. Good sleep quality was linked to better prognosis and faster rehabilitation |

AEs, Adverse Events; AIMS-2, Arthritis Impact Measurement Scales 2; AS, Ankylosing Spondylitis; ASAS20, Assessment of Spondyloarthritis International Society 20% improvement criteria; ASQoL, Ankylosing Spondylitis Quality of Life questionnaire; AVS, Audio-Visual Stimulation; BASDAI, Bath Ankylosing Spondylitis Disease Activity Index; BASFI, Bath Ankylosing Spondylitis Functional Index; BASMI, Bath Ankylosing Spondylitis Metrology Index; BEO, Bitter Orange Essential Oil; BKZ, Bimekizumab; BPI, Brief Pain Inventory; CBT, Cognitive Behavioural Therapy; CBT-I, Cognitive Behavioural Therapy for Insomnia; CBT-P, Cognitive Behavioural Therapy for Pain; CBT-PI, Cognitive Behavioural Therapy for Pain and Insomnia; CG, Control Group; CRP, C-reactive protein; CZP, Certolizumab Pegol; DAS-28, Disease Activity Score on 28 joints; DMARDs, Disease-Modifying Antirheumatic Drugs; EEG, Electroencephalogram; EOC, Education-Only Control; ESS, Epworth Sleepiness Scale; ETA, Etanercept; FFS, Flinders Fatigue Scale; FM, Fibromyalgia; FSS, Fatigue Severity Scale; FU, Follow-Up; GPR, Global Postural Re-education; HC, Healthy Controls; HRQoL, Health-Related Quality of Life; IG, Intervention Group; IL-6, Interleukin-6; ISI, Insomnia Severity Index; JPRT, Joint Protection and Relaxation Training; JSEQ, Jenkins Sleep Evaluation Questionnaire; KSS, Knee Society Score; LSEQ, Leeds Sleep Evaluation Questionnaire; MAF, Multidimensional Assessment of Fatigue; MBSR, Mindfulness-Based Stress Reduction; MOS-SPI-II, Medical Outcomes Study Sleep Problem Index II; MTX, Methotrexate; NA, Not Applicable; NR, Not Reported; NSAIDs, Non-Steroidal Anti-Inflammatory Drugs; OA, Osteoarthritis; OKS, Oxford Knee Score; PHQ-7, Patient Health Questionnaire–7 items; PHQ-9, Patient Health Questionnaire–9 items; PMR, Progressive Muscle Relaxation; PP, Phonophoresis; PSQI, Pittsburgh Sleep Quality Index; QoL, Quality of Life; QW2/QW4/Q2W/Q4W, every 2 or 4 weeks; RAID, Rheumatoid Arthritis Impact of Disease score; RA, Rheumatoid Arthritis; RATKA, Robotic-Assisted Total Knee Arthroplasty; REM, Rapid Eye Movement sleep; SSc, Systemic Sclerosis; SE, Sleep Efficiency; SF-36, Short Form-36 Health Survey; SL, Sleep Latency; SLZ, Sulfasalazine; TENS, Transcutaneous Electrical Nerve Stimulation; THA, Total Hip Arthroplasty; TKA/TKR, Total Knee Arthroplasty/Replacement; TNF-α, Tumour Necrosis Factor-alpha; TNFi, Tumour Necrosis Factor inhibitor; TST, Total Sleep Time; US, Ultrasound therapy; WASO, Wake After Sleep Onset.
